# Global transcriptome analysis of allopolyploidization reveals large-scale repression of the D-subgenome in synthetic hexaploid wheat

**DOI:** 10.1038/s42003-023-04781-7

**Published:** 2023-04-17

**Authors:** Akshaya Vasudevan, Madeleine Lévesque-Lemay, Tara Edwards, Sylvie Cloutier

**Affiliations:** 1 Agriculture and Agri-Food Canada, Ottawa Research and Development Centre, Ottawa, ON Canada; 2 Department of Biology, University of Ottawa, Ottawa, ON Canada

**Keywords:** Genome duplication, Polyploidy in plants, Plant hybridization

## Abstract

Synthetic hexaploid wheat (SHW) lines are created as pre-breeding germplasm to diversify the D subgenome of hexaploid wheat and capitalize upon the untapped genetic diversity of the *Aegilops tauschii* gene pool. However, the phenotypes observed in the *Ae*. *tauschii* parents are not always recovered in the SHW lines, possibly due to inter-subgenome interactions. To elucidate this post-polyploidization genome reprogramming phenomenon, we performed RNA-seq of four SHW lines and their corresponding tetraploid and diploid parents, across ten tissues and three biological replicates. Homoeologue expression bias (HEB) analysis using more than 18,000 triads suggests massive suppression of homoeoalleles of the D subgenome in SHWs. Comparative transcriptome analysis of the whole-genome gene set further corroborated this finding. Alternative splicing analysis of the high-confidence genes indicates an additional layer of complexity where all five splice events are identified, and retained intron is predominant. Homoeologue expression upon resynthesis of hexaploid wheat has implications to the usage and handling of this germplasm in breeding as it relates to capturing the effects of epistatic interaction across subgenomes upon polyploidization. Special considerations must be given to this germplasm in pre-breeding activities to consider the extent of the inter-subgenome interactions on gene expression and their impact on traits for crop improvement.

## Abstract (web summary)

Substantial downregulation of D homoeologs is reported in synthetic hexaploid wheat lines, as a consequence of inter-subgenome interactions following polyploidization, impacting gene expression in pre-breeding lines involving synthetic hexaploids.

## Introduction

Whole-genome duplication events are one of the prime drivers of speciation^1–3^. The majority of angiosperms have undergone polyploidization in the course of their evolution, and notably, 30% of crop species are deemed to be polyploid based on the extent of duplicated loci in their genome^4^. The grass lineage underwent a minimum of three whole genome duplication events^5^, basically making the whole Poaceae family polyploid. Whole genome duplication events are frequently found to be associated with significant evolutionary potential that promotes adaptability to changing environments^6,7^. There are several intriguing questions related to polyploids, from the initial dynamic changes they undergo in the genome for stabilization, until their establishment as discrete populations^8^. Polyploidization creates extensive redundancy within the genome, thereby paving the way for novel alterations that promote the development of new phenotypes and/or adaptation^9^.

Bread wheat (*Triticum aestivum* L.), is an allohexaploid crop (2*n* = 6x = 42) comprised of the A, B and D subgenomes. The natural chance hybridizations that occurred ~8000 years ago between cultivated emmer wheat (*Triticum turgidum* L. ssp. *dicoccum*, 2*n* = 4x = 28; AABB genome) and Tausch’s goatgrass (*Aegilops tauschii* Coss., 2*n* = 2x = 14; DD genome), followed by chromosome doubling, resulted in the development of modern bread wheat^10,11^. Presumably, only a limited number of *Ae. tauschii* plants contributed to the evolution of hexaploid wheat, leading to an evolutionary bottleneck called the founder effect^12^; a bottleneck that was further constricted by limited natural flow of genetic variation from diploid to hexaploid species^13,14^ and subsequent domestication and breeding.

Wild relatives of crop species are a source of genetic diversity for multiple agriculturally important traits. However, there are certain limitations to their usage, including linkage drag, infertility and poor cross-compatibility^15^. Pre-breeding is a systematic approach that aims to recapture the genetic diversity of crop wild relatives and deploy it into breeding programs. Traditionally, the desired genomic segments from wild crop species are introgressed into elite cultivar backgrounds by repeated backcrossing with the support of state-of-the-art whole-genome genotyping tools for foreground and background selections. SHWs generated from the artificial hybridization between *T. turgidum* ssp. *durum* (AABB) or other subspecies and *Ae. tauschii* (DD), followed by chromosome doubling, serve as effective pre-breeding populations to broaden the genetic diversity of the D subgenome of hexaploid wheat. SHWs developed by the International Maize and Wheat Improvement Center (CIMMYT, Mexico)^16,17^ have been extensively utilized in wheat genetic diversity enrichment programs in several countries. Later, other research institutes and wheat improvement programs across the globe also produced SHWs using different sources of *Ae*. *tauschii* accessions to serve as base populations for commercial wheat breeding.

*Ae. tauschii* is a proven source of resistance to a broad range of biotic and abiotic stresses^18^. However, parental phenotypes are not always effectively recovered in the synthetic hexaploid lines, possibly due to the dynamic genetic and epigenetic changes and the consequent variation in gene expression^19^. For instance, studies spanning the past two decades have shown that resistance to stem rust pathogen (*Puccinia graminis* f. sp. *tritici*) is suppressed in the hexaploid state by Med15, a component of the Mediator complex encoded by the D subgenome^20,21^. Upon polyploidization, the D homoeologue of the *High-Affinity K* + *Transporter 1;5* (*HKT1;5*) shows reduced expression, when compared to diploid parental levels, but regains the diploid parent native expression level under salt stress condition^22^. In certain phenotypes, like grain width, each of the homoeologues is associated differently with the trait of interest and their combined levels of expression determine the resultant phenotype^23^.

The alteration across multiple layers of gene regulation upon polyploidization is also considered to improve adaptability of allohexaploid bread wheat when compared to its tetraploid and diploid parents^24,25^. The genetic and epigenetic alterations reported upon polyploidization in wheat are similar to other polyploid plant species. At the structural level, apart from homoeologous exchanges between genomes, both loss and amplification of repetitive DNA due to perceived genome stress have been observed^26^. The recent whole genome sequencing of bread wheat sheds light on the transposon insertions and deletions in the intergenic regions of the A, B and D subgenomes, although the fraction of each transposon family among the homoeologous genomes does not significantly vary^27^. At the epigenetic level, in addition to changes in cytosine methylation status of the DNA, variability in histone methylation^28,29^, euchromatin and heterochromatin patterns across chromosomes and prominent differences in the levels of epigenetic regulators like small RNAs were also observed^30,31^. These epigenetic marks on the regulatory elements of the genome modulate gene expression levels in hexaploid bread wheat^32^.

The effect of some of these genomic and epigenomic changes occurring upon polyploidization are reflected in the transcriptome. Understanding the pattern of expression changes between the parental ploidy backgrounds and hexaploid wheat provides valuable information to inform interspecific crosses and assist in predicting the phenotypes recovered in the progenies. In this study, we used four SHW lines and their corresponding tetraploid (*T. turgidum*) and diploid (*Ae. tauschii*) parents for a comparative evaluation of transcriptome dynamics. We focused on unraveling the changes in expression patterns of homoeologous genes between the subgenomes in parental (2x and 4x) and synthetic hybrid backgrounds (6x). In addition to quantitative differences, the subtle qualitative differences in transcripts arising from alternative splicing events were also determined.

## Results

### HEB in SHW lines

In this experiment, RNA-seq data was generated from four SHW lines and their two diploid and two tetraploid parents (Supplementary Table 1), from three biological replicates across ten tissues (Supplementary Fig. 1) for a total of 240 samples. After pre-processing of the sequencing reads for the 240 samples, we obtained a total of 4.9 B reads corresponding to an average of 20.4 M reads per sample, ranging from 4.8 to 70.7 M. The number of raw reads and processed reads for each sample was compiled (Supplementary Tables 2, 3). Approximately 85% of the total reads were uniquely mapped to the Chinese Spring IWGSC RefSeq v2.1^33^, ~12% mapped to multiple loci, and ~3% did not map at all (Supplementary Table 4).

**Fig. 1:**
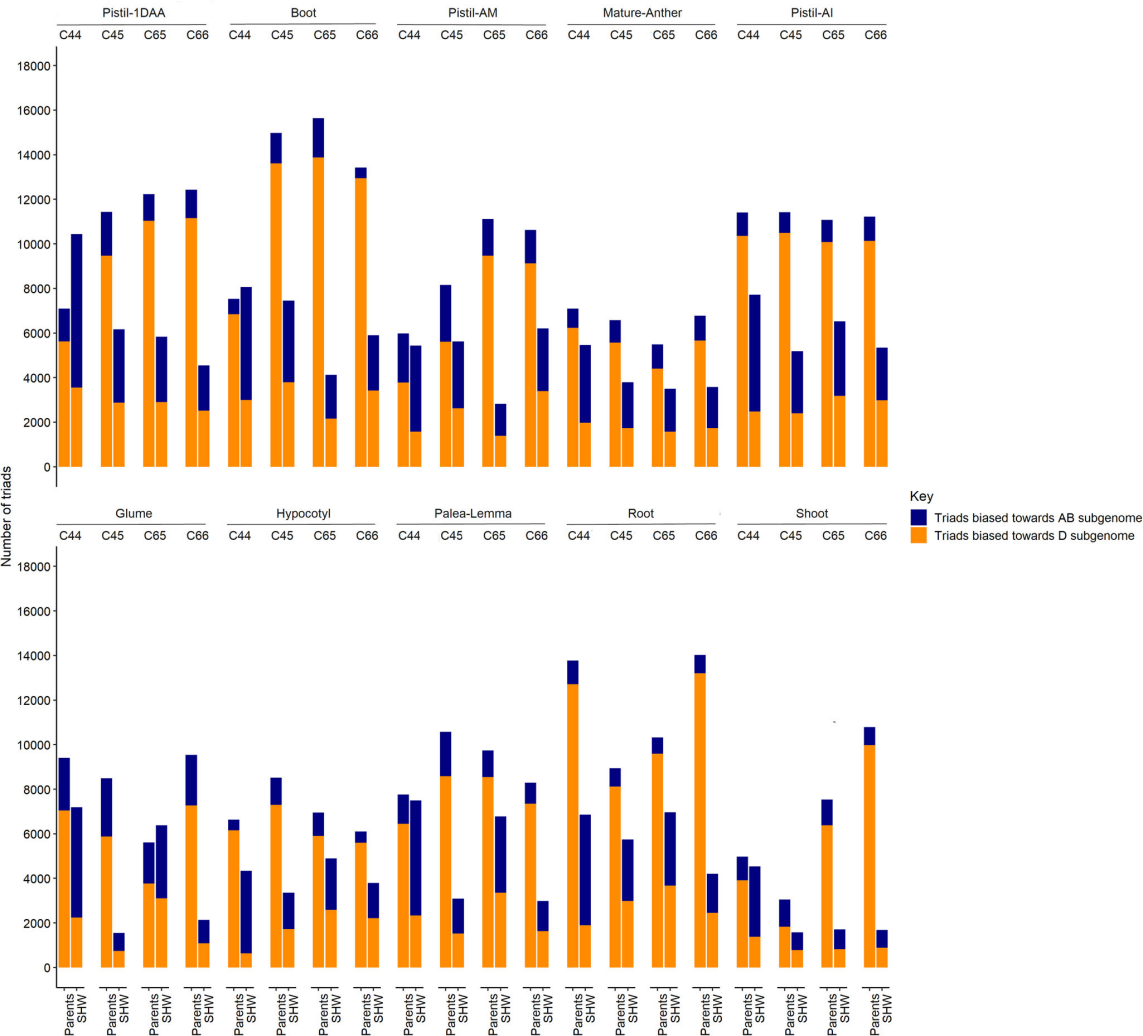
Number of triads showing significant bias towards AB and D subgenomes in all four SHW lines—C44, C45, C65 and C66 and in the natural state of expression in their parents. The navy blue and dark orange bars represent the number of triads showing significant expression bias towards AB and D subgenomes, respectively. Pistil-1DAA pistil-one day after anthesis, Pistil-AM pistil—when anthers are at mature stage, Pistil-AI pistil—when anthers are at immature stage, Boot head at boot stage.

In order to understand the global pattern of gene expression dynamics upon polyploidization, specifically, HEB, and compare them between SHWs and their parental state, genes (homoeologues) belonging to triads, i.e., with one copy in all three sub-genomes (18,357 triads^34^), were subjected to the likelihood ratio test^35^ (LRT) computation. Here, the differences in gene length between the homoeologues are considered. The null hypothesis, i.e., no expression differences between the homoeologues, was tested for all comparisons. In the SHW lines the AB vs. D homoeologues were compared. To test for the parental expression state, in-silico SHW-like scenarios with corresponding tetraploid and diploid parental expression levels were constituted. Expression biases, predominantly towards the D subgenome in parental level of expression, were observed across all ten tissues and SHWs (Fig. 1). The number of triads showing significant bias towards the subgenome contributed by the diploid (D) parent were drastically reduced in the corresponding SHW lines across all tissues (Fig. 1 and Supplementary Table 5). The pistil tissue when anthers were green and immature had more than 10,000 triads biased towards the D subgenome, based on parental expression levels in all four SHWs. The heads collected at the boot stage showed a similar pattern in all SHWs but C44 (Fig. 1).

**Fig. 2:**
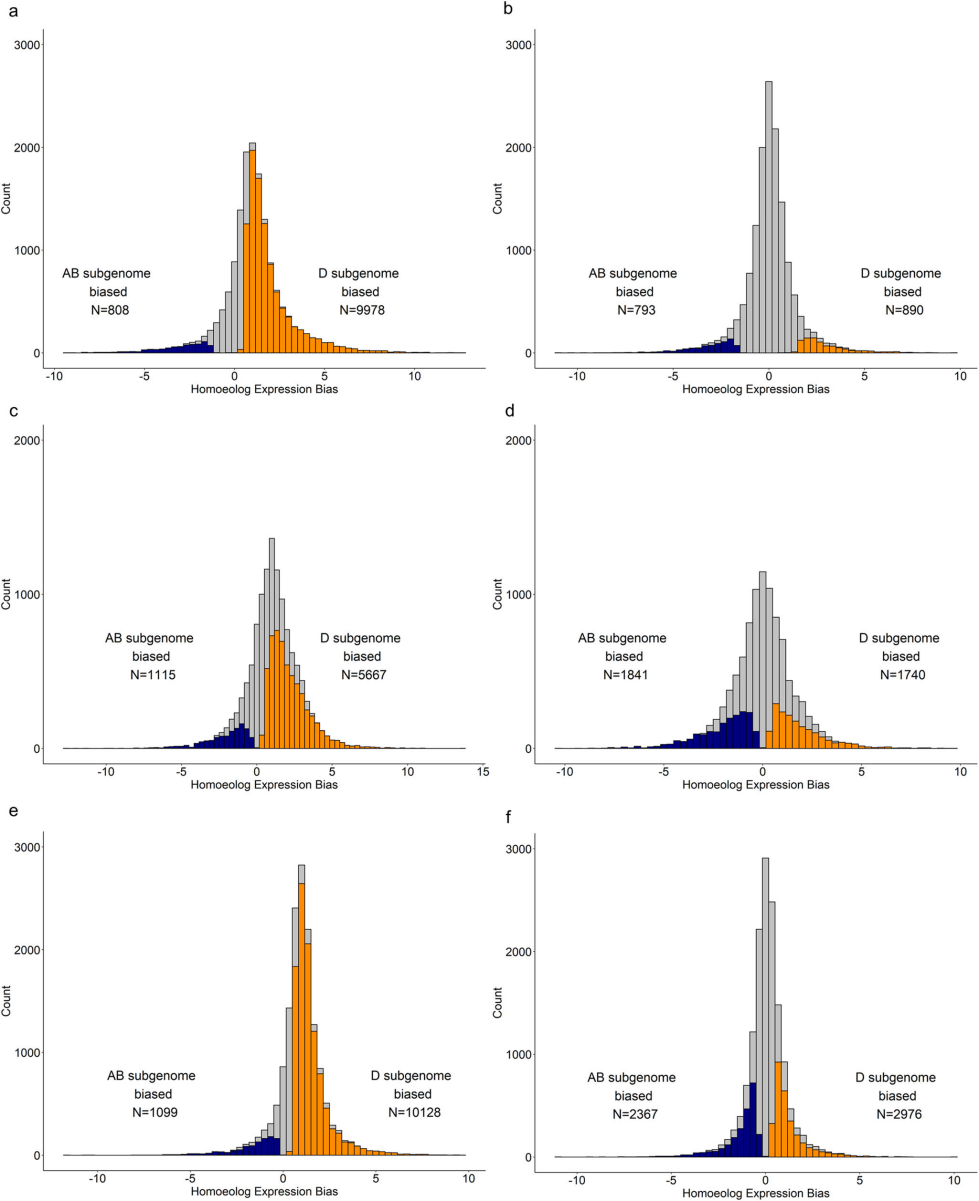
Distribution of homoeologue expression bias in SHW-C66. (**a**, **c**, **e**) Comparison of the expression bias of triads towards AB and D genomes of tetraploid (PI377655) and diploid (AS2386) parents; (**b**, **d**, **f**) Comparison of the expression bias of triads towards AB and D subgenomes of SHW-C66; (**a**, **b**) Shoot; (**c**, **d**) Mature anther just prior to dehiscence; (**e**, **f**) Pistil when anthers are green and immature. The navy blue and dark orange bars represent the triads showing significant expression bias towards AB and D subgenomes, respectively.

In the shoot tissue of SHW-C66, 9978 and 808 triads showed a significant bias towards the D and AB subgenomes, respectively, based on the parental level of expression. However, in the SHW background, only 890 triads were biased towards the D subgenome (Fig. 2a, b). In anthers, 5667 and 1115 triads were biased towards the D and AB subgenomes, respectively, in the progenitor level of expression and more balanced in the hexaploid condition with 1740 D-biased and 1841 AB-biased triads (Fig. 2c, d). Likewise, 10,128 triads biased towards the D subgenome in the parental expression levels were reduced to 2976 in the pistils (collected when anthers were green and immature) of SHWs (Fig. 2e, f). These results illustrate major shifts in expression bias among hundreds of gene sets, between the no-interaction scenario, i.e., the parental state of expression and the large-scale gene expression alteration caused by interactions of the subgenomes, i.e., in SHW lines. The number of triads significantly biased towards the D subgenome were higher in the parental genomic background, compared to their expression when they were introgressed into the synthetic hexaploid background. The distribution of the HEB for the other seven tissues for SHW-C66 is provided in Supplementary Figs. 2–8.

**Fig. 3:**
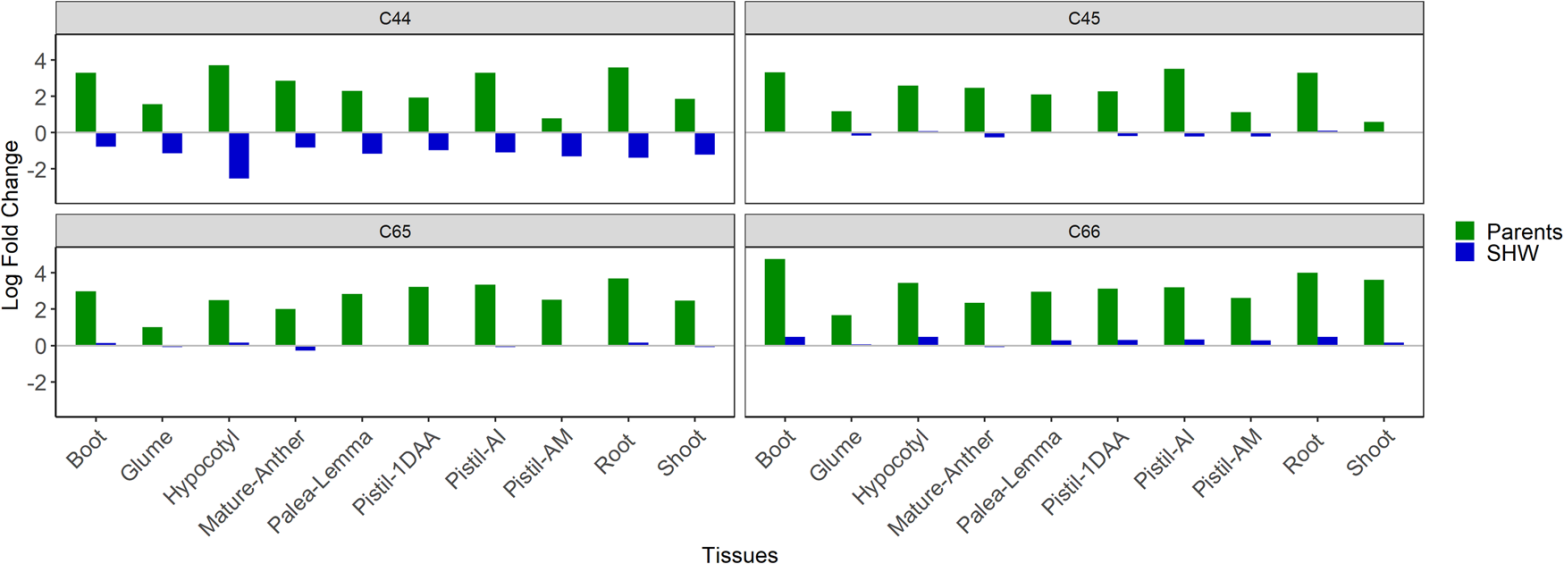
Comparison of ratios of triads significantly biased towards AB and D subgenomes within a genomic background (parents and SHWs). The ratios are represented as log fold change (LogFC) values. A LogFC < 0 indicates more triads are biased towards the AB subgenome than the D subgenome and a LogFC > 0 indicates more triads are biased towards the D subgenome than the AB subgenome, within a genomic background. C44, C45, C65 and C66 are the four SHWs taken for the study. The green bars represent the ratios in the parental genomic background and the blue bars represent the SHW genomic background. Pistil-1DAA pistil-one day after anthesis, Pistil-AM pistil-when anthers are at mature stage, Pistil-AI pistil-when anthers are at immature stage, Boot head at boot stage.

In the no-interaction predicted in-silico scenario, the proportion of triads biased towards the D genome were at least 1.5 fold (LogFC 0.6) higher than those biased towards the AB genome, as observed in shoot tissue of SHW-C45, and up to 27 fold (LogFC 4.8) higher, as observed in the head at boot stage tissue of SHW-C66 (Fig. 3). However, this number was drastically reduced in the actual hexaploid background and, an almost equal number of triads were biased towards the subgenomes of both parents (AB and D). On the contrary, the triads showing expression bias in the opposite direction, i.e., biased towards the AB genome increased in most tissue in the SHWs, with the most pronounced HEB being observed in SHW line C44 (Fig. 3).

**Fig. 4:**
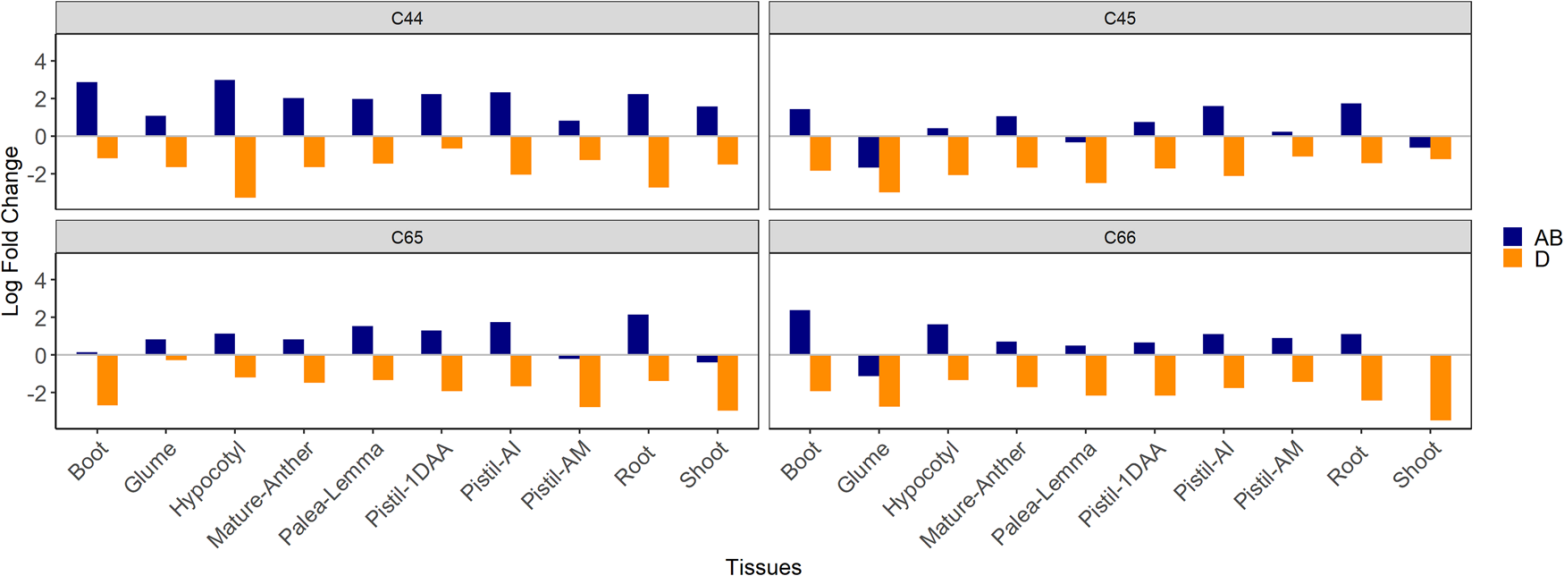
Comparison of ratios of triads significantly biased towards AB and D subgenomes between genomic backgrounds (parents vs SHWs). The ratios are presented as log fold change (LogFC) values. A LogFC > 0 indicates more triads are biased towards the AB subgenome in the SHW background than the parental background and a LogFC < 0 indicates more triads are biased towards the AB subgenome in the parental background than the SHW, similarly for D subgenome. C44, C45, C65 and C66 are the four SHWs used for the study. The navy blue and dark orange bars represent the triads showing significant expression bias towards AB and D subgenomes, respectively. Pistil-1DAA pistil-one day after anthesis, Pistil-AM pistil-when anthers are at mature stage, Pistil-AI pistil-when anthers are at immature stage, Boot head at boot stage.

The analysis of the trends within the same subgenome background revealed that the triads biased towards the AB subgenome were more numerous in the hexaploid state when compared to the parental state (Fig. 4). All tissues of SHW-C44 had an increased proportion of triads showing expression bias towards the AB subgenome, with up to 7.9-fold (LogFC 2.98) more triads overexpressed in the hypocotyl tissue. In the other three SHW lines, the increase in triads biased towards the AB subgenome were up to 5.2-fold (head at boot stage of C66; LogFC 2.37). The proportion of triads biased towards the D subgenome were reduced by more than eight times as detected across multiple SHW-tissue contexts.

The average magnitude of HEB values for the AB and D biased triads for the SHW and parental background scenarios are summarized in Supplementary Table 5. For the AB-biased triads, the magnitude of HEB values ranged from −1.17 (C65—pistil when anthers are green and immature) to −4.37 (C44—hypocotyl), in the SHW background. The magnitude of HEB of the AB biased triads varied from −1.22 (C65—head at boot stage) to −3.56 (C66—head at boot stage), in the parental background. Similarly, the magnitude of HEB values of the D-biased triads extended from 1.16 (C44—pistil when anthers are green and immature) to 3.12 (C45—glume), in SHW background, and from 1.32 (C45—head at boot stage) to 4.66 (C45—shoot), in parental background. The average magnitude of HEB values of the AB-biased triads were higher than those of the D-biased triads across all SHW-tissue scenarios, except in the C65 SHW background in pistil when anthers are green and immature. However, no fixed pattern was observed in the parental background (Supplementary Table 5).

### Role of parental genotypes on HEB

**Fig. 5:**
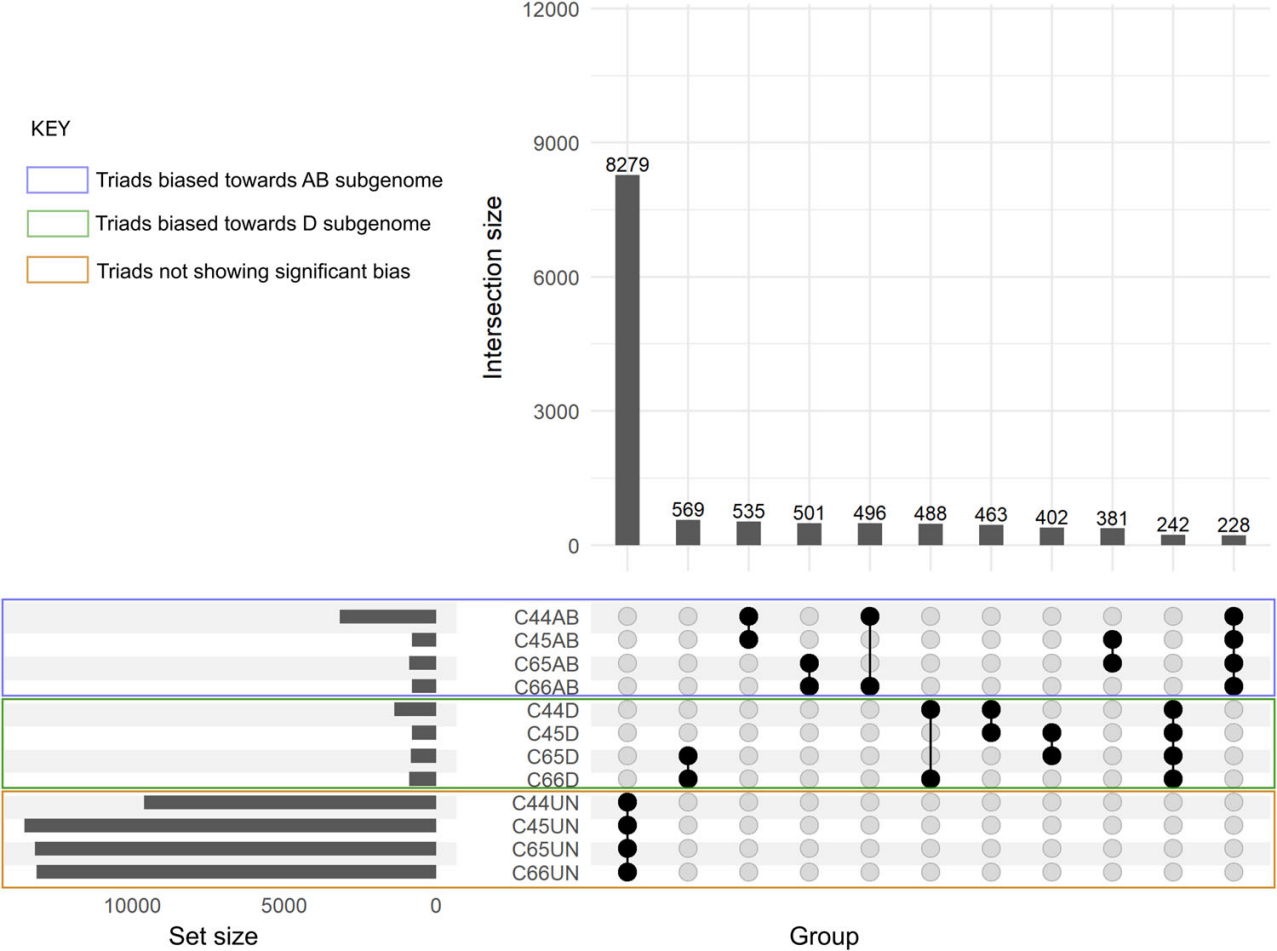
Subsets of triads showing similar expression pattern across all four SHW lines in shoot tissue. The gray horizontal bars under set size corresponding to C44AB indicates the number of triads biased towards the AB subgenome in the SHW-C44; C44D indicates the number of triads biased towards the D subgenome in the SHW-C44; C44UN indicates the number of triads showing no significant bias in the SHW-C44; Similarly, for SHWs C45, C65 and C66. The vertical bars under intersection size indicate the number of triads showing similar expression patterns between sets. The black dots highlight the two SHW sets compared for the corresponding intersection. The number over the bars represents the number of triads showing similar expression pattern. The triad sets showing significant bias towards the AB or D subgenomes and those not showing any significant bias are highlighted by the blue, green and brown rectangles, respectively.

The subset of triads showing similar expression bias patterns across different SHW lines was also analysed. The intersections among the four SHW lines and between the SHW lines sharing common tetraploid or diploid parents is represented in Fig. 5 for the shoot tissue. The largest intersection represented the subset showing no significant bias in all four SHW lines, and this was true across all ten tissues (Fig. 5; Supplementary Figs. 9–17). Shoot tissue had the smallest subset of triads showing similar significant expression bias towards the AB (228 triads) and D (242 triads) subgenomes, across the four SHW lines (Fig. 5). The largest intersection among the SHW lines was found in pistil when anthers are green and immature (Supplementary Fig. 15) and pistil–one day after anthesis, for the AB-biased (1281) and D-biased (1066) triads, respectively (Supplementary Fig. 9).

### Role of tissue of expression of homoeologues on HEB

Out of the 18,357 triads analysed for the expression pattern, 69% to 73% were not comprised of any tissue-specific homoeologues at all, in all four SHW lines (Supplementary Table 6). The next largest fractions were triads with all three homoeologues showing same tissue specific expression (8% to 11%) and triads with only one homoeologue showing tissue specificity with the other two being more broadly expressed (7% to 12%). Further, there were more triads with two homoeologues showing expression specificity towards the same tissue and one broadly expressed homoeologue, than one homoeologue alone displaying expression specificity in another tissue (Supplementary Table 6).

Similar constitution was observed in individual SHW-tissue contexts, most commonly, with only one of the three homoeologues being tissue specific or all three homoeologues showing tissue specificity (Supplementary Table 7). The triads with two of the three homoeologues alone showing tissue specificity were lowest in number across the ten tissues in all four SHW lines. In the root and mature anther tissues, the subset of triads comprised of all homoeologues showing tissue specificity were largest in all four SHW lines. Out of the larger subsets of triads with all three homoeologues showing same tissue specificity, 455 triads in the root were common among all four SHW lines. When the homoeologues of these triads were subjected to gene ontology analysis for understanding functional enrichment, biological process terms including regulation of root morphogenesis and regulation of root meristem growth were enriched in the root. In mature anthers, this number was 427 and the biological process terms including pollen germination, pollen sperm cell differentiation, pollen tube growth, and pollen wall assembly were enriched.

A large number of triads, comprised of either one, two or three tissue-specific homoeologues, showed no significant expression bias, similar to the observation in the entire triad dataset. When the triads showing AB- and D- bias and their tissue specificity patterns were interrogated specifically, a moderate strength of association with Cramer’s-V statistic ranging between 0.20 and 0.57 was observed between bias pattern and tissue specificity of the homoeologues in most tissues in all four SHW lines. For instance, more triads showed D-subgenome bias when D-homoeologue alone was tissue-specific, greater number of AB-biased triads were observed when A and B homoelogues showed tissue specificity, and more D-biased triads were observed when A and D or B and D homoeologues alone showed tissue specificity, in most SHW-tissue contexts. However, based on Chi-square test of independence, the association between expression bias and tissue specificity was significant only in mature anthers, root and hypocotyl tissues in all four SHW lines, and C66 showed significant association additionally in pistil-one day after anthesis, pistil-when anthers are green and immature and C44 displayed significant association in all tissues. Although the observed extent of associations were moderate, the relationship between homoeologues’ tissue specificity and expression bias cannot be dismissed.

### Impact of genome stabilization on HEB

**Fig. 6:**
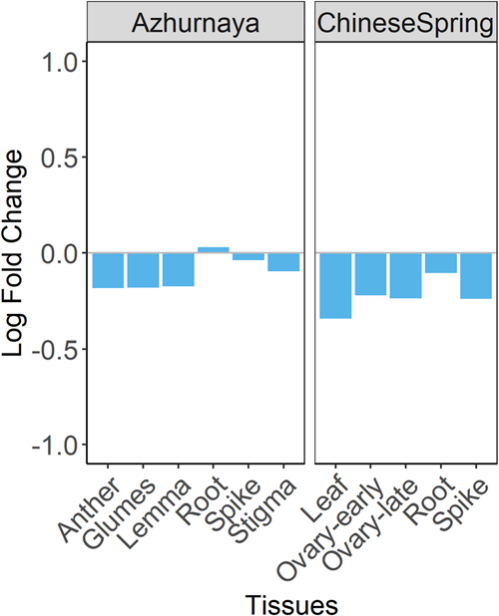
Ratio of triads significantly biased towards AB and D subgenomes in cultivar Azhurnaya and landrace Chinese Spring. The ratios are represented as log fold change (LogFC) values depicted by the blue bars. A LogFC < 0 indicates more triads are biased towards the AB subgenome than the D subgenome and a LogFC > 0 indicates more triads are biased towards the D subgenome than the AB subgenome.

To validate the observed patterns in more stabilized genomes, the expression bias was analyzed in wheat lines that were subjected to domestication and breeding processes using the publicly available RNA-seq data for the landrace Chinese Spring and the cultivar Azhurnaya from Ramírez-Gonzalez et al.^36^. The number of HEB and the magnitude of the expression biases were much smaller in these genotypes compared to the SHW lines (Fig. 6). In Azhurnaya, 2087 to 4244 triads were biased towards the AB subgenome and 2032 to 3739 triads were biased towards the D subgenome, in tissues reported in Fig. 6. In Chinese Spring, 874 to 4512 triads were biased towards the AB subgenome and 812 to 3821 triads were biased towards the D subgenome, across the five tissues used in the analysis. The magnitude of biases was lower in the two domesticated wheat lines compared to the newly created SHW lines.

The influence of domestication and breeding processes on the HEB patterns of the triads were analysed using the SHW, Chinese Spring and Azhurnaya results. Three of our SHW tissues could be matched to the developmental stages of samples in Ramírez-Gonzalez et al.^36^ namely pistils when anthers are mature, root and head at boot stage, and were used in this analysis. The majority of the triads (29–55%) maintained similar expression bias patterns in the resynthesized wheat, the landrace (Chinese Spring) and the cultivar (Azhurnaya) in all three tissues (Supplementary Fig. 18). Only 9–14% showed similar bias patterns in the SHW and Chinese Spring, and transitioned to a different bias state in Azhurnaya, possibly reflecting the impact of rigorous selection. Further, 9–30% of the triads, based on the different SHW-tissue scenarios, showed reversal to SHW bias state in Azhurnaya (Supplementary Fig. 18). Taking into consideration the HEB differences for the triads among the SHW lines, we also specifically investigated the subset of triads showing similar bias patterns across all four SHW lines. The major proportion of triads in this subset retained the same expression bias patterns in the SHWs, Chinese Spring and Azhurnaya, and the second largest proportion showed similar patterns in the SHWs and Azhurnaya and not in Chinese Spring, indicating the reversal of bias patterns during crop improvement. The triad set showing similar pattern changes including Chinese Spring and Azhurnaya was the smallest.

**Fig. 7:**
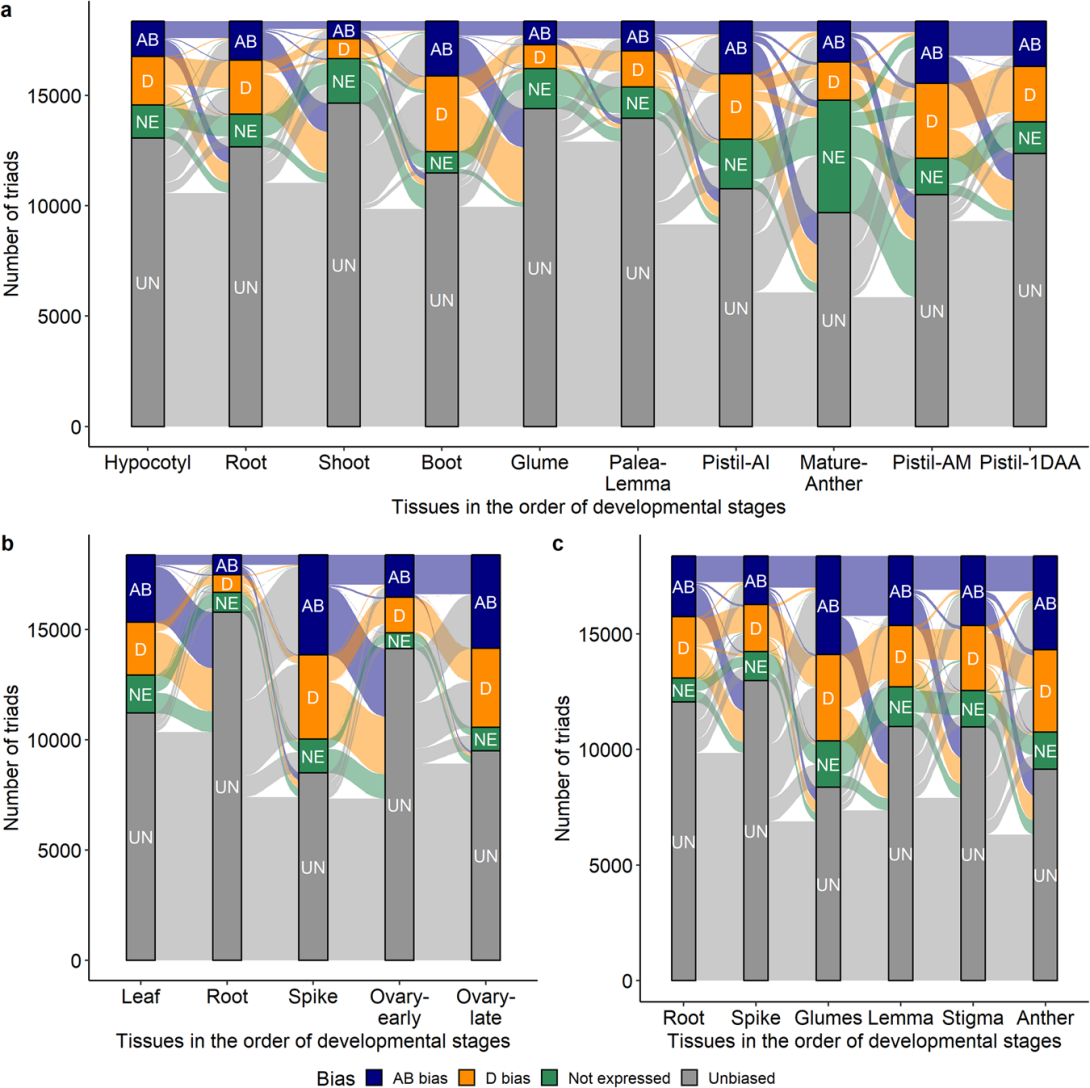
Expression bias trends of the triads across tissues. Alluvial plots representing the expression bias trends of the triads across the tissues in the (**a**) SHW-C66 (**b**) Landrace Chinese Spring and (**c**) Cultivar Azhurnaya; AB bias (AB), represented by navy blue bars: triads significantly biased towards the AB subgenome; D bias (D), represented by dark orange bars: triads significantly biased towards the D subgenome; Not expressed (NE), represented by green bars: the homoeologues of the triads are not expressed; Unbiased (UN), represented by gray bars: no significant expression bias observed.

The HEB expression analysis across ten different tissues enabled the investigation of bias patterns of the triads across tissues, i.e., through the developmental stages. In all four SHW lines, the largest pattern observed was triads showing no significant bias across any tissues. There were anywhere from 1449 to 3014 triads in the SHW lines that showed this pattern (Fig. 7, Supplementary Fig. 19). In all four SHW lines, the next most commonly observed bias trend was where the triads did not show significant bias in any of the tissues but were biased towards the AB or D subgenomes only in the mature anther tissues (Fig. 7, Supplementary Fig. 19). Patterns showing only significant bias in one of the tissues such as pistil—one day after anthesis, pistil when anthers are green and immature and root, were observed in multiple triads. In addition, there were thousands of triad-specific patterns across tissues. Similar trends were also observed in Azhurnaya, and the subset of triads showing significant bias in anther tissues was larger, whereas in Chinese Spring, triads showing significant bias only in spike tissue were common.

### Differential expression analysis of complete gene set

**Fig. 8:**
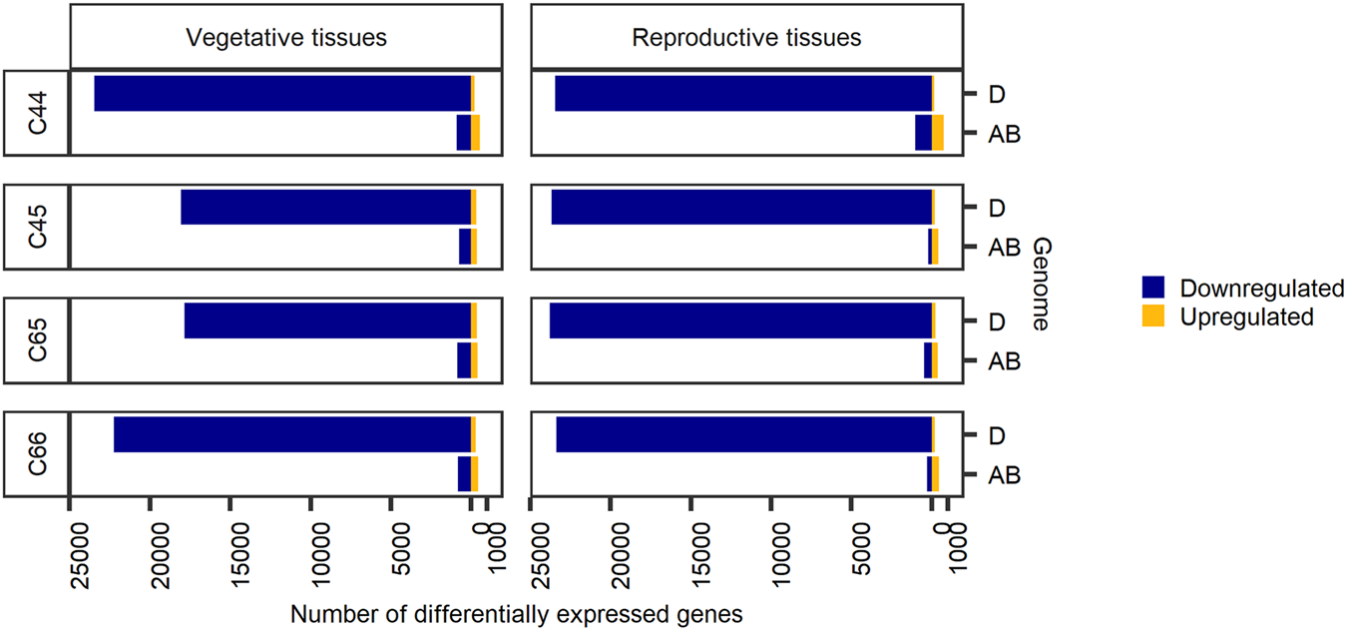
Differentially expressed genes of the AB and D subgenomes of the vegetative and reproductive tissue pools upon comparison of the SHW lines (C44, C45, C65 and C66) with their parents. The blue bars represent the number of downregulated genes and the yellow bars represent the number of upregulated genes.

In addition, the differential expression analysis of the whole gene set between the parents and SHWs was investigated. The 106,913 high confidence gene models identified in the hexaploid wheat genome as per the IWGSC RefSeq annotation v2.1^33^ were used in the analysis. In the genes from the A and B subgenomes, only a few hundred to a maximum of 1015 genes were downregulated in the SHWs compared to their tetraploid parents in SHW-C44 (Fig. 8). In contrast, more than 20,000 D subgenome genes were downregulated in all SHWs compared to their diploid parents (Fig. 8). Most importantly, in all three subgenomes, only 357 to 755 genes and 141 to 372 genes exhibited upregulation in the hexaploid state, in the AB and D subgenomes, respectively.

### Qualitative differences in transcripts between parents and SHWs

**Fig. 9:**
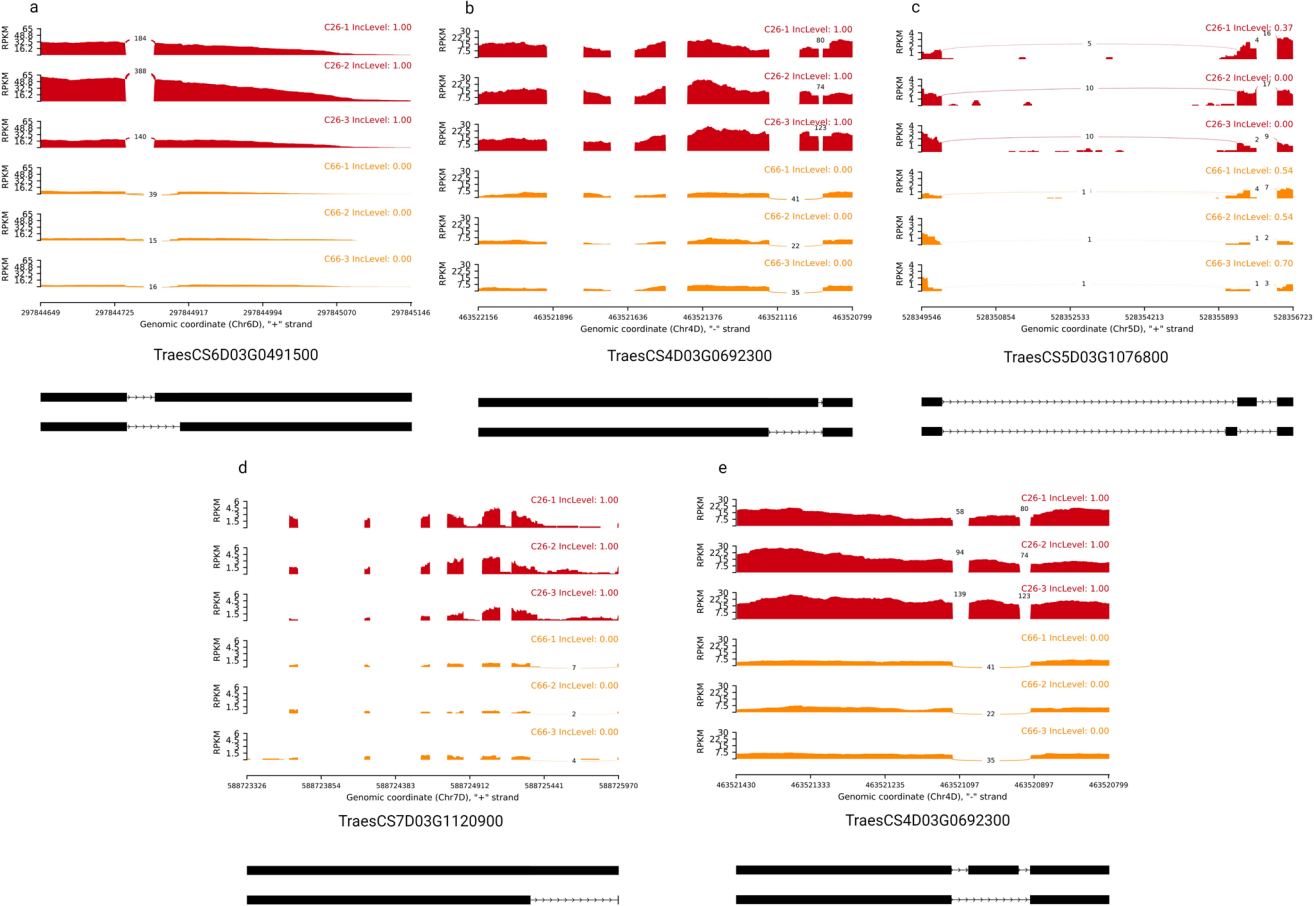
Sashimi plots representing examples of the five types of alternative splicing events in the shoot tissue of *Ae. tauschii* C26 line (diploid parent) vs SHW line C66. (**a**) Alternative 3′ splicing site; (**b**) Alternative 5’ splicing site; (**c**) Mutually exclusive exons; (**d**) Retained intron; (**e**) Skipped exon. The red plots represent the *Ae. tauschii* C26 parent transcripts and the yellow plots represent the SHW line C66 transcripts.

**Fig. 10:**
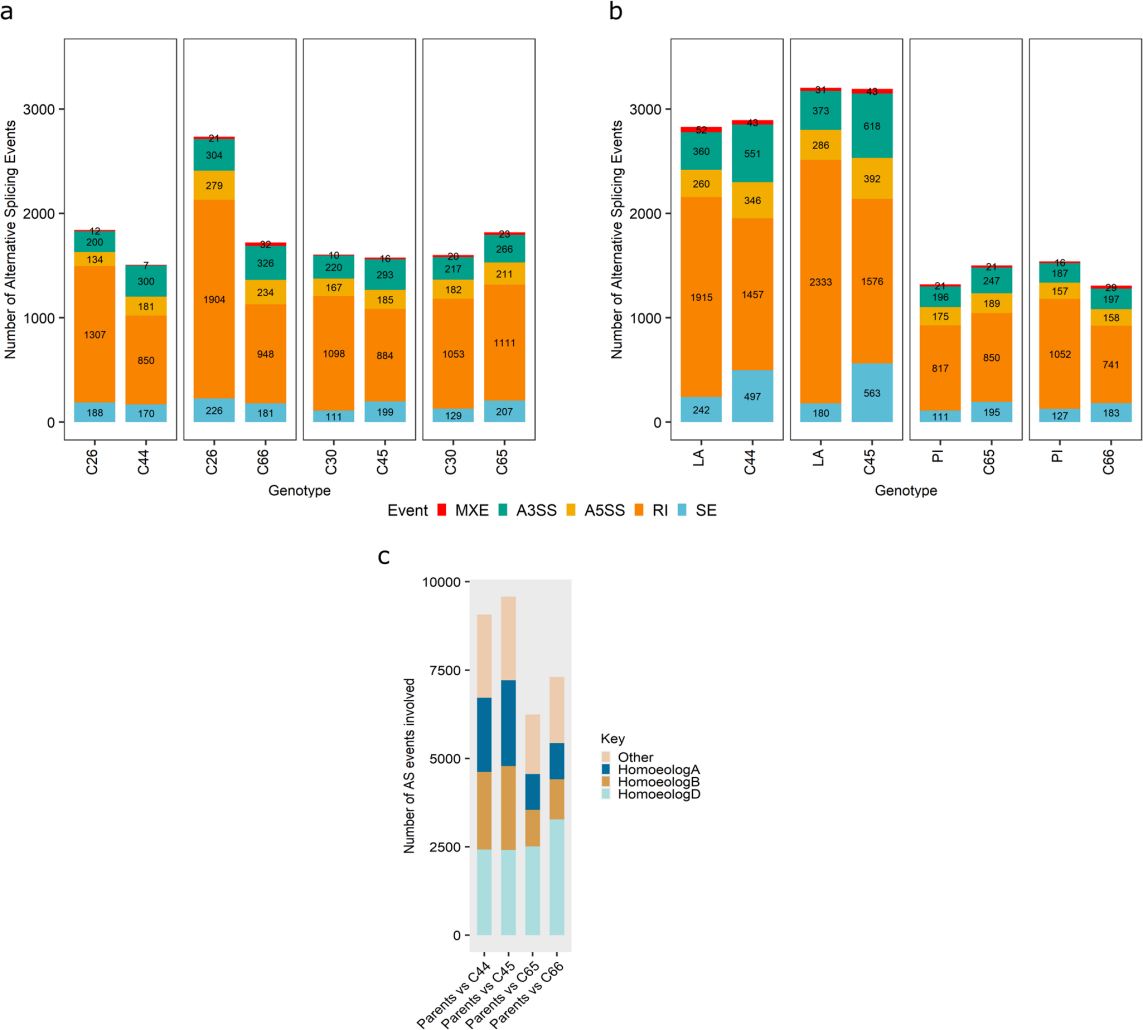
Number of alternative splicing events detected in the diploid (C26, C30)/tetraploid (LA, PI) parents compared to the SHWs (C44, C45, C65, C66) in shoot tissue. (**a**) Diploid parent vs. SHW line; (**b**) Tetraploid parent vs. SHW. MXE mutually exclusive exons, A3SS alternative 3′ splicing, A5SS alternative 5′ splicing, RI retained intron, SE skipped exon, LA Langdon, PI PI377655; (**c**) Proportion of alternative splicing events involving the homoeologues in triads and other genes.

The RNA-seq data was also mined to characterize the differences in splicing patterns at the different ploidy levels to shed light on the qualitative differences among transcripts. This analysis captured the splice variants from all genes whether differentially-expressed or not. Five major types of alternative splicing events, namely mutually exclusive exons (MXE), alternative 3′ splicing site (A3SS), alternative 5′ splicing site (A5SS), retained intron (RI) and skipped exon (SE) were investigated (Fig. 9). As an example, the number of statistically significant alternatively spliced RNA isoforms detected in each genotype is illustrated for shoot tissue in the facets of the stacked bar chart (Fig. 10a, b). *Retained intron* was the most common alternative splicing event (34.2–79.5%) and *mutually exclusive exons* were least detected (0–7.3%) in all scenarios. The mature anthers and pistil—when anthers are green and immature, showed the least alternative spliced events in comparison with other tissues. The number of alternatively spliced events detected in the other nine tissues are presented in Supplementary Figs. 20–28. Among the multiple alternatively spliced events detected, 69%-78% corresponded to homoeologous genes in triads (Fig. 10c and Supplementary Figs. 20–28). In all comparisons, more alternative splicing events were associated with D homoeologues, except in the shoot tissue of C45 vs parents, where an equal proportion of events was associated with all three subgenomes (Fig. 10c and Supplementary Figs. 20–28). Overall, large-scale quantitative repression of the D subgenome was observed in SHW lines as measured by homoeologous expression biases and, qualitative changes were also more prominent in this subgenome as illustrated by the larger representation of alternative RNA splicing events.

## Discussion

The process of introgressing desirable novel alleles and haplotypes underlying phenotypes from genetically diverse parents known as pre-breeding is a viable strategy to develop cultivars with improved disease resistance, quality and agronomic traits, including climate resilience traits. However, hybridizations among diverse parents often result in poor penetrance and expressivity of traits sourced from donor parents. SHWs are being generated to access the genetic diversity present in the *Ae. tauschii* genepool (D genome donor) to overcome this allelic diversity bottleneck of bread wheat, wherein poor recovery of parental traits were reported when their genomic background changes from diploid (DD) to hexaploid (AABBDD)^19^. Hence, a critical understanding of the genome-wide impact of polyploidization in crop species is needed to design strategies to enhance the potential of pre-breeding. In this study, we investigated and unraveled the global expression level alterations in SHW lines and their corresponding tetraploid and diploid progenitors to portray the transcriptome dynamics taking place during initial polyploidization events. The four parents used in the study were diverse in geographical origin. Specifically, *T. turgidum* cv. Langdon is from North America (Langdon) and PI377655 is from former Yugoslavia, while *Ae. tauschii* AS2386 and AS2399 are from Iran^37,38^. The primary SHW plants used in the experiment had been selfed for at least four generations; these selfings potentially facilitated the stabilization of the genome compared to nascent polyploids^39^, allowing the capture of heritable transcriptomic variations.

The triad gene sets (genes present in 1:1:1 condition in ABD homeologue chromosomes) form the vital portion of the wheat genome playing key functional roles, while the genes present as other homoeologue copy number variations perform other functions^40^. In all ten developmentally distinct tissues in the four SHW lines, the majority of the genes from the D subgenome were suppressed in SHWs, when compared with their parental levels of expression. Li et al.^41^ in their transcriptome analysis of resynthesized wheat, have also shown the shift in expression dominance while comparing parental and hexaploid states. Changes in expression bias among homoeologues upon whole genome duplication have been observed in other allopolyploid crops upon resynthesis, including in rapeseed^42^ and cotton^43^, reflecting the subgenome dominance in these species as well. However, in bread wheat, it was proposed that a balance is prevalent among the subgenomes, without any subgenome dominance^34,40^. Nevertheless, tissue-, growth stage-, and environmental cue- dependent dominance have been reported as the cause of developmental process and environmental adaptations^44,45^. In this study, based on the observations in the SHW lines, the expression level dominance in the parental states is reduced by polyploidization, navigating the evolutionary process towards achieving an overall balanced allohexaploid, while the homoeologue-specific variability is capitalized by the developmental or environmental requirements. In domesticated wheat genotypes, the expression of D homoeologues were found to be less repressed than the A and B homoeologues^36,40^. Hence, it can be considered that the initial broad-scale repressive effect on D homoeologues is to establish a balance in expression between genomes derived from different parents (AB and D), and not for the subjugation of the D subgenome. Further, the expression bias patterns established in newly synthesized polyploids are maintained over multiple generations through the processes of domestication and breeding, as observed in the RNA-seq data from more stabilized genomes such as Chinese Spring (a landrace) and Azhurnaya (a cultivar), sourced from the open-source databases corresponding to Ramírez-Gonzalez et al.^36^. Hence, the initial novel expression patterns (repression of D subgenome) observed in newly synthesized hexaploid wheat, are stabilized and inherited over multiple generations.

Polyploidization events involve genome reshuffling and can alter the gene dosage either immediately after duplication, or over a long period of time (million year scale) during evolution^46^. Here we showed the instantaneous effects (within five generations of selfing since initial crossing) of polyploidization in hexaploid wheat. The shift in expression balance can be attributed to gene dosage (allele copies) because biologically-significant stoichiometry of macromolecule complexes and regulatory genes impacting downstream loci are critical for the fitness of an organism^47^. The difference among expression bias of the same gene sets between tissues may be attributed to the expression contribution of the duplicates that might differ in a tissue-specific pattern, especially in the reproductive cells^48,49^. Notably, five of the ten tissues used in the study possibly had a higher fraction of haploid gametophytic cells, referred to as the reproductive pool. In yet another dimension, polyploidization events increase the complexity of the gene regulatory network, through the introduction of inter-genomic trans-regulatory mechanisms^50^. This could result in homoeoallele-specific expression as observed in previous studies in wheat^45^ (2*n* = 6*x* = 42), high-ploidy sugarcane^51^ (2*n* = 8x–12x = 80–120) and peanut^52^ (2*n* = 4*x* = 40). Further, the individual developmental state of the plant and the genotype may also have an influence over allele specificity^51,53^, further explaining the homoeoallele expression variation across the ten developmentally-distinct tissues and four genetically different SHW lines used in our investigation.

Gene expression dynamics is the translational component linking observed phenotypic variation and its underlying genotype. Several triads showing similar expression bias patterns in the SHWs, Chinese Spring and Azhurnaya, indicate this subset remains unaffected by artificial selection. The other triads having altering HEB patterns in the resynthesized, domesticated and elite wheat backgrounds, imply the selection for specific expression bias patterns during domestication and crop improvement, which are potentially coupled to other genes regulating the characteristics of modern-day bread wheat. The limitation of this comparison is that the SHW lines used in the study are not the direct progenitors of neither Chinese Spring or Azhurnaya and the expression data are from different studies. Hence, further interrogation with multiple wheat landraces and elite wheat cultivars will shed more light on the selection for HEB.

While investigating the bias trends of triads across the tissues, the number of triads showing unbiased expression pattern across all tissues were the largest, aligning with the observation that major subsets of triads did not show significant bias in all four SHW lines in all ten tissues. The HEB specific to biotic^45^ and abiotic^54^ stress response have been reported in wheat and allopolyploid cotton. The results from this study, showing preponderance of triad-specific bias trends across developmental stages, suggest the spatial and temporal variability of HEB, leading to different bias trends through developmental stages, in addition to stress responsive patterns. In allopolyploid cotton, a study analysing the fiber development, revealed the changes in chromatin architecture in the different developmental stages and their influence on gene regulatory networks (GRNs) leading to HEB^55^. This highlights the potential role of dynamic variation in 3D chromatin architecture in bringing about spatiotemporal differences in HEB. In addition, this also conveys how the expression plasticity in allopolyploids are leveraged throughout development.

Beyond the effect of the environmental stressors on expression bias, variability in expression bias in the different tissues has also been reported in several alloplyploid species, such as rapeseed^56^, cotton^57^, coffee^58^ and white clover^59^. Previous work in hexaploid wheat has also shown that triads with varied expression bias patterns across tissues are more tissue specific^36^. In this study using SHW transcriptomes, the moderate association between homoeologue expression bias and tissue-specificity of homoeologues in the triads, elucidate the potential role of expression specificity of homoeologues in driving the direction of the expression biases. Hence, the tissue, developmental stage, environmental cues and parental genotype, all determine the bias patterns of the triads through exploiting the additional plasticity in the hexaploid wheat genomes when compared to their tetraploid and diploid progenitors.

In addition to comparison of expression patterns among homoeoalleles, the differential expression analysis at the whole genome level between the parents and SHW lines performed to characterize the expression changes of the genes between different genomic backgrounds, revealed the large-scale repression of the D subgenome genes. The introgression of chromosomal segments from species in the secondary and tertiary gene pools has resulted in transcriptomic changes in both wheat^60–63^ and other polyploid species^64,65^. Specifically, transcriptomic analysis of wheat × *Ambylopyrum muticum* introgression lines indicated the suppressed expression of genes in the introgressed segments^60^. Similarly, a higher proportion of genes in the introgressed region were down regulated in the expression studies of wheat-barley addition lines^61^. Introgression of alien chromatin segments into a species leads to the suppression of alien transcripts by the host genome; though how the host genome distinguishes the alien genome remains unclear. Combining the D genome from *Ae. tauschii* with the AB genome in *T. turgidum* results in analogous responses, hinting that the AB genome acts as the native genome and the D genome as the introduced foreign chromatin in SHWs.

The outcome of a polyploidization event at the genomic, and consequently at the phenotypic level is dependant on the genetic background of the lines involved in the hybridization event^66,67^. The assessment of multiple SHW lines derived from diverse tetraploid and diploid accessions showed variability in response to FHB infection, which could not be envisaged based on the FHB responses of the parent^68^. Similar variability based on genotypic background effects has been observed for yield-related traits of wheat lines with rye introgressions^69^.

The expression level differences from polyploidization to achieve dosage-balance, to establish homoeollele-specific expression or in exhibiting genomic background effects, are all potentially the manifestation of the genetic and epigenetic changes that occur upon polyploidization. The chromosome-level analysis of synthetic lines and their tetraploid and diploid parents using molecular cytogenetic techniques have depicted structural differences in chromatin regions^70^, and genome sequence level analysis have also revealed similar outcomes^71,72^. In an epigenetic perspective, microRNAs (miRNAs), the key players in post transcriptional gene regulation, were expressed in a non-additive manner in resynthesized wheat^41^. The modified expression of miRNAs possibly underlies the downstream shift in expression bias and repression in the mRNA data. Small interfering RNAs (siRNAs) derived from double stranded RNA molecules were involved in the methylation of DNA sequences, specifically transposable elements, and also in establishing repressive heterochromatin marks^73^. Comparison of a newly synthesized hexaploid wheat and its *Ae. tauschii* parent revealed increased accumulation of siRNAs on the D homoeologues in the hexaploid, in contrast to the observations in *T. turgidum* vs hexaploid A subgenomes^41^. The increased accumulation of siRNAs could be the underlying cause for the repression of D homeologues in hexaploids^41^. DNA methylation is an epigenetic feature for gene expression regulation and genome stability through silencing of transposable elements (TEs). The ploidy-level changes drive modification of methylation patterns in CG, CHG and CHH sequence contexts in wheat, involving the TEs as well. As TEs occupy the major proportion of the wheat genome, such epigenetic reprogramming of the TE fraction modulates expression of genes in its vicinity because TEs harbor promoter and enhancer motifs^74,75^. Among the multiple histone marks in the genome, an increase of the repressive histone-3 lysine-27 dimethylations (H3K27me2) was found to be positively correlated with the increase in ploidy in wheat^76^. Furthermore, the H3K27me3 marks underlie the subgenome-specific activity of regulatory elements^77^, and the introduction of the D subgenome triggers distinct histone modifications and consequent inter-subgenome regulatory interactions^29^. The chromatin accessibility, which is controlled by association between transcription factors and nucleosomes, is lower in the D subgenome of hexaploids compared to the *Ae. tauschii* background, also leading to extensive reduction in gene expression^78^. In addition to these genetic and epigenetic changes, alteration of chromatin architecture upon polyploidization also warrants investigation in hexaploid wheat. For instance, changes in topologically-associated domains (TAD) and A/B compartmentalization have been reported in other polyploid crop species like watermelon^79^ and cotton^80^.

Following gene transcription, precursor mRNAs are modified using the splicing machinery to produce mature RNAs. During this process, the splicing machinery can retain different combinations of introns and exons by alternative splicing (AS). This AS mechanism can diversify the transcriptional and translational products of a gene, thereby altering its function in the developmental frameworks of space (different tissues) and time (vegetative vs reproductive stages)^81^. There are tissue-specific isoforms, and abiotic^82^ and biotic^83^ stress-induced AS events. However, the impact of polyploidization and the resulting genomic shock on AS have only recently been investigated. In a study of hexaploid wheat and its tetraploid and diploid parents and relatives, Yu et al.^84^ also found that retained intron was the most common AS mechanism observed. The dominance of intron retention has been observed across plant species, including Arabidopsis^85^, rice, sorghum and maize^86^ and cotton^87^. The influence of polyploidization on differential splicing events has been observed in allopolyploid rapeseed, along with homoeolog-specific differences in AS^88^. The characterization of AS in the transcriptome derived from embryogenesis tissues in wheat and its progenitors, had a preponderance of alternative 3’ splicing events, however, the difference has been attributed to AS-event specificity of the analysis tool and percent spliced in thresholds utilized^89^.

In conclusion, the global transcriptome dynamics analysis in SHWs compared to its tetraploid and diploid parents revealed expression bias among homoeologues resulting in large-scale suppression of the D subgenome in the SHWs as inferred from more than 18,000 triads and expression from ten tissues. Qualitative changes observed among transcripts in the form of novel splice variants upon polyploidization also majorly impacted the D genome homoeologues. These quantitative (transcript abundance) and qualitative (splice variants) expression changes seem to be dependent on the genomic composition of the parents and the developmental stage (tissues) of the wheat lines. To harness the genetic diversity available in the D genome progenitors (*Ae. tauschii*) and other wild relatives for wheat improvement through interspecific hybridization, the potentiality of modified expression in the progeny must be taken into consideration to recover the desired phenotype in the polyploid background.

## Methods

### Plant materials

Four SHW lines (C44, C45, C65 and C66) and their two tetraploid (PI377655–C16, Langdon–C19) and two diploid (AS2386–C26, AS2399–C30) parents, for a total of eight genotypes, were used in the study (Supplementary Table 1). The following ten tissue samples (Supplementary Fig. 1) were collected from the above mentioned eight lines: (1) head at boot stage, (2) shoot collected at heading, (3) lemma and palea at heading, (4) glume from first and second floret of a spike, (5) pistil—when anthers are green and immature, (6) pistil—when anthers are yellow and just prior to dehiscence, (7) pistil—one day after anthesis, (8) yellow anther just prior to dehiscence, (9) hypocotyl and (10) root, where the latter two tissues were collected from young seedlings grown on Whatman filter papers. Three biological replicates per tissue from all the eight genotypes were used in the study (ten tissues × eight genotypes × three replicates = 240 samples).

### RNA extraction and library preparation

The RNeasy Plant Mini Kit (Qiagen Inc., Germantown, MD, USA) was used for all tissues except for the mature anther and pistil when anthers are green and immature, which were isolated using miRNeasy mini kit (Qiagen Inc). The extracted RNAs were quantified initially with the Implen Nanophotometer (Implen Inc, Westlake Village, CA, USA) and the quality and concentration were assessed with the Agilent RNA 6000 Nano assay (Agilent Technologies, Santa Clara, CA, USA). The construction of cDNA libraries and sequencing were carried out at the Centre d’expertise et de services Génome Québec (Montreal, QC, Canada). Briefly, mRNA enrichment was performed using NEBNext Poly(A) Magnetic Isolation Module (New England BioLabs, Ipswich, MA, USA), followed by cDNA synthesis and library preparation with the NEBNext RNA First Strand Synthesis, NEBNext Ultra Directional RNA Second Strand Synthesis modules and the NEBNext Ultra II DNA Library Prep Kit (New England BioLabs). Libraries were quantified using PicoGreen dsDNA assay kit, and quality was analysed using LabChip GX (PerkinElmer, MA, USA). The normalized libraries were clustered on an Illumina cBot, and 100-bp paired-end reads were generated on a HiSeq 4000 platform (Illumina Inc., CA, USA) for the mature anthers and the pistils collected when the anthers were still immature, i.e., prior to pollination. The other eight tissues were sequenced as 150-bp paired-end reads on the NovaSeq 6000 platform (Illumina Inc.).

### Processing and alignment of sequencing reads

The sequencing reads were pre-processed using Trimmomatic^90^ and aligned to the recently updated version of the bread wheat genome pseudomolecules (RefSeq v2.1; Annotation v2.1)^33^ using Spliced Transcripts Alignment to a Reference (STAR)^91^, after genome indexing, with the maximum intron length set to 10,000 bp, the maximum number of allowed mismatches as six, and the other parameters left at their default settings.

### Estimation of homoeologue expression bias (HEB) and likelihood ratio test (LRT)

In the bread wheat genome, nearly 35% of the genes are present as triads, with one homoeologous copy in each of the subgenomes (1:1:1 in A:B:D)^34^. A total of 18,474 triads were identified using the Refseq v1.0 annotation. However, after taking into account the revisions made in Refseq v2.1 annotation, a set of 18,357 triads, comprised of gene models annotated with high confidence^33,34^, were used for this study of comparative transcriptomes. An expression matrix was generated from the gene count data, and the reads per kilobase per million mapped reads (RPKM) values were estimated from the raw count data using the *rpkm* function in edgeR^92^. Homoeologue expression bias (HEB), representing the number of fold bias and its direction, was estimated using the following formula^35^.

$${{{{{\rm{HEB}}}}}} = {\log }_{2}\left({\frac{{{{{{\rm{RPKM}}}}}}_{{{{{\rm{D}}}}}}}{{{{{{\rm{RPKM}}}}}}_{{{{{\rm{AB}}}}}}}}\right)$$

Where, RPKM_AB_ and RPKM_D_ represent the expression values from the AB and D subgenomes, respectively. For the RPKM expression values from the AB subgenome, the average between the expression levels of the A and B homoeologues of a triad gene set was utilized, considering the balanced expression levels of the A, B and D homoeologues, and the difference in gene length is accounted for by the normalization.

Further, the likelihood ratio test developed on MATLAB by Smith et al.^35^ and first used in Edger et al.^93^, was applied to identify the triads showing significant bias towards the tetraploid (AABB) or diploid (DD) genomes. Likelihood ratio tests were performed using the expression data from the SHW lines as well as the data from their corresponding tetraploid and diploid parents, creating an *in-silico* SHW without inter-subgenome interactions, in order to determine the shift in expression bias upon polyploidization. The upstream input preparation and downstream summarization steps were performed with custom scripts in bash and R. In order to investigate the bias patterns of tissue-specific and broadly expressed genes, the Tau method^94^ was used for all the genotypes with a cut-off of 0.8. The strength of association between the tissue specificity of the homoeologues and the expression bias of the triads was estimated using Cramer’s-V statistic and the significance of association was determined using the Chi-square test of independence. The gene ontology analysis of the homoeologues in triads of root and mature anther tissues with A, B and D homoeologues showing same tissue specificity across all four SHW lines, was performed using the Triticeae-Gene Tribe tool kit^95^. The R scripts used for input file preparation for LRT and tissue-specificity analyses are available at https://github.com/akshaya-v/SHW-Expression-bias.

To unravel the HEB in domesticated wheat lines, the RNA-seq data from Ramírez-Gonzalez et al.^36^ for the landrace Chinese Spring and the cultivar Azhurnaya were downloaded for the following tissues: Chines Spring: leaf (at 14 days), root (at 14 days), ovary (early anthesis), ovary (late anthesis) and spike (at booting); Azhurnaya: stigma and ovary (anthesis), glumes (milk grain stage), anther (anthesis), root (seedling), spike (30% spike out). The downstream read processing, alignment and HEB analysis was carried out as described above for the SHWs.

### Differential expression analysis

The read count data from the ten tissues were grouped into two pools: vegetative (shoot, root, hypocotyl, glume and palea+lemma) and reproductive (head, pistil—when anthers are green, pistil—when anthers are yellow, pistil—one day after anthesis and yellow anther just prior to dehiscence). The logic was to group the tissues comprised of only vegetative cells into one pool and the tissues comprised of both vegetative and reproductive cells into another pool. The differential expression analysis in the two pools was performed using the edgeR statistical package^92^, and a false discovery rate (FDR) of <0.05 and log fold change (LogFC) cut-off of ±2.00 were applied as thresholds for inferring differentially expressed genes.

### Alternative splicing analysis

The alternative splicing analysis was carried out using rMATS^96^. The BAM files generated using STAR were utilized. The allow-clipping option was enabled to accept alignments with soft clipping. The detection of novel splice sites not present in the annotation files were allowed using the novelSS option. The alternative splicing analysis was performed for all eight possible SHW vs diploid/tetraploid parent comparisons in the ten tissues. The sashimi plots were made using rmats2sashimiplot^97^. The number and types of differentially spliced isoforms detected in parents or SHW lines were summarized for comparison. The alternative splice types were alternative 3’ splice site, alternative 5’ splice site, mutually exclusive exons, retained intron and skipped exon.

### Statistics and reproducibility

Three biological replicates per tissue per genotype were utilized. There were ten tissues and eight genotypes, and hence, there were 240 tissue samples in total. The likelihood ratio test to detect significant HEB was performed with the null hypothesis that the homoeologues show equal levels of expression in AB and D subgenomes, and the alternate hypothesis that the homoeologues show unequal expression levels between subgenomes, as described in Smith et al.^35^. The differential expression analysis was performed using edgeR^92^ v3.38.4 with FDR < 0.05 and logFC cut-off ±2.00. The alternative splicing analysis was carried out with rMATS^96^ using the default splicing difference cut-off of 0.01%.

### Reporting summary

Further information on research design is available in the Nature Portfolio Reporting Summary linked to this article.

## Supplementary information

Supplementary Information

Reporting Summary

## Data Availability

The raw RNA-seq data has been deposited in the Short Read Archive (SRA) at NCBI under Bioproject PRJNA905376. The base data for the HEB figures, alternative splicing figures and tissue-specificity analyses results are available in Figshare (10.6084/m9.figshare.c.6443621) (ref. ^98^).

## Code availability

The source code of the R scripts used for preparing input files and all other analyses are provided within the Methods section.
