# Supplementary material for: Global transcriptome analysis of allopolyploidization reveals large-scale repression of the D-subgenome in synthetic hexaploid wheat
Source: Commun Biol. 2023 Apr 17;6:426. doi: 10.1038/s42003-023-04781-7 (PMC10110605; doi:10.1038/s42003-023-04781-7)
Supplement: Supplementary file 1 — Supplementary Information [file 42003_2023_4781_MOESM1_ESM.pdf]

Supplementary Information

Supplementary Table 1. Synthetic hexaploid lines and their corresponding diploid and tetraploid parents used in the study

		Male parent	
		AS2386 (DD) - C26<br>*Ae*. *tauschii*	AS2399 (DD) - C30<br>*Ae*. *tauschii*
**Female parent**	PI377655 (AABB)<br>*T. turgidum* ssp. *dicoccum*	C66 (AABBDD)	C65 (AABBDD)
	Langdon (AABB)<br>*T. turgidum* ssp. *durum*	C44 (AABBDD)	C45 (AABBDD)

Supplementary Table 2. Number of raw reads in the 240 RNA-seq samples

		Diploid - AS2386	Diploid - AS2399	SHW - C44	SHW - C45	SHW - C65	SHW - C66	Tetraploid - Langdon	Tetraploid - PI377655
**Pistil-1DAA**	**Rep1**	22,195,307	21,995,116	33,587,073	26,493,898	38,641,474	26,548,645	28,846,234	28,035,524
	**Rep2**	22,995,789	34,757,108	36,490,086	33,720,903	39,157,764	23,082,570	44,131,554	22,652,205
	**Rep3**	24,015,698	22,989,427	91,812,896	23,633,788	27,469,412	31,711,344	26,122,325	22,785,314
**Boot**	**Rep1**	76,018,937	32,301,846	28,027,166	38,784,598	18,707,397	38,612,808	33,243,732	24,941,487
	**Rep2**	26,200,871	73,864,787	21,492,555	26,939,056	61,995,968	20,344,851	45,296,952	25,427,653
	**Rep3**	29,151,714	68,049,299	28,500,457	85,145,293	69,852,158	22,442,149	27,178,086	23,280,574
**Glume**	**Rep1**	35,420,234	29,310,601	20,759,066	28,314,129	47,565,899	28,268,183	38,976,192	64,828,969
	**Rep2**	21,937,111	23,713,143	22,781,410	47,640,557	20,144,197	44,869,236	27,482,753	22,573,351
	**Rep3**	29,713,083	21,744,437	23,737,425	26,490,383	21,575,767	31,696,463	40,205,086	23,904,763
**Hypocotyl**	**Rep1**	30,449,989	20,003,076	13,082,000	48,991,348	18,179,165	18,007,903	28,016,934	38,735,285
	**Rep2**	22,841,340	31,214,263	23,833,152	51,412,571	27,737,105	29,903,001	42,518,094	27,349,705
	**Rep3**	63,882,306	46,891,825	50,655,072	28,343,485	51,303,621	22,989,560	39,910,639	40,895,942
**Palea+Lemma**	**Rep1**	24,328,003	32,518,917	24,198,386	42,344,790	20,249,452	23,162,717	64,847,457	33,799,385
	**Rep2**	26,014,180	21,748,599	22,482,334	30,774,636	25,410,801	24,318,988	25,346,626	20,559,596
	**Rep3**	27,805,863	21,900,549	21,547,690	41,084,241	29,369,761	23,520,086	22,848,870	40,133,310
**Pistil-AM**	**Rep1**	26,023,695	28,445,422	56,573,952	27,251,070	24,266,368	24,290,980	53,144,692	24,637,191
	**Rep2**	23,690,579	20,529,663	12,878,054	23,573,721	26,393,617	24,437,509	30,271,276	23,713,978
	**Rep3**	20,806,267	27,419,849	25,902,215	24,955,332	27,162,721	23,635,128	38,392,332	32,237,698
**Root**	**Rep1**	37,598,774	28,541,828	33,567,318	26,045,053	25,478,138	19,511,358	34,691,430	26,340,471
	**Rep2**	29,137,833	25,129,915	23,934,237	35,683,705	29,225,962	21,364,532	21,883,804	24,330,645
	**Rep3**	47,832,348	25,090,283	22,864,680	30,691,902	21,624,952	26,808,534	25,119,702	28,174,617
**Shoot**	**Rep1**	27,411,093	23,796,164	30,077,914	25,784,399	27,149,920	35,503,678	42,150,240	23,856,393
	**Rep2**	29,526,120	23,787,924	23,346,192	25,126,541	40,080,997	19,545,212	54,426,481	25,778,462
	**Rep3**	27,553,375	12,778,098	53,706,831	28,990,179	48,394,047	34,000,995	36,586,301	24,547,842
**Anther**	**Rep1**	14,858,969	12,068,339	13,332,346	12,464,913	15,245,079	14,566,281	14,138,343	12,871,028
	**Rep2**	13,950,637	14,561,991	12,961,043	13,591,105	11,329,218	14,192,473	14,466,506	12,874,863
	**Rep3**	13,939,729	13,903,778	12,948,472	14,120,579	13,776,126	13,739,453	12,189,781	14,738,894
**Pistil-AI**	**Rep1**	13,188,605	13,449,612	13,036,625	12,154,908	13,265,009	13,370,423	13,956,148	13,240,572
	**Rep2**	12,789,103	13,009,865	12,653,047	12,378,022	12,666,519	13,168,331	13,237,777	15,109,353
	**Rep3**	11,175,795	14,047,307	13,584,399	13,336,772	12,082,013	6,573,063	13,008,756	11,463,936

Pistil-1DAA: pistil-one day after anthesis; Pistil-AM: pistil-when anthers are at mature stage; Pistil-AI: pistil-when anthers are at immature stage; Boot: head at boot stage

Supplementary Table 3. Number of reads in the 240 RNA-seq samples, after processing

		Diploid - AS2386	Diploid - AS2399	SHW - C44	SHW - C45	SHW - C65	SHW - C66	Tetraploid - Langdon	Tetraploid - PI377655
**Pistil-1DAA**	**Rep1**	16,652,462	15,620,498	23,790,476	18,114,304	26,722,162	18,910,673	21,160,261	20,875,899
	**Rep2**	15,274,606	24,308,623	27,552,871	22,883,456	26,607,670	16,101,400	32,282,843	16,014,445
	**Rep3**	17,596,827	16,943,904	70,743,378	16,474,019	19,708,725	21,376,446	18,355,411	15,236,838
**Boot**	**Rep1**	57,664,218	24,949,933	21,275,022	29,714,612	13,089,654	30,169,811	23,571,404	17,661,315
	**Rep2**	19,408,624	57,391,546	15,926,482	20,617,391	47,873,378	14,793,083	34,287,497	18,371,225
	**Rep3**	20,664,589	53,646,495	20,195,131	64,951,442	49,579,406	15,987,495	19,319,412	17,171,614
**Glume**	**Rep1**	26,815,121	20,940,544	15,246,336	19,648,878	32,319,338	21,431,871	29,463,422	48,193,103
	**Rep2**	16,181,955	17,665,911	17,013,063	34,431,917	14,395,762	34,300,068	19,643,543	16,772,106
	**Rep3**	23,384,600	15,494,944	16,957,425	18,430,477	15,674,851	22,514,609	28,535,092	18,511,746
**Hypocotyl**	**Rep1**	22,561,521	14,100,352	10,041,404	37,561,278	13,827,431	13,993,188	19,525,603	27,837,920
	**Rep2**	16,526,306	22,602,975	17,221,309	40,381,040	20,276,202	21,450,701	30,098,228	20,226,843
	**Rep3**	48,387,770	34,862,966	36,242,751	21,403,064	35,854,302	16,338,510	28,766,799	29,606,341
**Palea+Lemma**	**Rep1**	18,391,019	25,039,738	17,712,620	29,284,744	14,637,765	17,059,434	48,637,322	24,787,679
	**Rep2**	20,019,127	15,967,411	16,328,130	21,710,695	18,812,481	17,763,568	17,905,772	14,222,274
	**Rep3**	19,870,649	16,053,756	16,023,215	32,167,434	21,649,321	17,532,475	17,107,285	28,181,077
**Pistil-AM**	**Rep1**	18,349,055	20,387,882	39,204,701	19,597,028	17,808,171	18,540,363	39,515,015	17,642,079
	**Rep2**	17,009,153	14,338,346	9,774,662	17,003,537	18,099,276	16,707,667	20,147,366	16,541,649
	**Rep3**	14,373,624	19,829,020	19,493,466	18,377,313	19,459,379	17,142,187	28,539,125	23,726,105
**Root**	**Rep1**	25,851,418	21,188,494	24,812,414	18,371,284	19,203,581	13,541,267	26,718,856	19,765,758
	**Rep2**	21,390,326	18,620,533	17,297,014	26,998,387	22,692,101	15,503,742	15,431,234	17,525,916
	**Rep3**	33,999,451	17,916,134	15,909,748	21,996,230	15,535,210	19,776,405	19,374,245	19,934,284
**Shoot**	**Rep1**	19,523,100	18,255,513	22,491,360	18,934,613	19,607,143	27,386,269	30,091,216	16,814,046
	**Rep2**	22,614,844	17,088,763	17,308,713	17,897,686	28,082,616	14,874,879	38,629,216	18,534,622
	**Rep3**	21,063,835	10,026,717	38,010,200	21,020,090	34,583,788	24,084,686	27,829,956	17,347,074
**Anther**	**Rep1**	10,862,366	8,457,261	10,832,242	9,653,328	12,421,104	10,966,326	10,320,422	10,231,104
	**Rep2**	11,112,928	11,739,559	9,546,590	10,147,261	9,136,176	11,468,430	11,543,432	10,230,761
	**Rep3**	10,378,299	9,800,090	9,542,973	11,388,733	9,912,320	9,921,095	9,810,303	11,785,351
**Pistil-AI**	**Rep1**	9,854,619	11,066,191	10,647,010	9,158,324	10,770,855	9,972,559	10,392,686	10,512,209
	**Rep2**	9,304,179	9,562,473	9,243,986	10,093,944	10,334,726	9,781,472	10,698,688	11,338,157
	**Rep3**	8,967,274	10,234,854	11,120,788	9,712,338	9,793,567	4,829,128	9,814,128	9,472,780

Pistil-1DAA: pistil-one day after anthesis; Pistil-AM: pistil-when anthers are at mature stage; Pistil-AI: pistil-when anthers are at immature stage; Boot: head at boot stage

Supplementary Table 4. Summary of proportion of uniquely mapped and multi-mapped reads

Genotype	Source material	Tissue	Rep	Uniquely mapped reads (%)	Multi-mapped (%)	Too many multi-mapped* (%)	Total multi-mapped$ (%)
AS2386	Diploid parent	Anther	1	83.07	13.69	1.22	14.91
AS2386	Diploid parent	Anther	2	84.01	13.21	1.19	14.40
AS2386	Diploid parent	Anther	3	82.94	13.49	1.32	14.81
AS2386	Diploid parent	Glume	1	84.42	12.72	0.53	13.25
AS2386	Diploid parent	Glume	3	83.45	13.74	0.50	14.24
AS2386	Diploid parent	Glume	2	85.43	11.07	1.15	12.22
AS2386	Diploid parent	Boot	3	88.18	8.95	0.36	9.31
AS2386	Diploid parent	Boot	2	87.34	9.74	0.75	10.49
AS2386	Diploid parent	Boot	1	88.09	9.32	0.38	9.70
AS2386	Diploid parent	Hypocotyl	1	85.23	11.11	1.21	12.32
AS2386	Diploid parent	Hypocotyl	3	87.49	9.46	0.58	10.04
AS2386	Diploid parent	Hypocotyl	2	85.63	10.90	0.95	11.85
AS2386	Diploid parent	Palea+Lemma	3	85.55	10.33	0.98	11.31
AS2386	Diploid parent	Palea+Lemma	1	86.50	10.11	0.90	11.01
AS2386	Diploid parent	Palea+Lemma	2	86.34	10.64	0.67	11.31
AS2386	Diploid parent	Pistil-1DAA	3	87.59	7.47	2.24	9.71
AS2386	Diploid parent	Pistil-1DAA	2	85.98	7.95	3.05	11.00
AS2386	Diploid parent	Pistil-1DAA	1	89.04	7.23	1.33	8.56
AS2386	Diploid parent	Pistil-AM	3	87.70	7.84	1.83	9.67
AS2386	Diploid parent	Pistil-AM	1	86.76	7.94	2.51	10.45
AS2386	Diploid parent	Pistil-AM	2	87.66	7.85	1.66	9.51
AS2386	Diploid parent	Pistil-AI	3	83.79	13.42	0.54	13.96
AS2386	Diploid parent	Pistil-AI	2	83.12	13.82	0.61	14.43
AS2386	Diploid parent	Pistil-AI	1	82.82	13.86	0.61	14.47
AS2386	Diploid parent	Root	5	88.36	6.68	2.19	8.87
AS2386	Diploid parent	Root	4	88.78	6.64	1.99	8.63
AS2386	Diploid parent	Root	2	89.52	6.83	0.93	7.76
AS2386	Diploid parent	Shoot	2	89.12	8.15	0.31	8.46
AS2386	Diploid parent	Shoot	1	86.94	9.44	0.88	10.32
AS2386	Diploid parent	Shoot	3	86.90	10.25	0.51	10.76
AS2399	Diploid parent	Anther	4	83.23	14.42	0.57	14.99
AS2399	Diploid parent	Anther	2	81.54	14.89	0.93	15.82
AS2399	Diploid parent	Anther	3	80.38	13.59	1.65	15.24
AS2399	Diploid parent	Glume	3	83.82	13.01	0.60	13.61
AS2399	Diploid parent	Glume	2	85.70	10.47	1.05	11.52
AS2399	Diploid parent	Glume	1	83.42	13.20	0.74	13.94
AS2399	Diploid parent	Boot	3	88.17	9.14	0.39	9.53
AS2399	Diploid parent	Boot	1	87.08	9.79	0.93	10.72
AS2399	Diploid parent	Boot	2	86.15	11.28	0.34	11.62
AS2399	Diploid parent	Hypocotyl	1	83.53	12.81	1.17	13.98
AS2399	Diploid parent	Hypocotyl	3	85.57	11.38	0.67	12.05
AS2399	Diploid parent	Hypocotyl	2	83.78	12.60	0.97	13.57
AS2399	Diploid parent	Palea+Lemma	1	85.94	11.05	0.37	11.42

AS2399	Diploid parent	Palea+Lemma	2	86.17	10.13	0.81	10.94
AS2399	Diploid parent	Palea+Lemma	3	86.84	9.74	0.71	10.45
AS2399	Diploid parent	Pistil-1DAA	1	86.10	7.47	3.64	11.11
AS2399	Diploid parent	Pistil-1DAA	3	88.03	7.38	2.18	9.56
AS2399	Diploid parent	Pistil-1DAA	2	85.31	7.91	3.71	11.62
AS2399	Diploid parent	Pistil-AM	2	84.95	8.02	4.04	12.06
AS2399	Diploid parent	Pistil-AM	1	85.55	7.82	3.55	11.37
AS2399	Diploid parent	Pistil-AM	3	86.51	7.62	3.24	10.86
AS2399	Diploid parent	Pistil-AI	2	82.28	13.75	0.89	14.64
AS2399	Diploid parent	Pistil-AI	1	83.05	13.89	0.52	14.41
AS2399	Diploid parent	Pistil-AI	3	83.54	13.72	0.50	14.22
AS2399	Diploid parent	Root	1	88.70	6.39	2.29	8.68
AS2399	Diploid parent	Root	4	89.31	7.42	0.70	8.12
AS2399	Diploid parent	Root	3	88.88	6.47	2.20	8.67
AS2399	Diploid parent	Shoot	2	84.25	12.07	0.78	12.85
AS2399	Diploid parent	Shoot	1	83.29	13.83	0.56	14.39
AS2399	Diploid parent	Shoot	3	87.57	9.57	0.53	10.10
C44	SHW	Anther	1	82.15	13.41	0.83	14.24
C44	SHW	Anther	6	81.42	13.70	0.90	14.60
C44	SHW	Anther	8	82.04	13.12	1.31	14.43
C44	SHW	Glume	3	84.89	11.03	0.96	11.99
C44	SHW	Glume	2	85.48	10.13	1.10	11.23
C44	SHW	Glume	1	84.42	11.73	0.94	12.67
C44	SHW	Boot	1	88.37	8.29	0.53	8.82
C44	SHW	Boot	2	88.02	8.53	0.79	9.32
C44	SHW	Boot	3	87.33	9.08	0.67	9.75
C44	SHW	Hypocotyl	1	85.67	10.72	0.68	11.40
C44	SHW	Hypocotyl	3	85.35	10.25	1.24	11.49
C44	SHW	Hypocotyl	2	86.52	9.25	1.12	10.37
C44	SHW	Palea+Lemma	3	85.77	10.87	0.61	11.48
C44	SHW	Palea+Lemma	1	85.34	10.63	0.77	11.40
C44	SHW	Palea+Lemma	2	85.40	11.01	0.58	11.59
C44	SHW	Pistil-1DAA	2	88.49	6.82	1.83	8.65
C44	SHW	Pistil-1DAA	1	87.50	7.20	2.17	9.37
C44	SHW	Pistil-1DAA	3	89.60	6.85	0.82	7.67
C44	SHW	Pistil-AM	1	86.18	7.82	2.70	10.52
C44	SHW	Pistil-AM	2	89.61	7.02	0.61	7.63
C44	SHW	Pistil-AM	3	86.58	7.16	3.25	10.41
C44	SHW	Pistil-AI	4	83.76	13.04	0.63	13.67
C44	SHW	Pistil-AI	3	83.18	13.26	0.70	13.96
C44	SHW	Pistil-AI	2	83.64	13.00	0.63	13.63
C44	SHW	Root	1	88.52	7.28	0.66	7.94
C44	SHW	Root	3	86.41	7.23	2.84	10.07
C44	SHW	Root	2	88.69	7.08	0.96	8.04
C44	SHW	Shoot	2	86.75	9.31	0.89	10.20
C44	SHW	Shoot	3	85.68	10.24	0.89	11.13
C44	SHW	Shoot	1	84.31	12.06	1.02	13.08

C45	SHW	Anther	2	81.86	13.66	0.75	14.41
C45	SHW	Anther	5	83.46	12.90	0.61	13.51
C45	SHW	Anther	1	82.71	13.29	0.83	14.12
C45	SHW	Glume	3	78.84	17.61	0.63	18.24
C45	SHW	Glume	1	83.85	12.27	0.64	12.91
C45	SHW	Glume	2	84.55	11.29	0.88	12.17
C45	SHW	Boot	2	86.66	10.34	0.44	10.78
C45	SHW	Boot	3	87.99	9.03	0.38	9.41
C45	SHW	Boot	1	85.51	11.40	0.45	11.85
C45	SHW	Hypocotyl	1	87.90	8.51	0.59	9.10
C45	SHW	Hypocotyl	2	88.68	7.93	0.41	8.34
C45	SHW	Hypocotyl	3	86.47	10.05	0.55	10.60
C45	SHW	Palea+Lemma	3	84.51	11.99	0.82	12.81
C45	SHW	Palea+Lemma	2	84.89	10.65	0.93	11.58
C45	SHW	Palea+Lemma	1	83.48	12.61	0.72	13.33
C45	SHW	Pistil-1DAA	3	87.12	7.54	2.15	9.69
C45	SHW	Pistil-1DAA	2	84.16	7.72	4.57	12.29
C45	SHW	Pistil-1DAA	1	85.30	7.78	3.53	11.31
C45	SHW	Pistil-AM	2	83.85	7.56	5.34	12.90
C45	SHW	Pistil-AM	1	84.61	7.65	4.52	12.17
C45	SHW	Pistil-AM	3	88.15	7.36	1.66	9.02
C45	SHW	Pistil-AI	3	83.19	13.41	0.55	13.96
C45	SHW	Pistil-AI	2	83.13	13.24	0.67	13.91
C45	SHW	Pistil-AI	1	83.14	13.14	0.75	13.89
C45	SHW	Root	3	88.71	7.37	0.98	8.35
C45	SHW	Root	2	88.25	6.91	1.04	7.95
C45	SHW	Root	1	88.48	7.43	0.94	8.37
C45	SHW	Shoot	2	85.61	10.46	0.82	11.28
C45	SHW	Shoot	1	84.08	11.55	1.30	12.85
C45	SHW	Shoot	3	86.77	9.35	0.81	10.16
C65	SHW	Anther	4	84.09	13.10	0.34	13.44
C65	SHW	Anther	1	84.15	13.09	0.41	13.50
C65	SHW	Anther	3	83.91	12.89	0.46	13.35
C65	SHW	Glume	2	83.86	12.11	1.00	13.11
C65	SHW	Glume	3	82.30	13.63	1.03	14.66
C65	SHW	Glume	1	83.43	11.71	1.12	12.83
C65	SHW	Boot	1	87.09	9.56	0.42	9.98
C65	SHW	Boot	2	86.95	9.86	0.59	10.45
C65	SHW	Boot	3	85.03	11.52	0.68	12.20
C65	SHW	Hypocotyl	2	84.61	11.08	1.53	12.61
C65	SHW	Hypocotyl	1	85.58	11.20	0.65	11.85
C65	SHW	Hypocotyl	3	85.84	9.95	1.13	11.08
C65	SHW	Palea+Lemma	2	86.60	9.52	1.10	10.62
C65	SHW	Palea+Lemma	1	84.49	11.18	1.18	12.36
C65	SHW	Palea+Lemma	3	83.38	12.70	1.11	13.81
C65	SHW	Pistil-1DAA	1	85.14	7.65	3.90	11.55
C65	SHW	Pistil-1DAA	2	86.35	7.68	2.74	10.42

C65	SHW	Pistil-1DAA	3	88.30	7.43	1.53	8.96
C65	SHW	Pistil-AM	2	87.42	7.94	1.65	9.59
C65	SHW	Pistil-AM	1	84.54	7.55	4.73	12.28
C65	SHW	Pistil-AM	3	82.00	7.80	6.72	14.52
C65	SHW	Pistil-AI	3	83.41	13.12	0.58	13.70
C65	SHW	Pistil-AI	1	82.74	13.60	0.66	14.26
C65	SHW	Pistil-AI	2	82.81	13.24	0.73	13.97
C65	SHW	Root	2	90.02	6.63	0.87	7.50
C65	SHW	Root	3	87.81	7.23	2.04	9.27
C65	SHW	Root	1	89.31	6.99	1.13	8.12
C65	SHW	Shoot	1	78.86	17.40	1.12	18.52
C65	SHW	Shoot	2	82.96	12.59	1.33	13.92
C65	SHW	Shoot	3	84.72	11.39	0.88	12.27
C66	SHW	Anther	1	83.57	13.15	0.43	13.58
C66	SHW	Anther	2	83.69	13.32	0.40	13.72
C66	SHW	Anther	4	83.65	12.96	0.53	13.49
C66	SHW	Glume	3	78.74	17.81	0.70	18.51
C66	SHW	Glume	2	86.57	10.20	0.36	10.56
C66	SHW	Glume	1	85.17	11.16	0.76	11.92
C66	SHW	Boot	2	86.07	10.54	0.64	11.18
C66	SHW	Boot	1	86.61	10.58	0.37	10.95
C66	SHW	Boot	3	85.73	10.93	0.58	11.51
C66	SHW	Hypocotyl	2	83.79	12.08	1.29	13.37
C66	SHW	Hypocotyl	1	81.67	14.51	0.74	15.25
C66	SHW	Hypocotyl	3	81.80	13.30	1.86	15.16
C66	SHW	Palea+Lemma	2	84.62	11.27	1.00	12.27
C66	SHW	Palea+Lemma	3	84.10	12.39	0.82	13.21
C66	SHW	Palea+Lemma	1	83.95	12.33	0.87	13.20
C66	SHW	Pistil-1DAA	1	84.88	7.46	4.28	11.74
C66	SHW	Pistil-1DAA	2	86.41	7.71	2.82	10.53
C66	SHW	Pistil-1DAA	3	84.97	7.83	3.82	11.65
C66	SHW	Pistil-AM	3	87.19	7.54	2.42	9.96
C66	SHW	Pistil-AM	2	84.45	7.91	4.20	12.11
C66	SHW	Pistil-AM	1	87.26	7.42	2.51	9.93
C66	SHW	Pistil-AI	1	83.61	13.13	0.66	13.79
C66	SHW	Pistil-AI	3	82.76	13.70	0.62	14.32
C66	SHW	Pistil-AI	2	82.43	13.73	0.69	14.42
C66	SHW	Root	1	86.17	7.16	1.01	8.17
C66	SHW	Root	3	88.51	6.99	1.62	8.61
C66	SHW	Root	2	88.62	6.77	1.76	8.53
C66	SHW	Shoot	3	84.61	11.53	0.73	12.26
C66	SHW	Shoot	1	86.57	9.85	0.46	10.31
C66	SHW	Shoot	2	83.72	12.81	0.83	13.64
Langdon	Tetraploid parent	Glume	1	84.25	11.86	0.44	12.30
Langdon	Tetraploid parent	Glume	2	83.44	11.69	0.81	12.50
Langdon	Tetraploid parent	Glume	3	82.89	12.83	0.71	13.54
Langdon	Tetraploid parent	Boot	2	84.90	12.05	0.32	12.37

Langdon	Tetraploid parent	Boot	1	85.75	10.48	0.55	11.03
Langdon	Tetraploid parent	Boot	3	85.56	10.63	0.68	11.31
Langdon	Tetraploid parent	Hypocotyl	2	83.77	11.69	1.18	12.87
Langdon	Tetraploid parent	Hypocotyl	3	85.22	11.13	0.58	11.71
Langdon	Tetraploid parent	Hypocotyl	1	86.27	9.27	1.15	10.42
Langdon	Tetraploid parent	Palea+Lemma	1	84.15	12.03	0.48	12.51
Langdon	Tetraploid parent	Palea+Lemma	3	86.68	9.32	0.94	10.26
Langdon	Tetraploid parent	Palea+Lemma	2	84.25	11.48	0.75	12.23
Langdon	Tetraploid parent	Pistil-1DAA	2	87.76	7.15	2.01	9.16
Langdon	Tetraploid parent	Pistil-1DAA	1	87.10	7.13	2.59	9.72
Langdon	Tetraploid parent	Pistil-1DAA	3	85.99	7.57	2.76	10.33
Langdon	Tetraploid parent	Pistil-AM	2	85.70	7.61	3.21	10.82
Langdon	Tetraploid parent	Pistil-AM	1	88.66	7.02	1.21	8.23
Langdon	Tetraploid parent	Pistil-AM	3	89.36	7.08	0.58	7.66
Langdon	Tetraploid parent	Root	3	89.37	6.96	0.74	7.70
Langdon	Tetraploid parent	Root	1	89.54	7.11	0.50	7.61
Langdon	Tetraploid parent	Root	2	88.60	7.46	0.68	8.14
Langdon	Tetraploid parent	Shoot	2	80.25	15.64	0.99	16.63
Langdon	Tetraploid parent	Shoot	1	82.92	11.06	1.57	12.63
Langdon	Tetraploid parent	Shoot	3	84.79	11.67	0.53	12.20
Langdon	Tetraploid parent	Anther	5	82.93	13.31	0.48	13.79
Langdon	Tetraploid parent	Anther	2	82.21	13.42	0.56	13.98
Langdon	Tetraploid parent	Anther	3	82.88	13.22	0.48	13.70
Langdon	Tetraploid parent	Pistil-AI	1	81.58	12.61	1.28	13.89
Langdon	Tetraploid parent	Pistil-AI	2	82.75	12.36	1.07	13.43
Langdon	Tetraploid parent	Pistil-AI	4	83.08	12.35	0.96	13.31
PI377655	Tetraploid parent	Glume	3	85.34	11.69	0.32	12.01
PI377655	Tetraploid parent	Glume	1	86.14	10.32	0.49	10.81
PI377655	Tetraploid parent	Glume	2	86.42	9.48	1.11	10.59
PI377655	Tetraploid parent	Boot	1	83.87	11.71	0.45	12.16
PI377655	Tetraploid parent	Boot	3	86.20	9.42	1.59	11.01
PI377655	Tetraploid parent	Boot	2	82.08	14.53	0.53	15.06
PI377655	Tetraploid parent	Hypocotyl	3	85.68	9.90	1.09	10.99
PI377655	Tetraploid parent	Hypocotyl	1	85.28	10.56	1.20	11.76
PI377655	Tetraploid parent	Hypocotyl	2	83.98	12.25	1.03	13.28
PI377655	Tetraploid parent	Palea+Lemma	1	82.16	14.07	0.88	14.95
PI377655	Tetraploid parent	Palea+Lemma	3	83.28	13.25	0.67	13.92
PI377655	Tetraploid parent	Palea+Lemma	2	83.56	12.51	0.95	13.46
PI377655	Tetraploid parent	Pistil-1DAA	1	88.36	7.28	1.43	8.71
PI377655	Tetraploid parent	Pistil-1DAA	3	85.35	7.71	3.20	10.91
PI377655	Tetraploid parent	Pistil-1DAA	2	80.77	7.50	7.75	15.25
PI377655	Tetraploid parent	Pistil-AM	3	89.17	7.32	0.61	7.93
PI377655	Tetraploid parent	Pistil-AM	1	84.80	7.72	3.87	11.59
PI377655	Tetraploid parent	Pistil-AM	2	86.22	7.93	2.56	10.49
PI377655	Tetraploid parent	Root	4	88.54	7.42	1.01	8.43
PI377655	Tetraploid parent	Root	1	88.59	7.10	1.01	8.11
PI377655	Tetraploid parent	Root	2	88.70	7.36	0.82	8.18

PI377655	Tetraploid parent	Shoot	1	80.87	14.33	1.23	15.56
PI377655	Tetraploid parent	Shoot	2	84.28	11.30	0.95	12.25
PI377655	Tetraploid parent	Shoot	3	85.13	10.55	1.13	11.68
PI377655	Tetraploid parent	Anther	4	83.37	13.09	0.53	13.62
PI377655	Tetraploid parent	Anther	2	83.73	12.73	0.50	13.23
PI377655	Tetraploid parent	Anther	3	83.55	13.07	0.41	13.48
PI377655	Tetraploid parent	Pistil-AI	2	83.38	12.94	0.55	13.49
PI377655	Tetraploid parent	Pistil-AI	4	83.16	12.42	0.93	13.35
PI377655	Tetraploid parent	Pistil-AI	3	82.77	13.02	0.78	13.80

**Too many multi-mapped reads imply reads mapping to more than 10 loci $^{\$}$Total multi-mapped implies the sum of multi-mapped and too many multi-mapped reads*
Pistil-1DAA: pistil-one day after anthesis; Pistil-AM: pistil-when anthers are at mature stage; Pistil-AI: pistil-when anthers are at immature stage; Boot: head at boot stage.

Supplementary Table 5. Number of triads biased towards the AB and D subgenomes in the parental level of expression and the SHW background

SHW	Parents/ SHW*	Pistil-1DAA		Boot		Pistil-AM		Anther		Pistil-AI		Glume		Hypocotyl		Palea+Lemma		Root		Shoot	
		AB biased	D biased	AB biased	D biased	AB biased	D biased	AB biased	D biased	AB biased	D biased	AB biased	D biased	AB biased	D biased	AB biased	D biased	AB biased	D biased	AB biased	D biased
C44	SHW	6,886 (-2.7577)	3,556 (1.1739)	5,069 (-2.7606)	2,992 (1.2135)	3,860 (-3.7535)	1,572 (1.8123)	3,484 (-2.5037)	1,975 (1.5327)	5,243 (-2.5754)	2,477 (1.1562)	4,940 (-2.988)	2,244 (1.5222)	3,704 (-4.3743)	640 (2.6087)	5,166 (-2.9745)	2,330 (1.4397)	4,963 (-3.5897)	1,902 (1.5716)	3,167 (-4.2085)	1,376 (2.1535)
	Parents	1,471 (-1.7018)	5,626 (2.1351)	700 (-3.2717)	6,839 (1.889)	2,203 (-1.29)	3,784 (2.2471)	865 (-2.3239)	6,234 (2.4059)	1,047 (-1.7311)	1,0365 (1.5026)	2,357 (-2.1738)	7,044 (1.7993)	471 (-3.1401)	6,160 (2.554)	1,310 (-2.3041)	6,448 (2.3956)	1,053 (-2.4667)	12,714 (1.5124)	1,071 (-2.6505)	3,907 (3.6611)
C45	SHW	3,286 (-1.4999)	2,875 (1.413)	3,666 (-1.2864)	3,790 (1.233)	3,008 (-1.5487)	2,617 (1.4366)	2,064 (-1.8394)	1,730 (1.7007)	2,788 (-1.3753)	2,396 (1.3264)	815 (-3.2174)	735 (3.1225)	1,632 (-2.153)	1,723 (1.8929)	1,565 (-2.2685)	1,527 (2.1279)	2,765 (-1.5269)	2,984 (1.3928)	795 (-3.0994)	786 (2.9369)
	Parents	1,963 (-1.5472)	9,476 (1.6018)	1,369 (-1.9462)	13,604 (1.3177)	2,550 (-1.3416)	5,608 (1.7844)	1,000 (-2.1385)	5,575 (2.3361)	920 (-1.9668)	10,495 (1.4122)	2,614 (-1.8089)	5,881 (2.0119)	1,216 (-2.0073)	7,301 (2.0876)	1,985 (-1.8371)	8,586 (2.1801)	824 (-2.5347)	8,122 (2.0916)	1,221 (-2.157)	1,830 (4.6597)
C65	SHW	2,941 (-1.5634)	2,899 (1.4843)	1,957 (-1.8329)	2,163 (1.6844)	1,426 (-2.2148)	1,392 (2.1096)	1,927 (-1.9912)	1,576 (1.971)	3,343 (-1.171)	3,177 (1.1735)	3,281 (-1.599)	3,104 (1.5195)	2,308 (-1.6095)	2,591 (1.5255)	3,423 (-1.4091)	3,362 (1.3694)	3,293 (-1.3203)	3,674 (1.2486)	881 (-3.1496)	826 (3.0063)
	Parents	1,184 (-2.4306)	11,041 (1.401)	1,760 (-1.2221)	13,878 (1.3188)	1,645 (-1.941)	9,476 (1.4874)	1,092 (-2.2004)	4,402 (2.2806)	988 (-1.9739)	10,086 (1.4445)	1,853 (-2.0502)	3,764 (2.3942)	1,042 (-2.3845)	5,907 (1.864)	1,188 (-2.2121)	8,547 (2.1358)	738 (-2.5328)	9,590 (1.9212)	1,155 (-2.1834)	6,387 (2.3872)
C66	SHW	2,038 (-1.8335)	2,513 (1.6338)	2,478 (-1.4125)	3,422 (1.3084)	2,809 (-1.4906)	3,392 (1.3367)	1,841 (-2.0856)	1,740 (1.9145)	2,367 (-1.3552)	2,976 (1.2564)	1,051 (-2.8733)	1,090 (2.7251)	1,584 (-1.9159)	2,208 (1.7135)	1,348 (-2.2346)	1,633 (2.0951)	1,754 (-1.8702)	2,457 (1.6176)	793 (-3.1758)	890 (2.9798)
	Parents	1,274 (-2.0341)	11,153 (1.4293)	478 (-3.5611)	12,944 (1.3496)	1,496 (-1.7724)	9,125 (1.5867)	1,115 (-2.1407)	5,667 (2.1912)	1,099 (-1.7060)	10,128 (1.5192)	2,279 (-1.9382)	7,264 (1.7225)	512 (-3.3352)	5,597 (2.1047)	950 (-2.5973)	7,344 (2.2196)	817 (-2.7019)	13,208 (1.4689)	808 (-3.1095)	9,978 (1.991)

Pistil-1DAA: pistil-one day after anthesis; Pistil-AM: pistil-when anthers are at mature stage; Pistil-AI: pistil-when anthers are at immature stage; Boot: head at boot stage.
**Parents: Parental level of expression; SHW: Synthetic hexaploid wheat.*

The number of triads showing significant bias towards the AB and D subgenomes in the SHW and parental expression levels (in-silico SHW scenario) are summarized in this table. The values in the brackets in each cell represent the average value of the magnitude of HEB in the particular SHW-tissue scenario i.e., $\frac{1}{n}\sum_{i=1}^{n} log_2\left(\frac{RPKM_D}{RPKM_{AB}}\right)$*, where n is the number of triads showing significant bias towards the specific subgenome, and* $RPKM_D$ *and* $RPKM_{AB}$ *represent the expression values from the D and AB subgenomes, respectively.*

Supplementary Table 6. Summary of tissue specificity of homoeologues constituting the triads in the four SHW lines

	Number of triads (absolute values)				Relative percentage*			
**Homoeologue tissue specificity**	**C44**	**C45**	**C65**	**C66**	**C44**	**C45**	**C65**	**C66**
All homoeologues not tissue specific[1]	12,751	13,368	13,241	13,391	69.5	72.8	72.1	72.9
All homoeologues same tissue specificity[2]	1,556	1,938	1,868	1,798	8.5	10.6	10.2	9.8
D tissue specific, A and B not tissue specific[3]	1204	381	399	394	6.6	2.1	2.2	2.1
B tissue specific, A and D not tissue specific[3]	516	476	495	523	2.8	2.6	2.7	2.8
A tissue specific, B and D not tissue specific[3]	473	422	482	481	2.6	2.3	2.6	2.6
A and B same tissue specificity, D other tissue specificity[4]	119	137	98	99	0.6	0.7	0.5	0.5
A and B same tissue specificity, D not tissue specific[5]	351	227	245	228	1.9	1.2	1.3	1.2
A and D same tissue specificity, B other tissue specificity[4]	115	153	168	158	0.6	0.8	0.9	0.9
A and D same tissue specificity, B not tissue specific[5]	259	263	277	277	1.4	1.4	1.5	1.5
B and D same tissue specificity, A other tissue specificity[4]	114	156	169	167	0.6	0.8	0.9	0.9
B and D same tissue specificity, A not tissue specific[5]	269	259	293	279	1.5	1.4	1.6	1.5
All homoeologues showing varied tissue specificty[6]	630	577	622	562	3.4	3.1	3.4	3.1
Total	18,357	18,357	18,357	18,357	100.0	100.0	100.0	100.0

*[1]All three homoeologues of the triads are broadly expressed without any tissue specificity; [2]All three homoeologues show expression specificity to the same tissue; [3]One of the three homoeologues is tissue specific and the other two are broadly expressed; [4]Two of the three homoeologues show expression specificity in the same tissue and the third homoeologue shows expression specificity in another tissue; [5]Two of the three homoeologues show expression specificity in the same tissue and the third homoeologue is broadly expressed; [6]All homoeologues showed varied tissue specificity or wider expression. *Percentage of triads relative to the total number of triads (18,357).*

Supplementary Table 7. Summary of the number of triads with varied tissue specificity of homoeologues and expression bias patterns across the ten tissues in the four SHW lines

SHW	Homoeologues showing tissue specificity in triads	Bias pattern	Pistil-1DAA	Pistil-AI	Palea+ Lemma	Pistil-AM	Root	Shoot	Anther	Boot	Glume	Hypocotyl
**C44**	**A,B,D**	**Total**	**13**	**23**	**6**	**18**	**653**	**24**	**600**	**140**	**14**	**65**
C44	A,B,D	AB-bias	5	6	1	5	244	6	236	33	5	16
C44	A,B,D	D-bias	7	2	2	1	125	6	143	32	4	6
C44	A,B,D	UN-biased	1	15	3	12	284	12	221	75	5	43
**C44**	**A and B**	**Total**	**16**	**20**	**6**	**20**	**219**	**15**	**77**	**49**	**8**	**40**
C44	A and B	AB-bias	9	3	1	2	59	4	17	3	1	8
C44	A and B	D-bias	2	2	0	1	13	2	3	3	1	3
C44	A and B	UN-biased	5	15	5	17	147	9	57	43	6	29
**C44**	**A and D**	**Total**	**14**	**21**	**4**	**11**	**146**	**29**	**72**	**37**	**12**	**28**
C44	A and D	AB-bias	7	4	2	2	41	6	29	7	5	4
C44	A and D	D-bias	3	4	1	2	42	7	18	9	1	3
C44	A and D	UN-biased	4	13	1	7	63	16	25	21	6	21
**C44**	**B and D**	**Total**	**19**	**11**	**7**	**10**	**134**	**28**	**84**	**47**	**10**	**33**
C44	B and D	AB-bias	4	2	1	4	46	5	27	6	4	7
C44	B and D	D-bias	8	5	2	1	33	7	26	7	2	3
C44	B and D	UN-biased	7	4	4	5	55	16	31	34	4	23
**C44**	**A only**	**Total**	**60**	**80**	**59**	**82**	**231**	**92**	**176**	**112**	**72**	**100**
C44	A only	AB-bias	6	12	8	5	57	10	34	8	8	13
C44	A only	D-bias	11	19	8	16	16	15	9	16	13	4
C44	A only	UN-biased	43	49	43	61	158	67	133	88	51	83
**C44**	**B only**	**Total**	**77**	**78**	**47**	**71**	**266**	**78**	**188**	**90**	**60**	**140**
C44	B only	AB-bias	20	10	9	9	51	8	27	13	6	17
C44	B only	D-bias	11	14	4	14	38	15	25	6	11	13
C44	B only	UN-biased	46	54	34	48	177	55	136	71	43	110
**C44**	**D only**	**Total**	**158**	**225**	**116**	**163**	**241**	**189**	**204**	**159**	**121**	**164**
C44	D only	AB-bias	104	181	76	110	114	85	100	72	91	98
C44	D only	D-bias	20	4	8	9	33	31	30	20	6	7
C44	D only	UN-biased	34	40	32	44	94	73	74	67	24	59
**C45**	**A,B,D**	**Total**	**58**	**33**	**21**	**8**	**838**	**52**	**527**	**268**	**10**	**123**
C45	A,B,D	AB-bias	16	2	3	0	178	6	159	43	2	24
C45	A,B,D	D-bias	17	2	7	4	236	5	146	64	1	23
C45	A,B,D	UN-biased	25	29	11	4	424	41	222	161	7	76
**C45**	**A and B**	**Total**	**25**	**21**	**8**	**10**	**127**	**37**	**37**	**50**	**6**	**43**
C45	A and B	AB-bias	7	4	1	3	33	5	13	7	2	12
C45	A and B	D-bias	3	1	1	1	17	6	8	6	1	8
C45	A and B	UN-biased	15	16	6	6	77	26	16	37	3	23
**C45**	**A and D**	**Total**	**25**	**22**	**10**	**5**	**157**	**51**	**46**	**57**	**5**	**38**
C45	A and D	AB-bias	3	5	2	2	28	8	9	11	0	9
C45	A and D	D-bias	9	3	0	1	52	12	14	16	0	11
C45	A and D	UN-biased	13	14	8	2	77	31	23	30	5	18
**C45**	**B and D**	**Total**	**24**	**14**	**9**	**5**	**165**	**38**	**39**	**61**	**6**	**54**

C45	B and D	AB-bias	6	2	2	1	24	3	8	8	0	3
C45	B and D	D-bias	4	2	3	3	64	9	15	14	0	15
C45	B and D	UN-biased	14	10	4	1	77	26	16	39	6	36
**C45**	**A only**	**Total**	**89**	**81**	**78**	**61**	**199**	**99**	**110**	**124**	**42**	**114**
C45	A only	AB-bias	15	8	13	7	36	8	31	29	5	17
C45	A only	D-bias	15	20	14	13	40	10	11	22	7	14
C45	A only	UN-biased	59	53	51	41	123	81	68	73	30	83
**C45**	**B only**	**Total**	**86**	**80**	**81**	**53**	**237**	**130**	**99**	**135**	**34**	**126**
C45	B only	AB-bias	18	8	3	8	42	13	18	20	0	26
C45	B only	D-bias	14	20	18	9	43	13	14	29	4	15
C45	B only	UN-biased	54	52	60	36	152	104	67	86	30	85
**C45**	**D only**	**Total**	**69**	**64**	**76**	**50**	**218**	**105**	**86**	**105**	**52**	**104**
C45	D only	AB-bias	6	15	9	11	30	11	9	14	10	11
C45	D only	D-bias	19	9	12	6	61	14	33	26	9	22
C45	D only	UN-biased	44	40	55	33	127	80	44	65	33	71
**C65**	**A,B,D**	**Total**	**82**	**22**	**10**	**15**	**856**	**53**	**468**	**237**	**8**	**117**
C65	A,B,D	AB-bias	19	2	1	4	189	3	141	27	2	29
C65	A,B,D	D-bias	16	4	4	2	251	6	107	41	1	21
C65	A,B,D	UN-biased	47	16	5	9	416	44	220	169	5	67
**C65**	**A and B**	**Total**	**22**	**11**	**6**	**13**	**142**	**38**	**33**	**33**	**9**	**36**
C65	A and B	AB-bias	6	3	1	3	38	3	12	6	1	10
C65	A and B	D-bias	4	2	0	1	25	6	7	4	3	7
C65	A and B	UN-biased	12	6	5	9	79	29	14	23	5	19
**C65**	**A and D**	**Total**	**34**	**25**	**10**	**8**	**181**	**41**	**48**	**57**	**4**	**37**
C65	A and D	AB-bias	5	1	2	3	37	4	9	10	1	10
C65	A and D	D-bias	8	4	3	0	59	12	24	10	1	11
C65	A and D	UN-biased	21	20	5	5	85	25	15	37	2	16
**C65**	**B and D**	**Total**	**32**	**19**	**8**	**10**	**192**	**33**	**44**	**68**	**6**	**50**
C65	B and D	AB-bias	5	4	1	2	35	5	8	8	1	4
C65	B and D	D-bias	11	2	1	3	83	11	21	13	1	18
C65	B and D	UN-biased	16	13	6	5	74	17	15	47	4	28
**C65**	**A only**	**Total**	**88**	**97**	**81**	**54**	**221**	**117**	**120**	**173**	**45**	**133**
C65	A only	AB-bias	15	8	8	6	50	16	28	34	4	23
C65	A only	D-bias	10	27	17	7	46	17	13	31	12	31
C65	A only	UN-biased	63	62	56	41	125	84	79	108	29	79
**C65**	**B only**	**Total**	**100**	**84**	**68**	**67**	**244**	**106**	**110**	**137**	**63**	**125**
C65	B only	AB-bias	14	12	10	7	46	10	26	21	6	30
C65	B only	D-bias	9	16	15	5	55	6	10	20	14	20
C65	B only	UN-biased	77	56	43	55	143	90	74	96	43	75
**C65**	**D only**	**Total**	**78**	**75**	**55**	**52**	**224**	**94**	**94**	**128**	**51**	**101**
C65	D only	AB-bias	9	12	9	9	38	11	10	19	11	23
C65	D only	D-bias	17	12	10	7	67	17	34	27	11	20
C65	D only	UN-biased	52	51	36	36	119	66	50	82	29	58
**C66**	**A,B,D**	**Total**	**65**	**26**	**11**	**15**	**781**	**29**	**503**	**254**	**5**	**109**
C66	A,B,D	AB-bias	13	6	0	1	118	3	123	30	1	26
C66	A,B,D	D-bias	16	3	3	7	184	6	136	56	2	16
C66	A,B,D	UN-biased	36	17	8	7	479	20	244	168	2	67

**C66**	**A and B**	**Total**	**24**	**24**	**10**	**6**	**120**	**29**	**36**	**33**	**6**	**39**
C66	A and B	AB-bias	9	2	1	1	27	4	13	4	1	8
C66	A and B	D-bias	1	2	1	1	18	4	7	3	1	5
C66	A and B	UN-biased	14	20	8	4	75	21	16	26	4	26
**C66**	**A and D**	**Total**	**23**	**22**	**9**	**17**	**156**	**32**	**48**	**73**	**11**	**44**
C66	A and D	AB-bias	4	3	1	3	20	1	11	13	6	4
C66	A and D	D-bias	4	3	2	2	42	9	21	20	0	11
C66	A and D	UN-biased	15	16	6	12	94	22	16	40	5	29
**C66**	**B and D**	**Total**	**27**	**20**	**10**	**13**	**174**	**34**	**39**	**69**	**8**	**52**
C66	B and D	AB-bias	4	2	1	3	23	3	11	5	0	3
C66	B and D	D-bias	12	2	3	2	49	4	18	15	1	16
C66	B and D	UN-biased	11	16	6	8	102	27	10	49	7	33
**C66**	**A only**	**Total**	**100**	**95**	**61**	**68**	**205**	**107**	**111**	**126**	**53**	**130**
C66	A only	AB-bias	10	7	9	6	34	10	31	20	5	18
C66	A only	D-bias	20	23	10	14	30	11	12	20	7	18
C66	A only	UN-biased	70	65	42	48	141	86	68	86	41	94
**C66**	**B only**	**Total**	**116**	**85**	**46**	**58**	**229**	**110**	**113**	**135**	**52**	**144**
C66	B only	AB-bias	25	10	2	6	41	12	24	15	3	18
C66	B only	D-bias	12	25	10	10	32	11	16	28	4	24
C66	B only	UN-biased	79	50	34	42	156	87	73	92	45	102
**C66**	**D only**	**Total**	**83**	**86**	**44**	**55**	**211**	**84**	**81**	**121**	**38**	**93**
C66	D only	AB-bias	11	25	4	7	19	9	7	19	11	15
C66	D only	D-bias	20	8	4	12	54	22	25	17	3	19
C66	D only	UN-biased	52	53	36	36	138	53	49	85	24	59

Pistil-1DAA: pistil-one day after anthesis; Pistil-AM: pistil-when anthers are at mature stage; Pistil-AI: pistil-when anthers are at immature stage; Boot: head at boot stage.

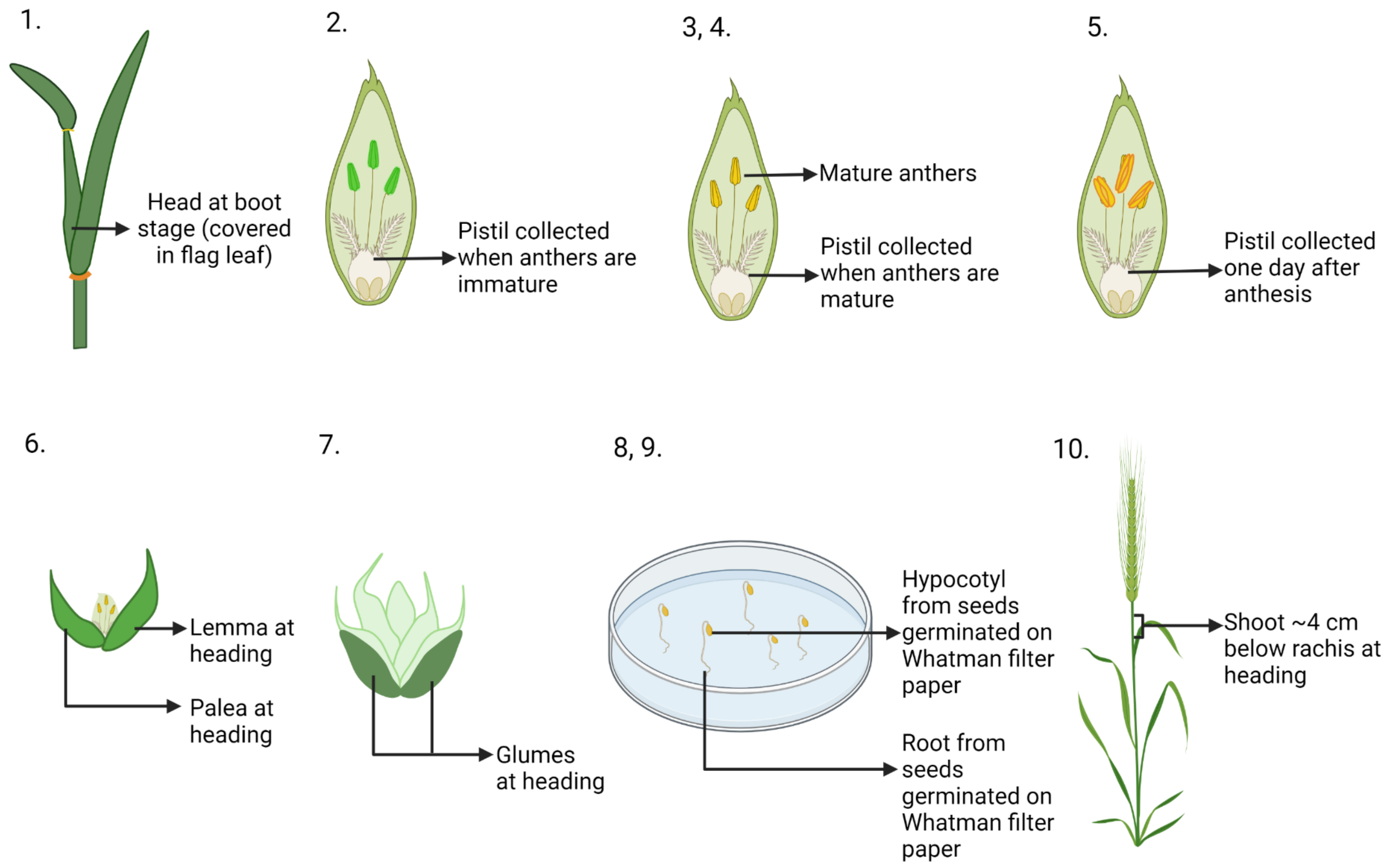

Supplementary Figure 1. Pictorial representation of the ten different tissues used in the study. (1) Head at boot stage; (2) Pistil when anthers are green and immature; (3) Mature anthers; (4) Pistil when anthers are mature; (5) Pistil-one day after anthesis; (6) Palea+Lemma; (7) Glumes; (8) Hypocotyl; (9) Root; (10) Shoot. Figure created with Biorender.com.

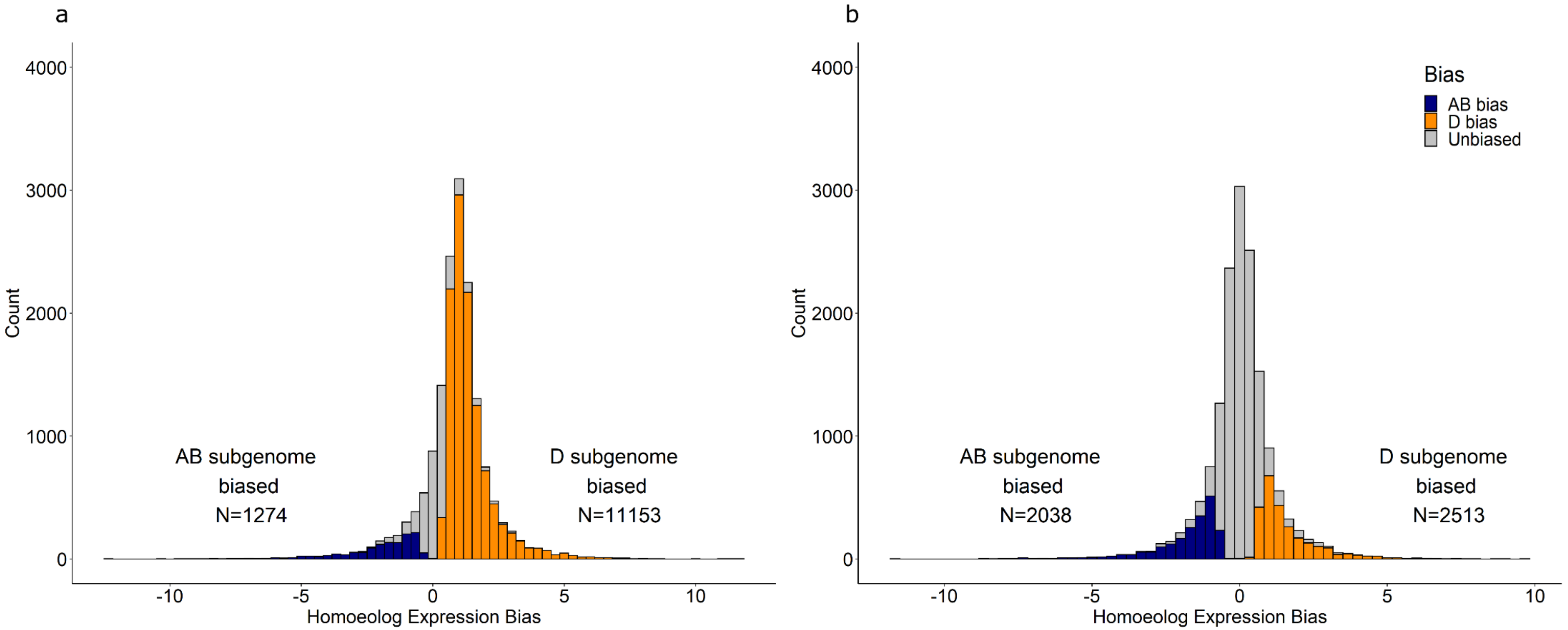

Supplementary Figure 2. Distribution of homoeologue expression bias in pistil-one day after anthesis of SHW-C66. (a) Comparison of the expression bias of triads towards AB and D genomes of tetraploid (PI377655) and diploid (AS2386) parents; (b) Comparison of the expression bias of triads towards AB and D subgenomes of SHW-C66.

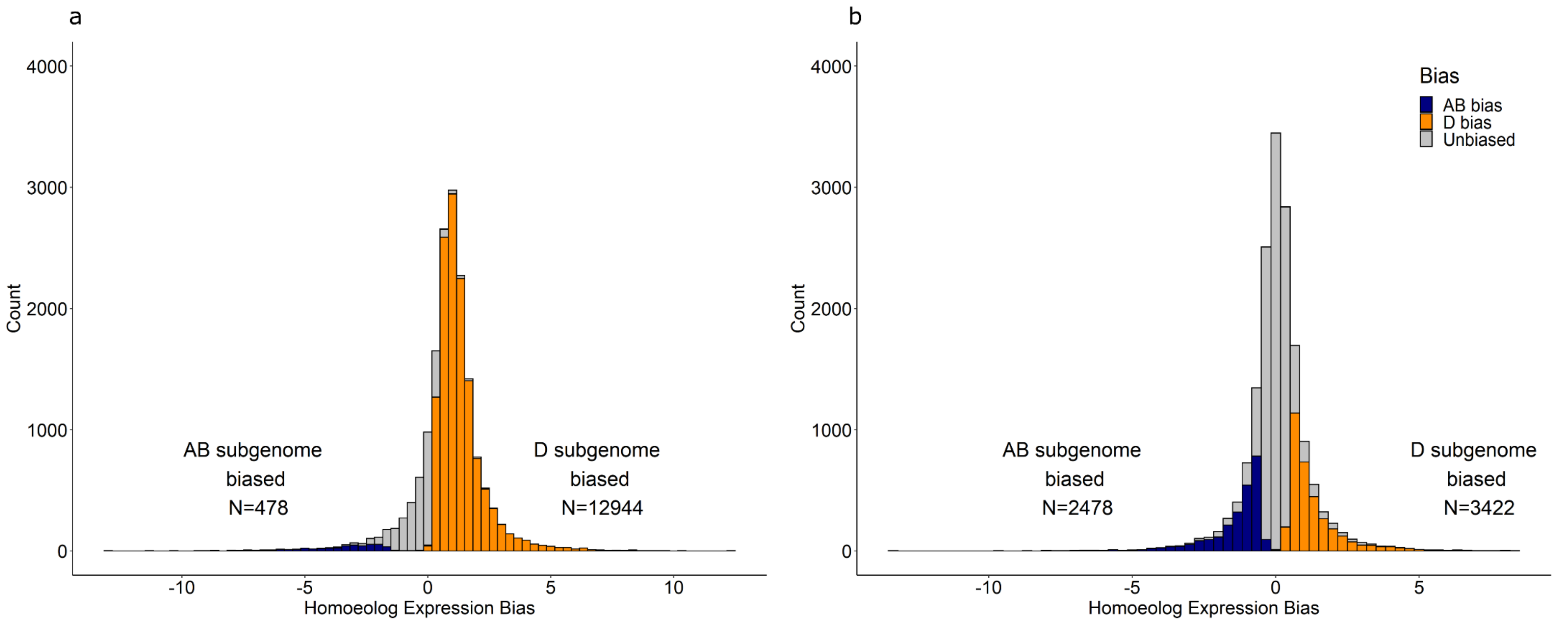

Supplementary Figure 3. Distribution of homoeologue expression bias in head collected at boot stage of SHW-C66. (a) Comparison of the expression bias of triads towards AB and D genomes of tetraploid (PI377655) and diploid (AS2386) parents; (b) Comparison of the expression bias of triads towards AB and D subgenomes of SHW-C66.

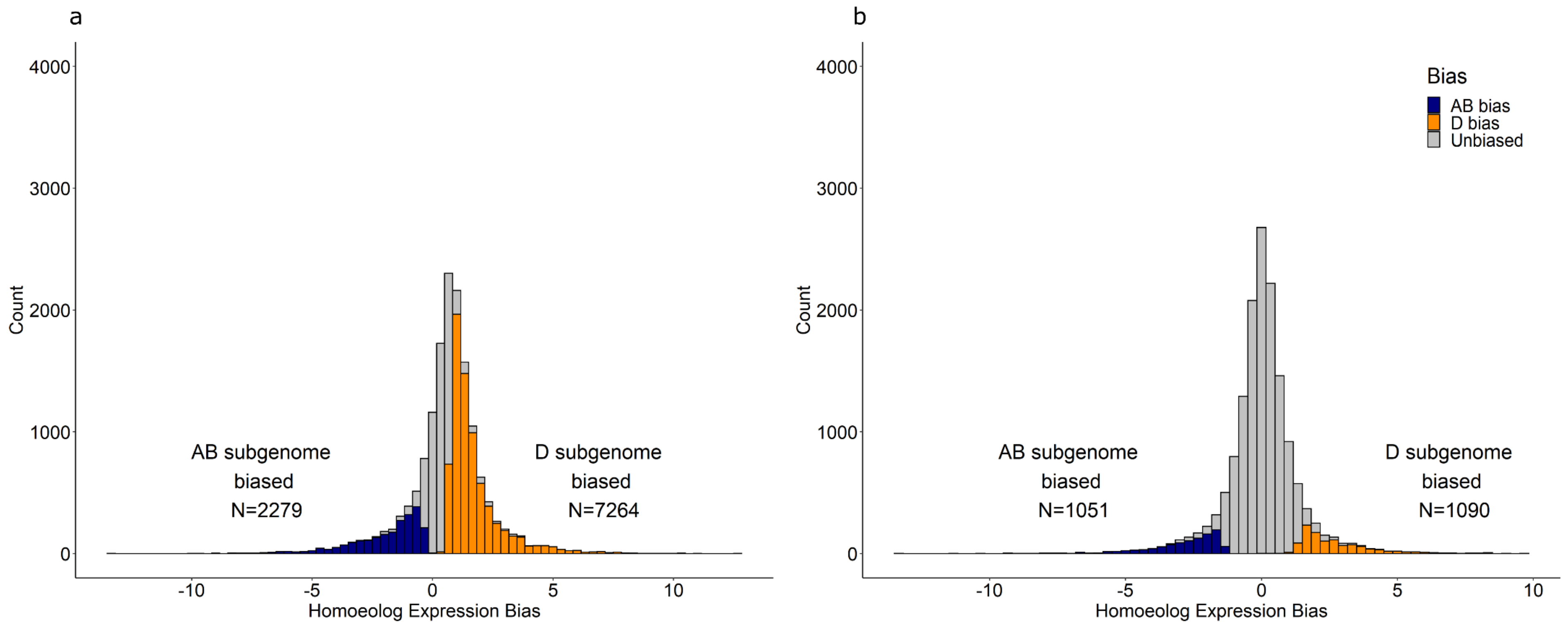

Supplementary Figure 4. Distribution of homoeologue expression bias in glume of SHW-C66. (a) Comparison of the expression bias of triads towards AB and D genomes of tetraploid (PI377655) and diploid (AS2386) parents; (b) Comparison of the expression bias of triads towards AB and D subgenomes of SHW-C66.

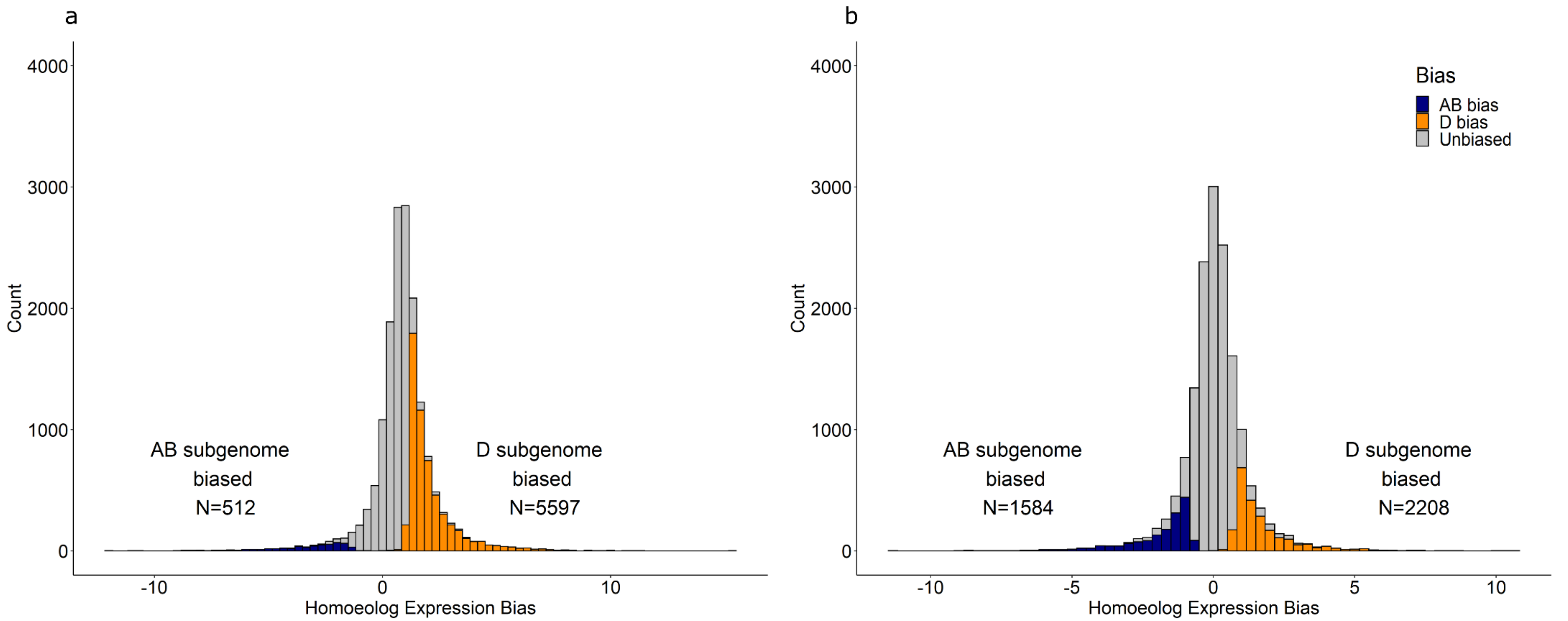

Supplementary Figure 5. Distribution of homoeologue expression bias in hypocotyl of SHW-C66. (a) Comparison of the expression bias of triads towards AB and D genomes of tetraploid (PI377655) and diploid (AS2386) parents; (b) Comparison of the expression bias of triads towards AB and D subgenomes of SHW-C66.

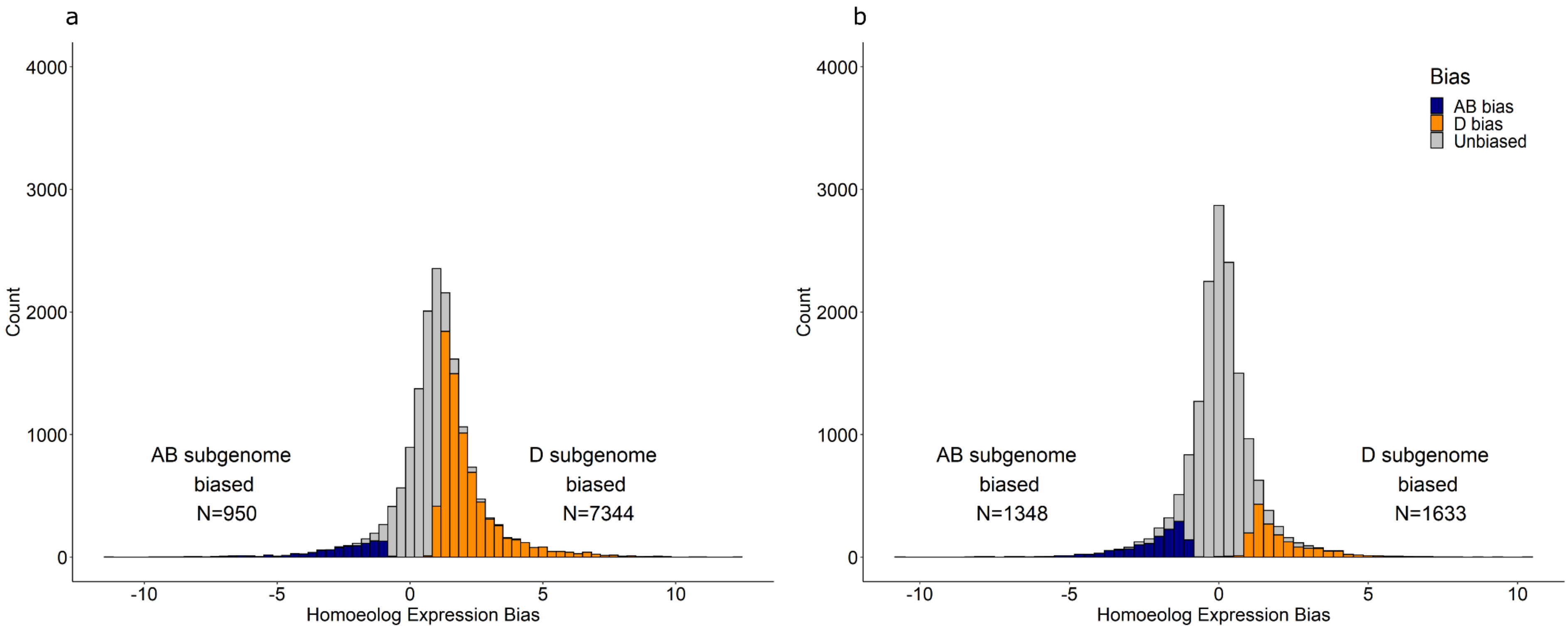

Supplementary Figure 6. Distribution of homoeologue expression bias in palea+lemma of SHW-C66. (a) Comparison of the expression bias of triads towards AB and D genomes of tetraploid (PI377655) and diploid (AS2386) parents; (b) Comparison of the expression bias of triads towards AB and D subgenomes of SHW-C66.

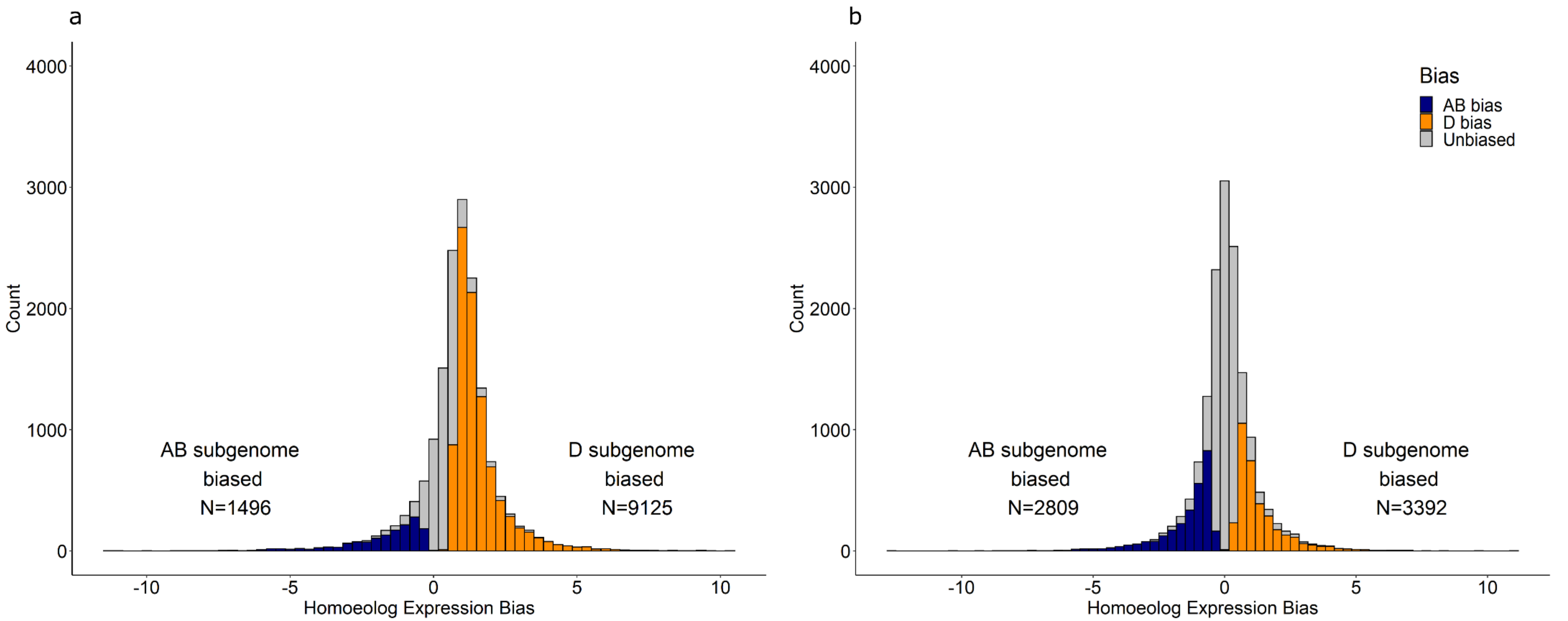

Supplementary Figure 7. Distribution of homoeologue expression bias in pistil (collected when anthers were mature) of SHW-C66. (a) Comparison of the expression bias of triads towards AB and D genomes of tetraploid (PI377655) and diploid (AS2386) parents; (b) Comparison of the expression bias of triads towards AB and D subgenomes of SHW-C66.

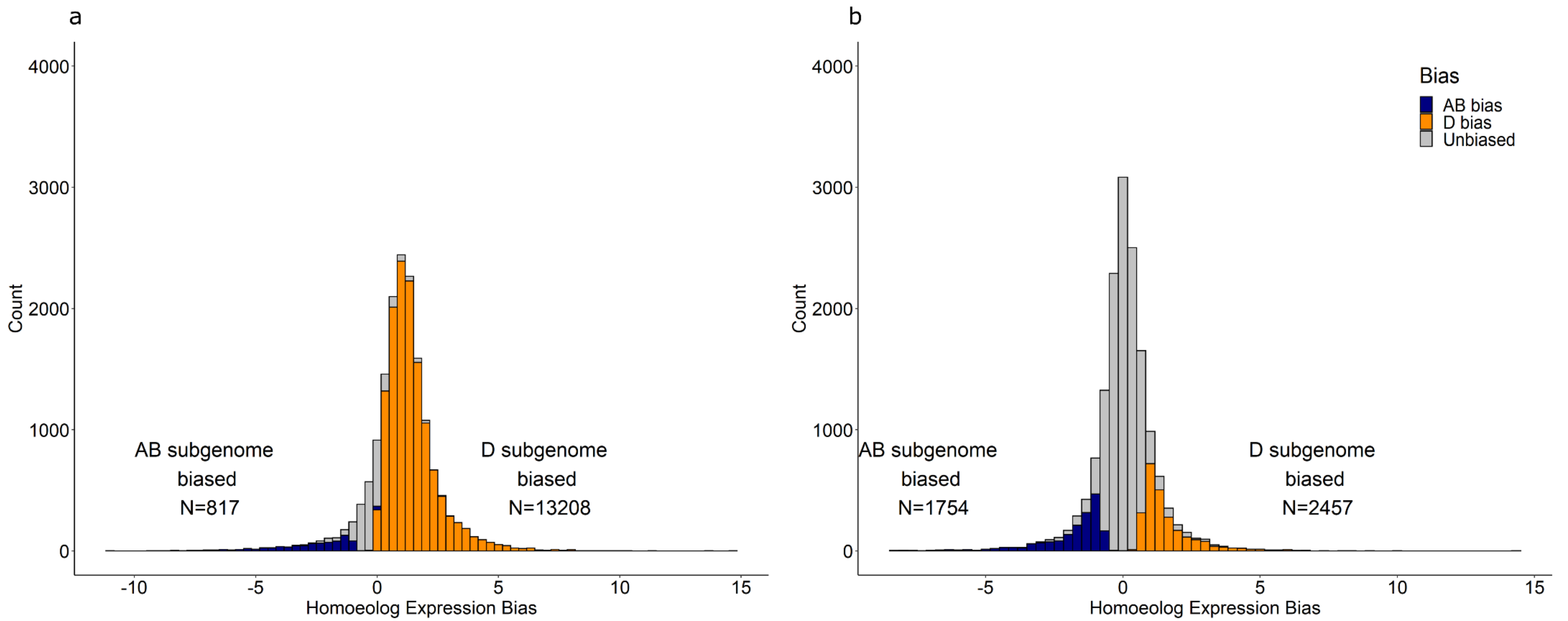

Supplementary Figure 8. Distribution of homoeologue expression bias in root of SHW-C66. (a) Comparison of the expression bias of triads towards AB and D genomes of tetraploid (PI377655) and diploid (AS2386) parents; (b) Comparison of the expression bias of triads towards AB and D subgenomes of SHW-C66.

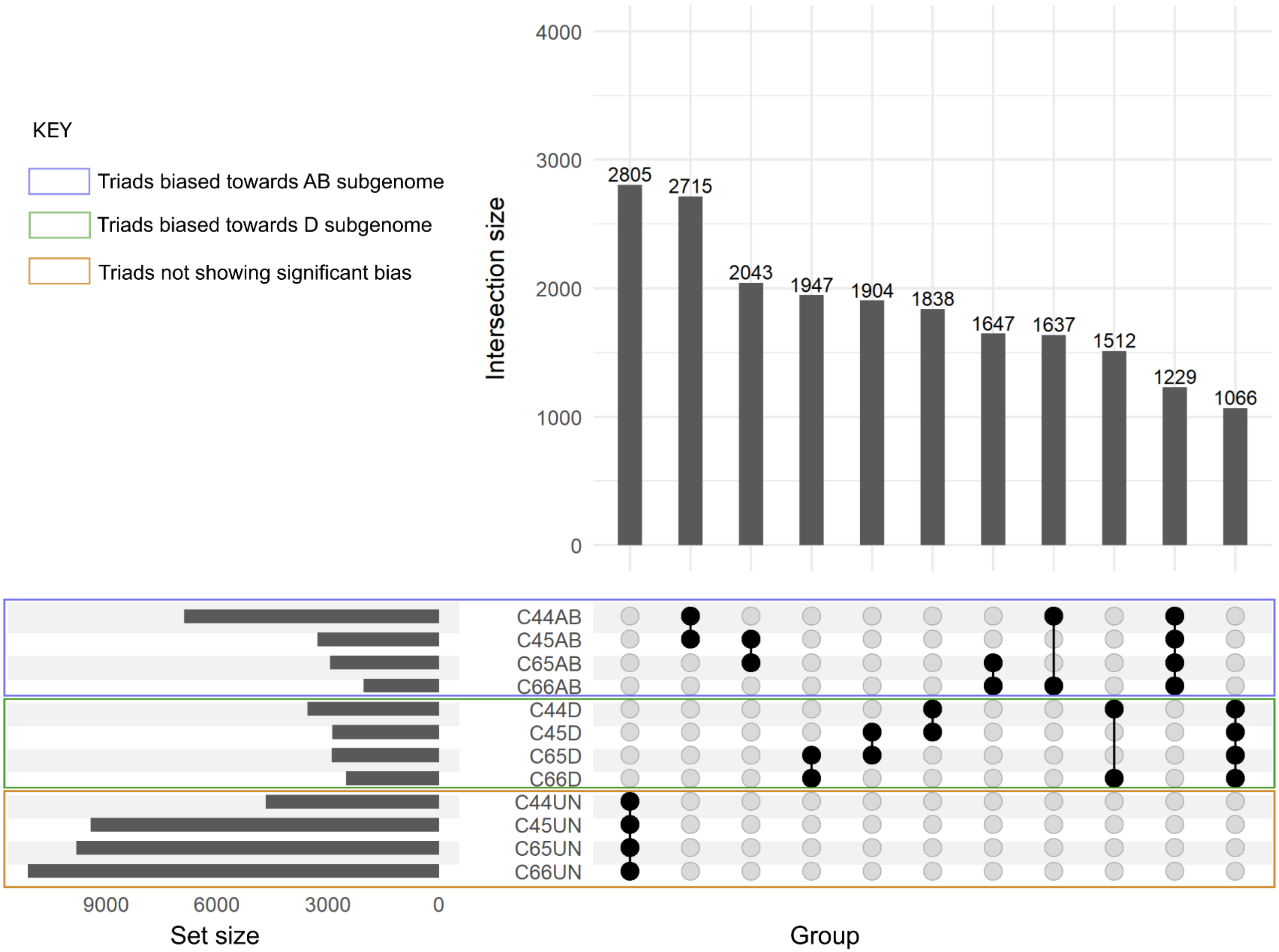

Supplementary Figure 9. Subsets of triads showing similar expression patterns across all four SHW lines in pistil-one day after anthesis.

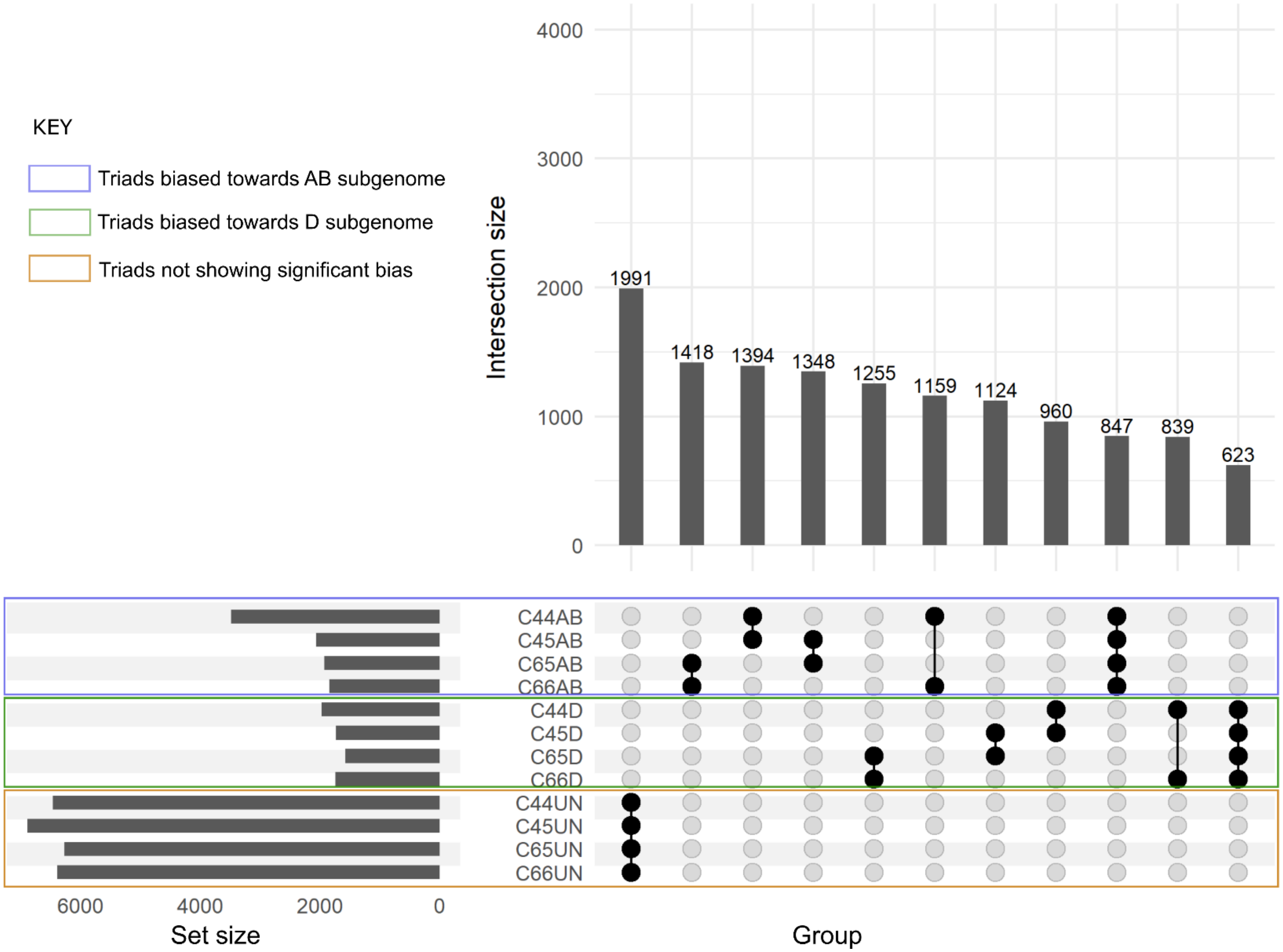

Supplementary Figure 10. Subsets of triads showing similar expression patterns across all four SHW lines in mature anther.

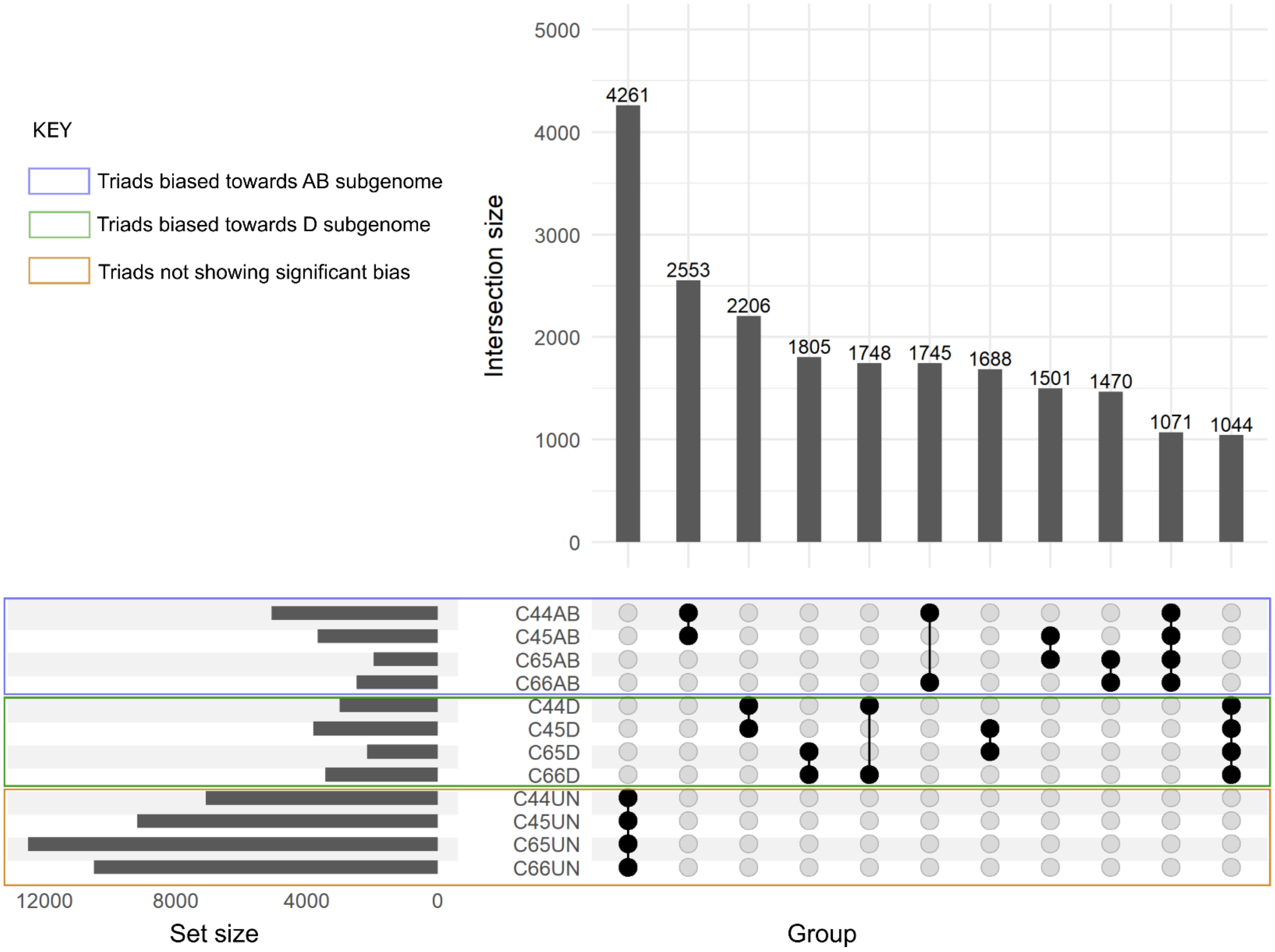

Supplementary Figure 11. Subsets of triads showing similar expression patterns across all four SHW lines in head collected at boot stage.

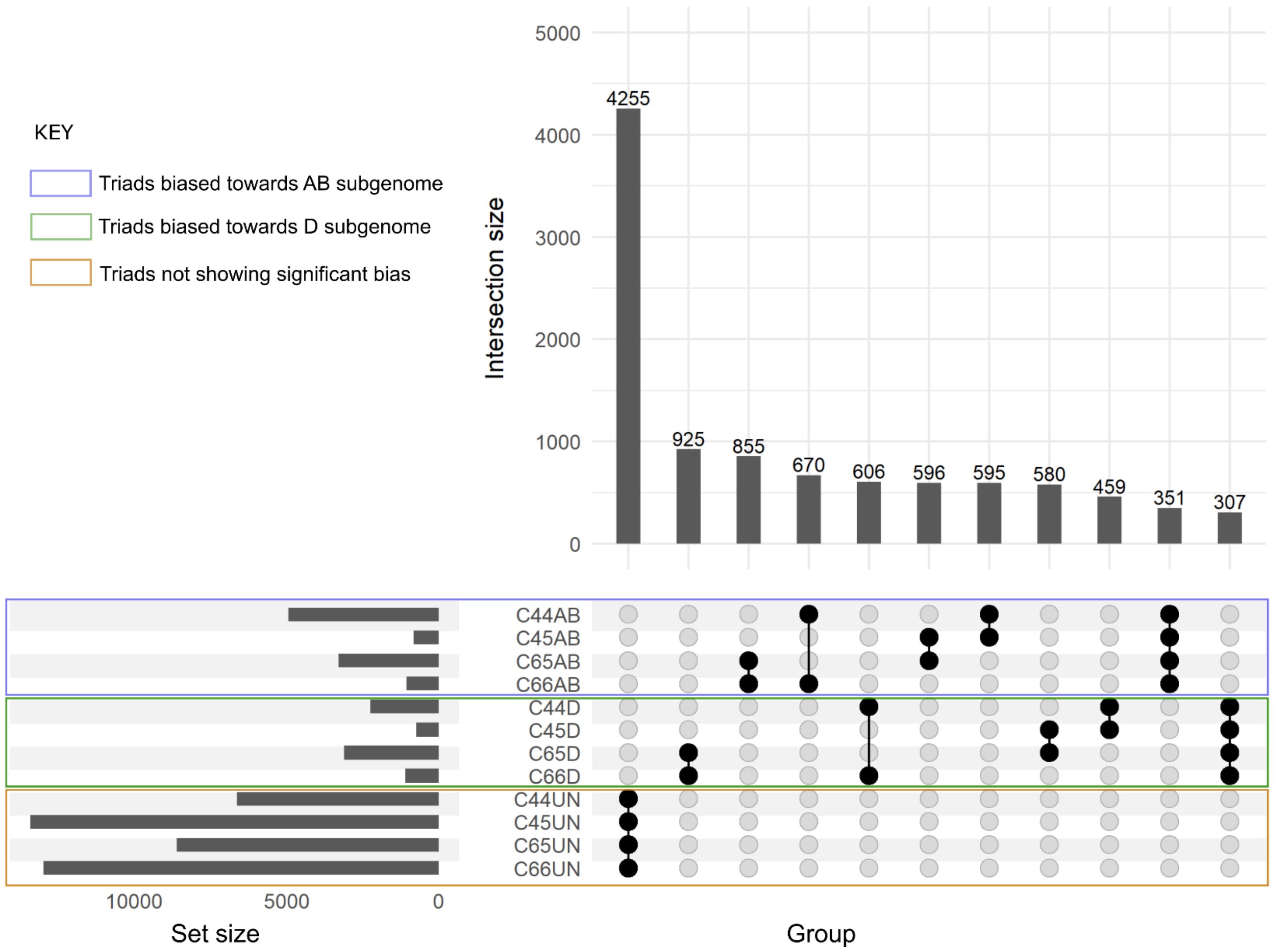

Supplementary Figure 12. Subsets of triads showing similar expression patterns across all four SHW lines in glume.

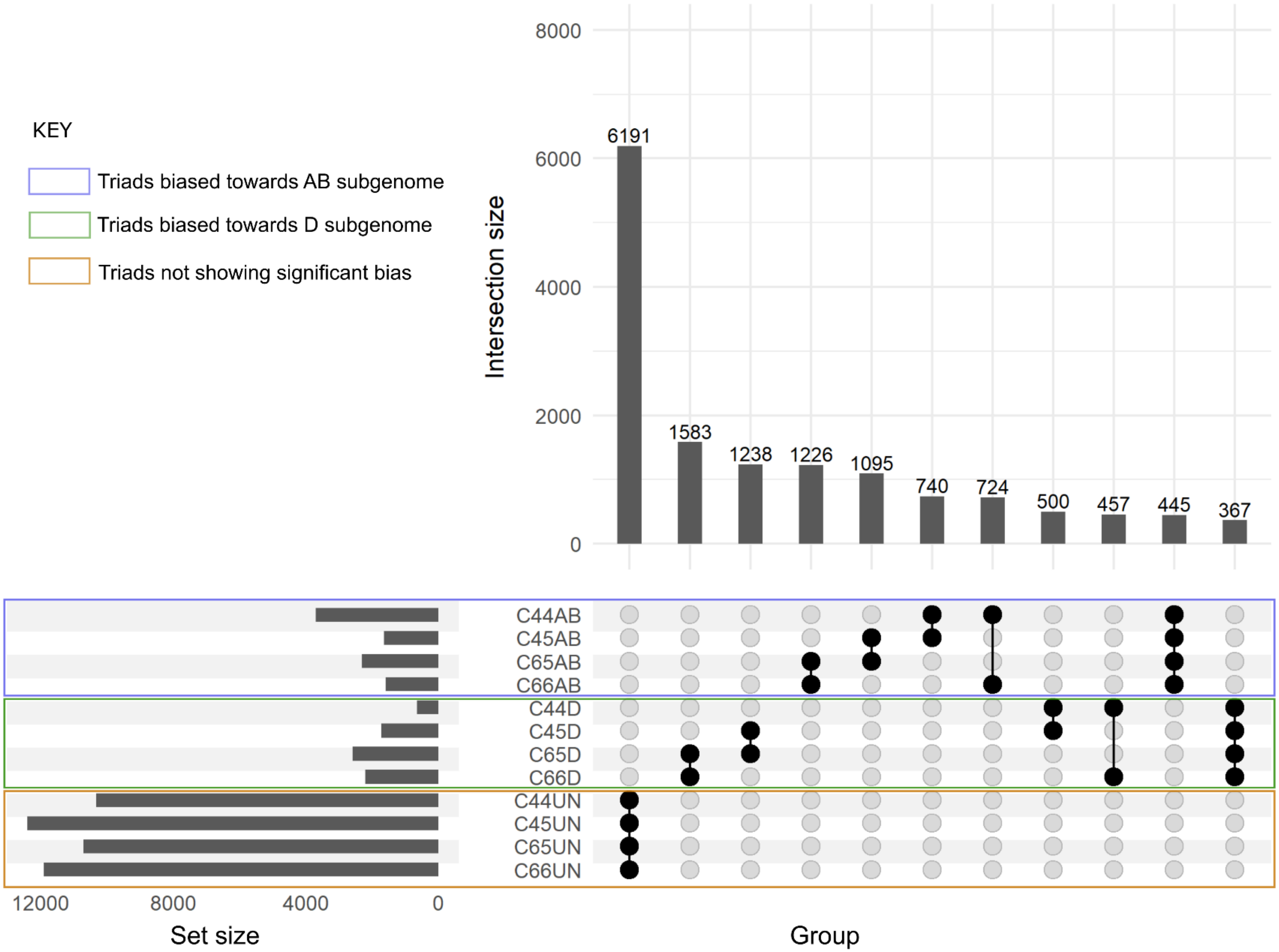

Supplementary Figure 13. Subsets of triads showing similar expression patterns across all four SHW lines in hypocotyl.

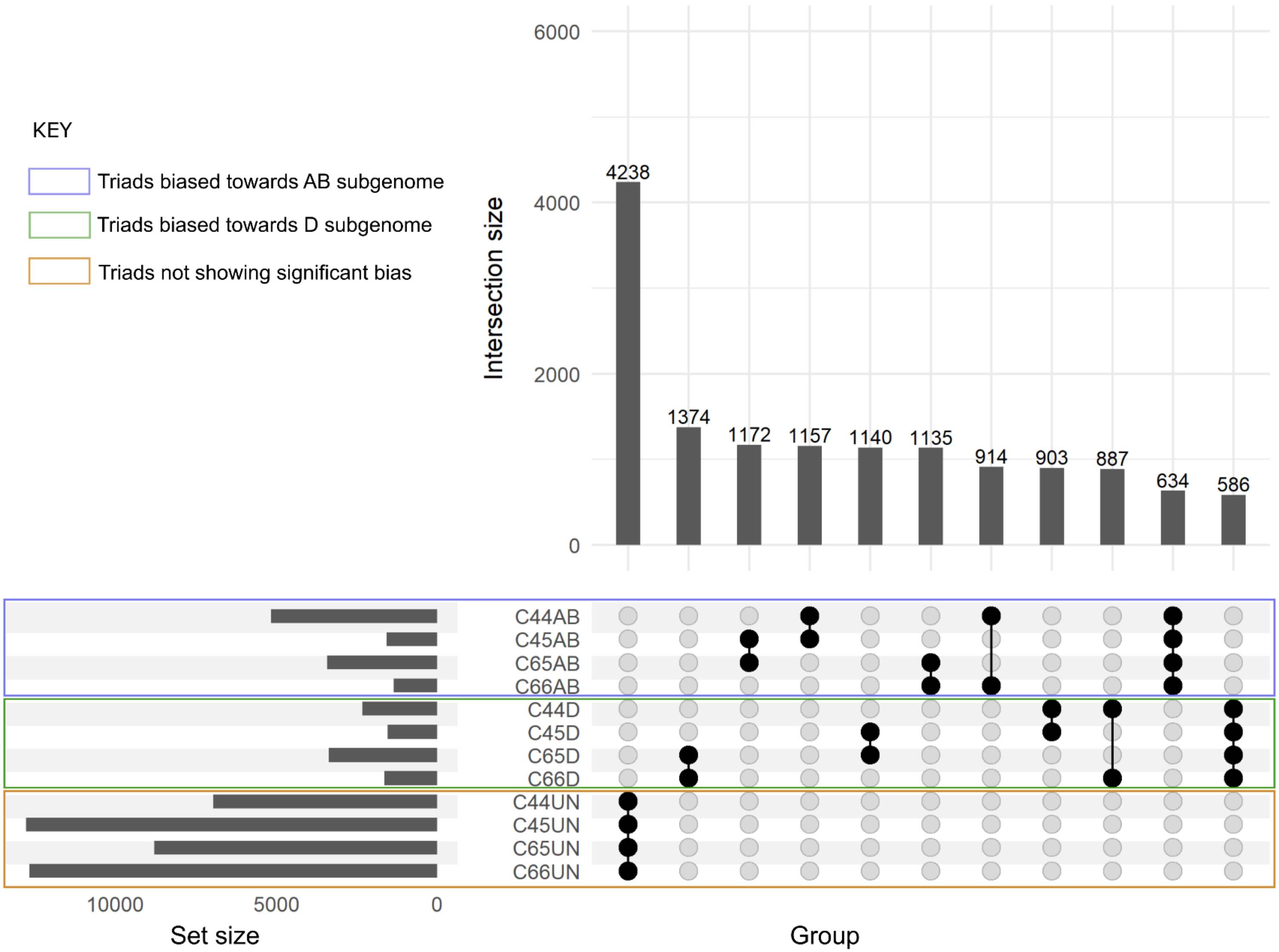

Supplementary Figure 14. Subsets of triads showing similar expression patterns across all four SHW lines in palea+lemma.

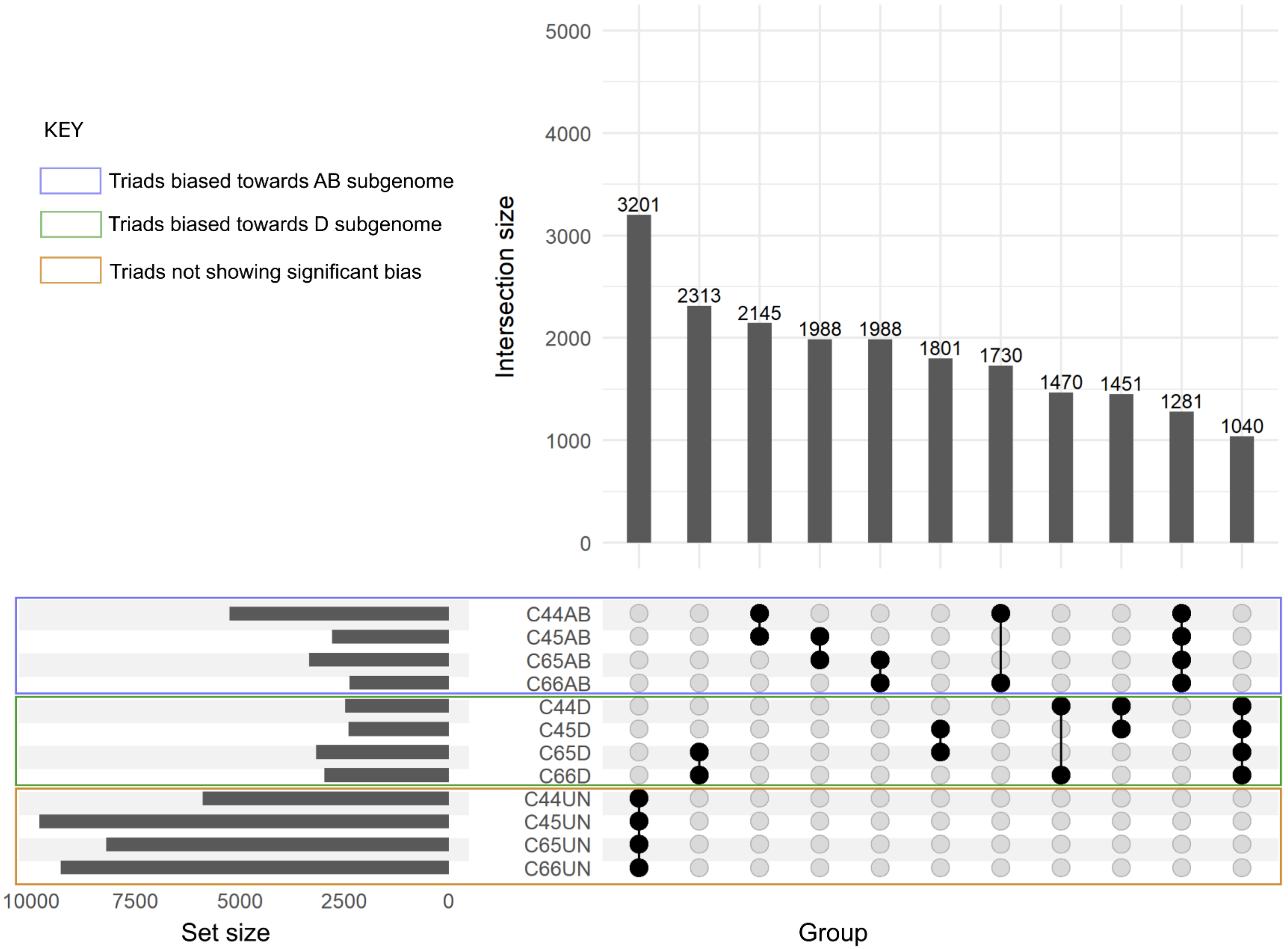

Supplementary Figure 15. Subsets of triads showing similar expression patterns across all four SHW lines in pistil-when anthers are green and immature.

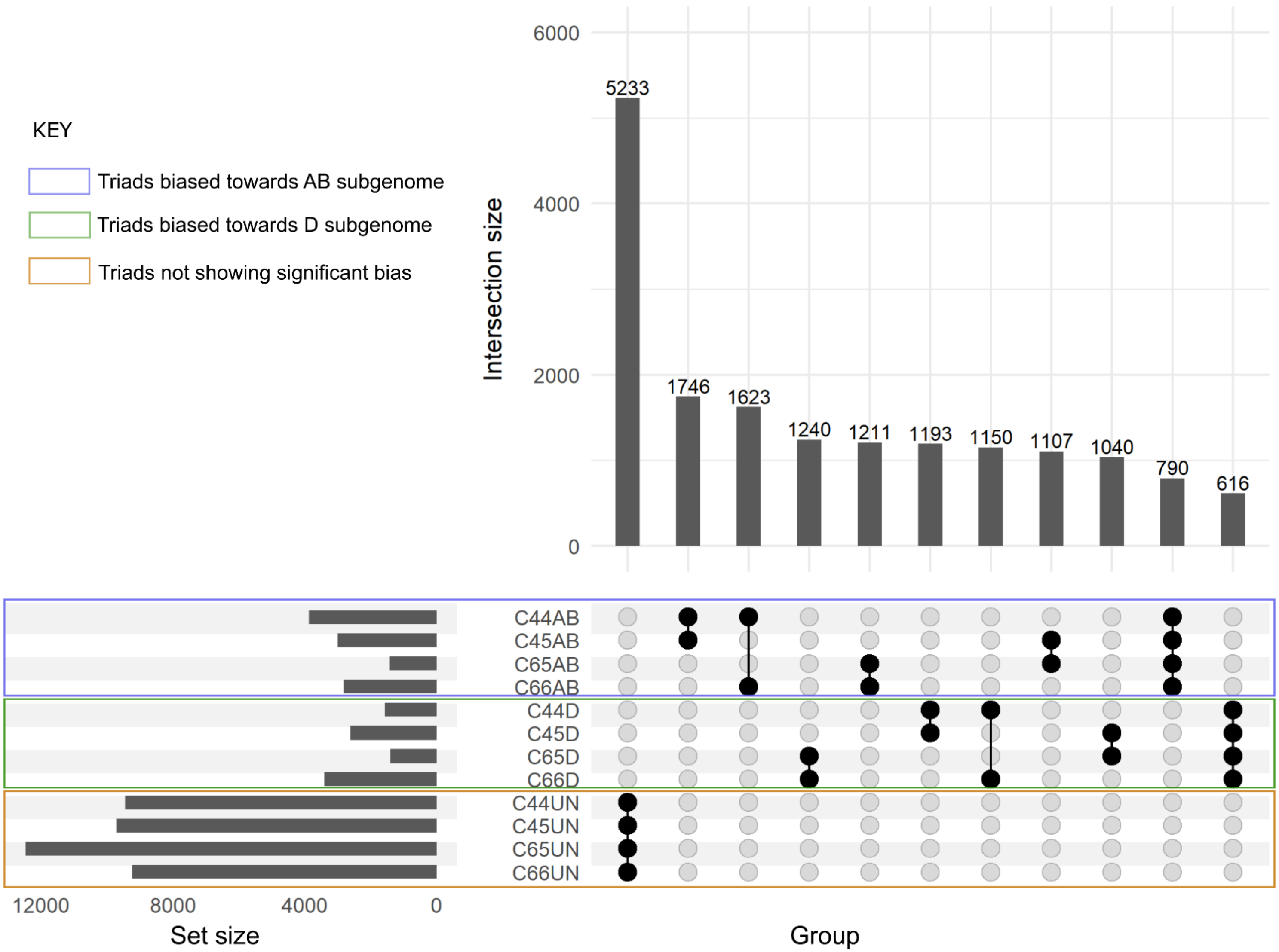

Supplementary Figure 16. Subsets of triads showing similar expression patterns across all four SHW lines in pistil-when anthers are yellow and just prior to dehiscence.

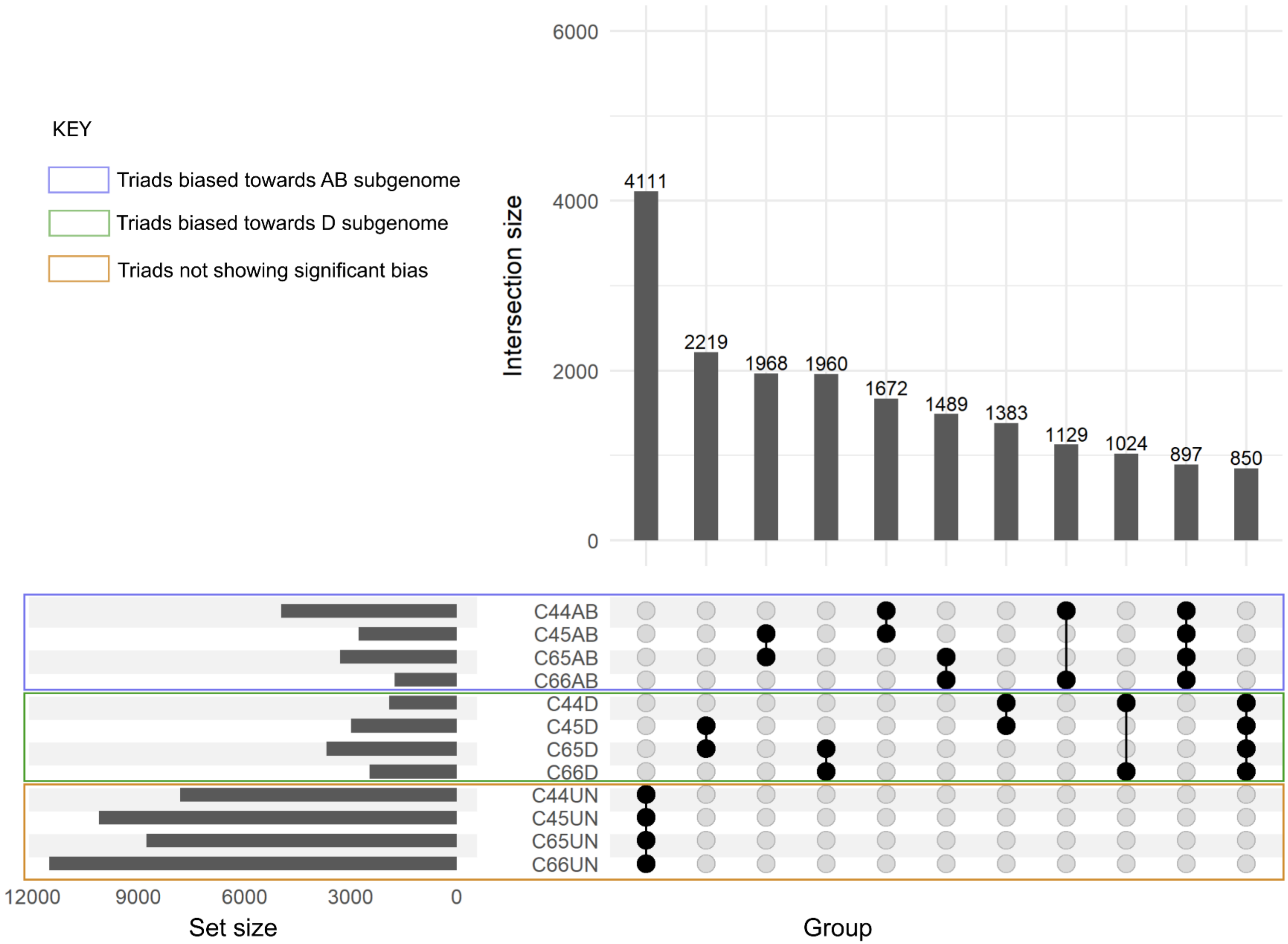

Supplementary Figure 17. Subsets of triads showing similar expression patterns across all four SHW lines in root.

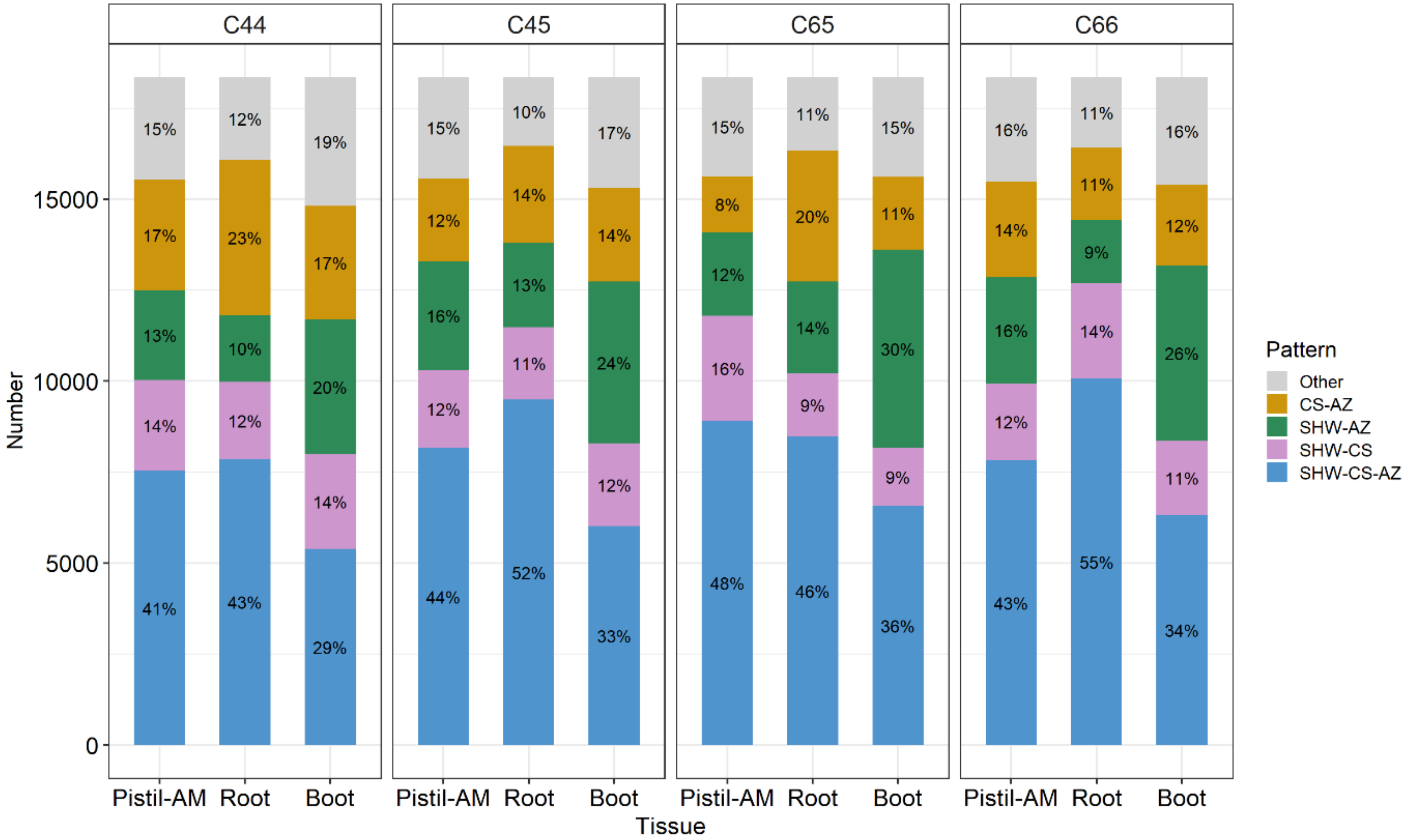

Supplementary Figure 18. The proportion of triads showing varied bias patterns between the SHWs (C44, C45, C65, C66), the landrace Chinese Spring and cultivar Azhurnaya. The different patterns represent the following: CS-AZ - triads showing similar pattern in Chinese Spring and Azhurnaya and not in the SHW line; SHW-AZ - triads showing reversal of bias in Azhurnaya to the same observed in SHW; SHW-CS - triads showing similar pattern in SHW and Chinese Spring and not in Azhurnaya; SHW-CS-AZ - triads showing similar patterns in SHW, Chinese Spring and Azhurnaya.

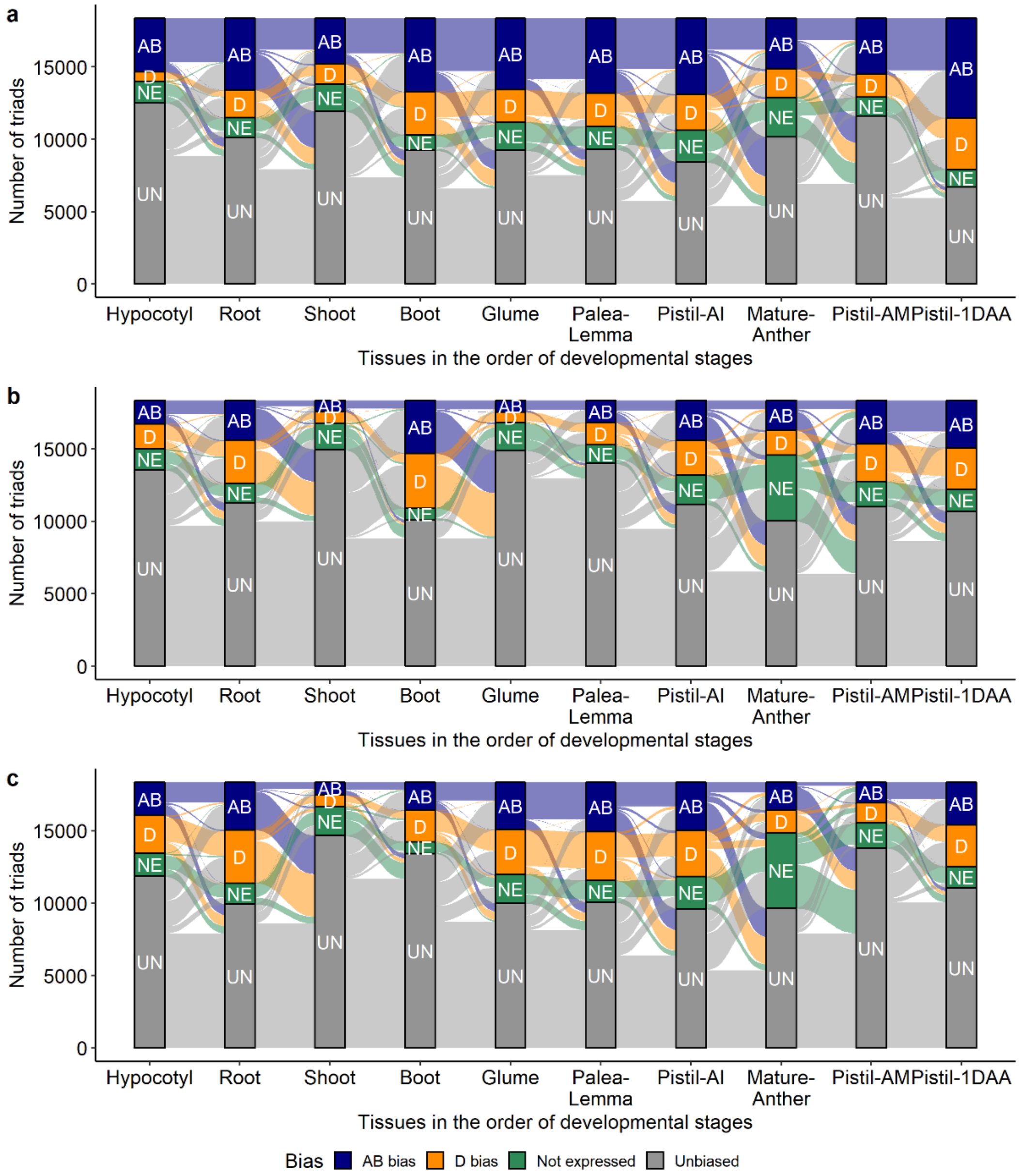

Supplementary Figure 19. Alluvial plots representing the expression bias trends of the triads across the tissues in the (a) SHW-C44 (b) SHW-C45 and (c) SHW-C65. AB bias (AB), represented by navy blue bars: triads significantly biased towards the AB subgenome; D bias (D), represented by dark orange bars: triads significantly biased towards the D subgenome; Not expressed (NE), represented by green bars: the homoeologues of the triads are not expressed; Unbiased (UN), represented by gray bars: no significant expression bias observed.

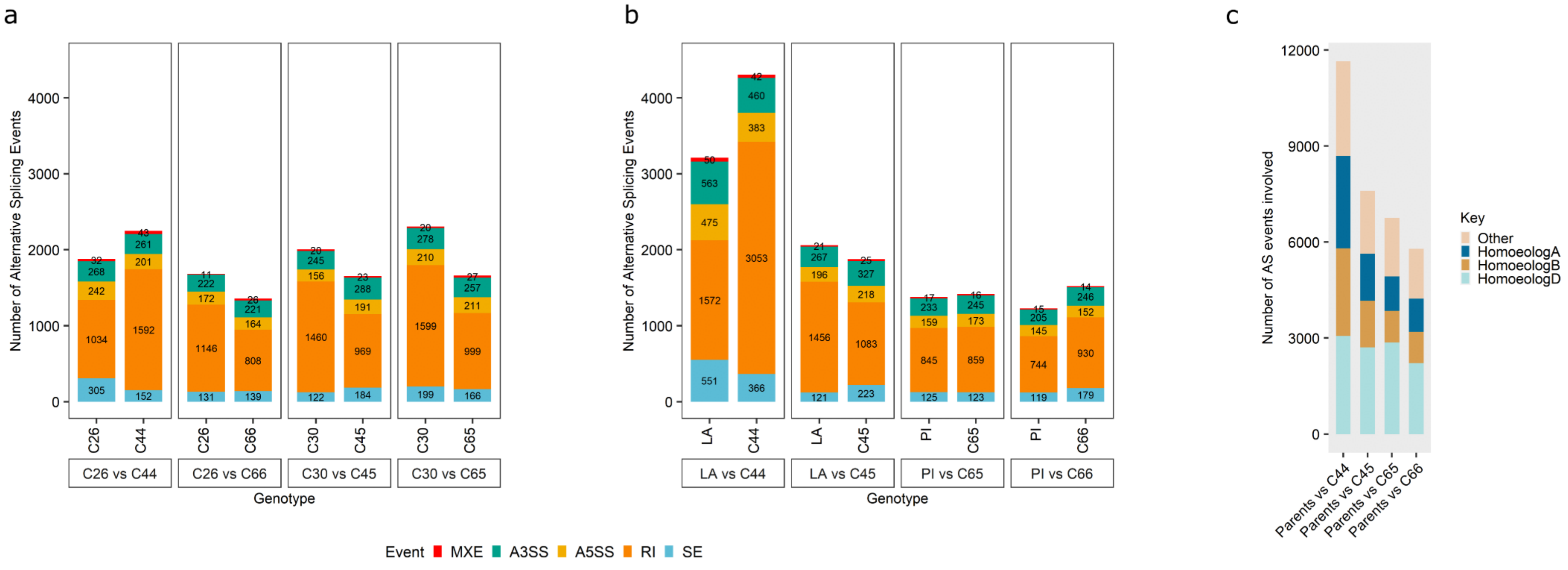

Supplementary Figure 20. Number of alternative splicing events detected in the Diploid/Tetraploid parent vs SHW comparison in pistil-one day after anthesis.

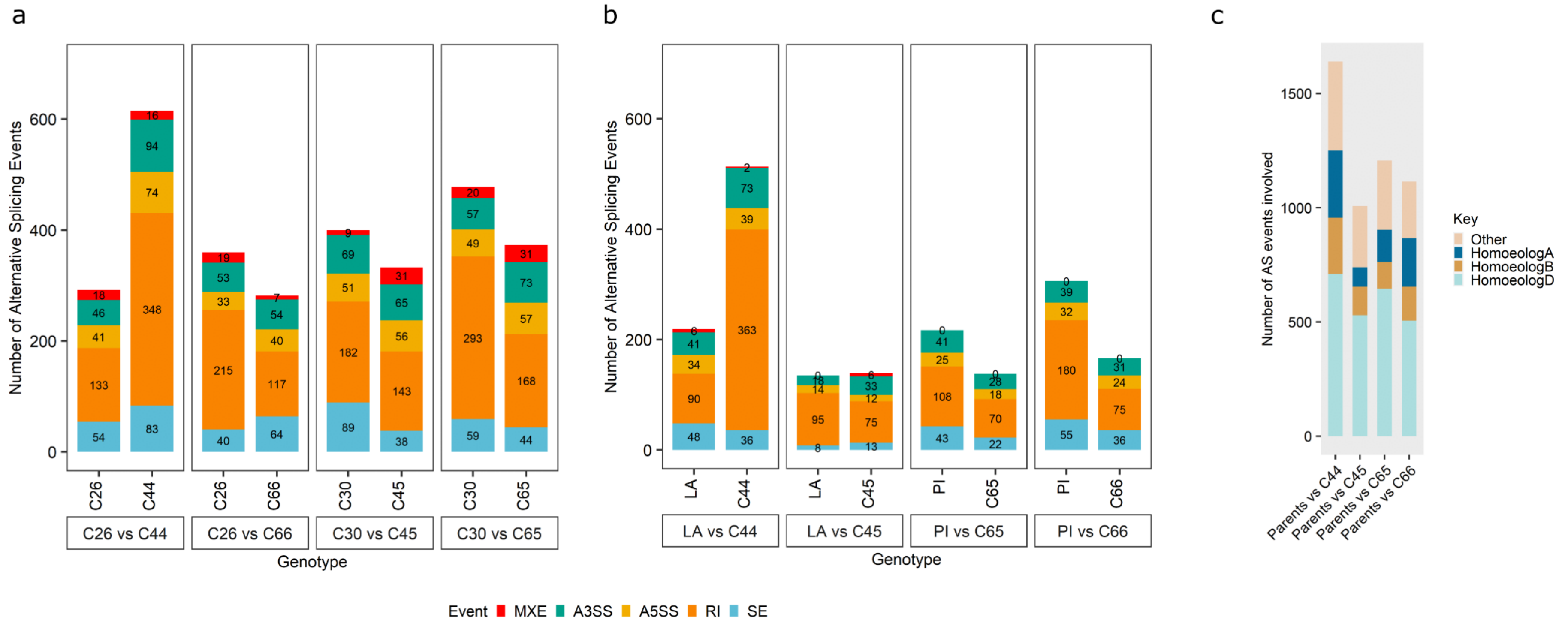

Supplementary Figure 21. Number of alternative splicing events detected in the Diploid/Tetraploid parent vs SHW comparison in mature anther.

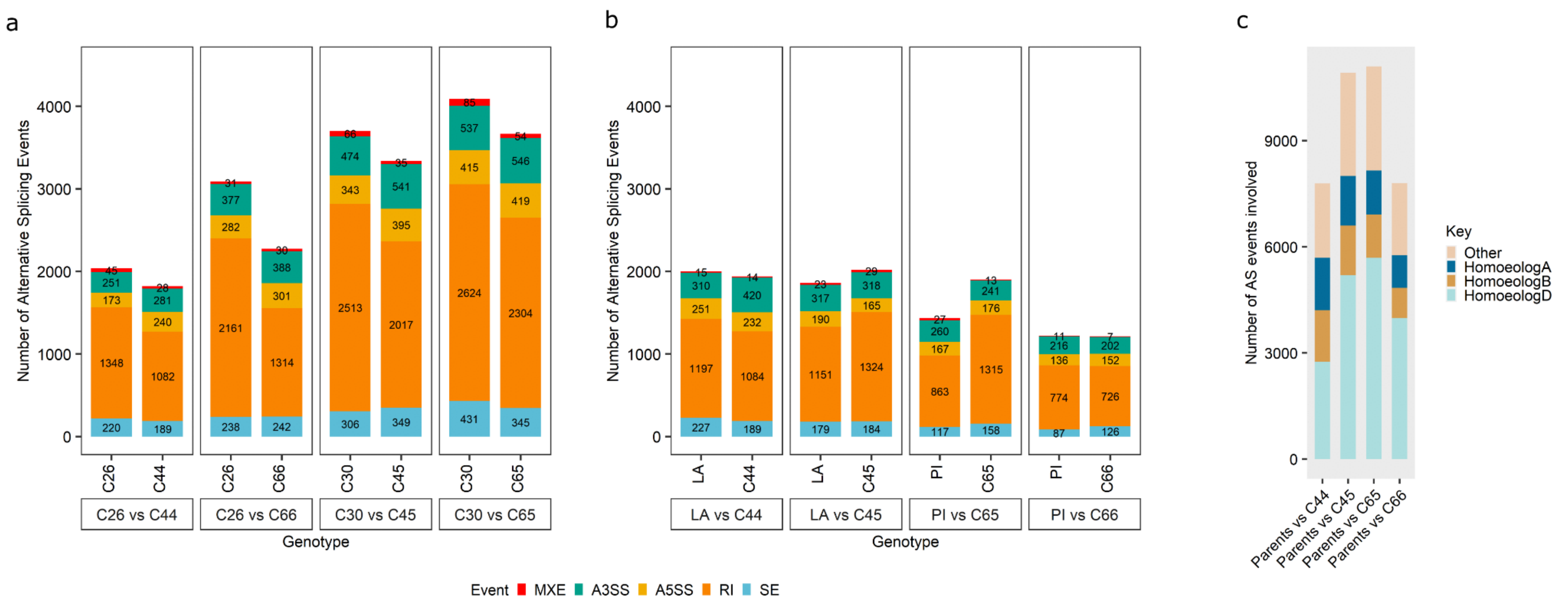

Supplementary Figure 22. Number of alternative splicing events detected in the Diploid/Tetraploid parent vs SHW comparison in head at boot stage.

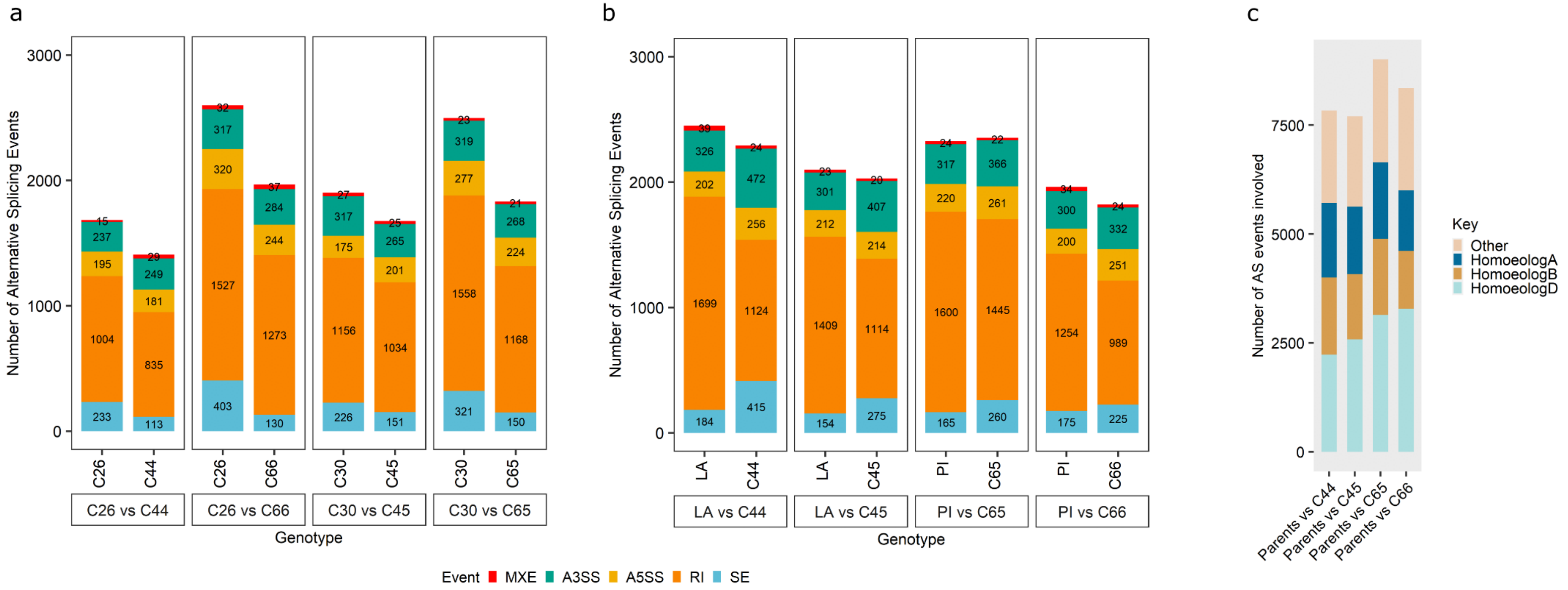

Supplementary Figure 23. Number of alternative splicing events detected in the Diploid/Tetraploid parent vs SHW comparison in glume.

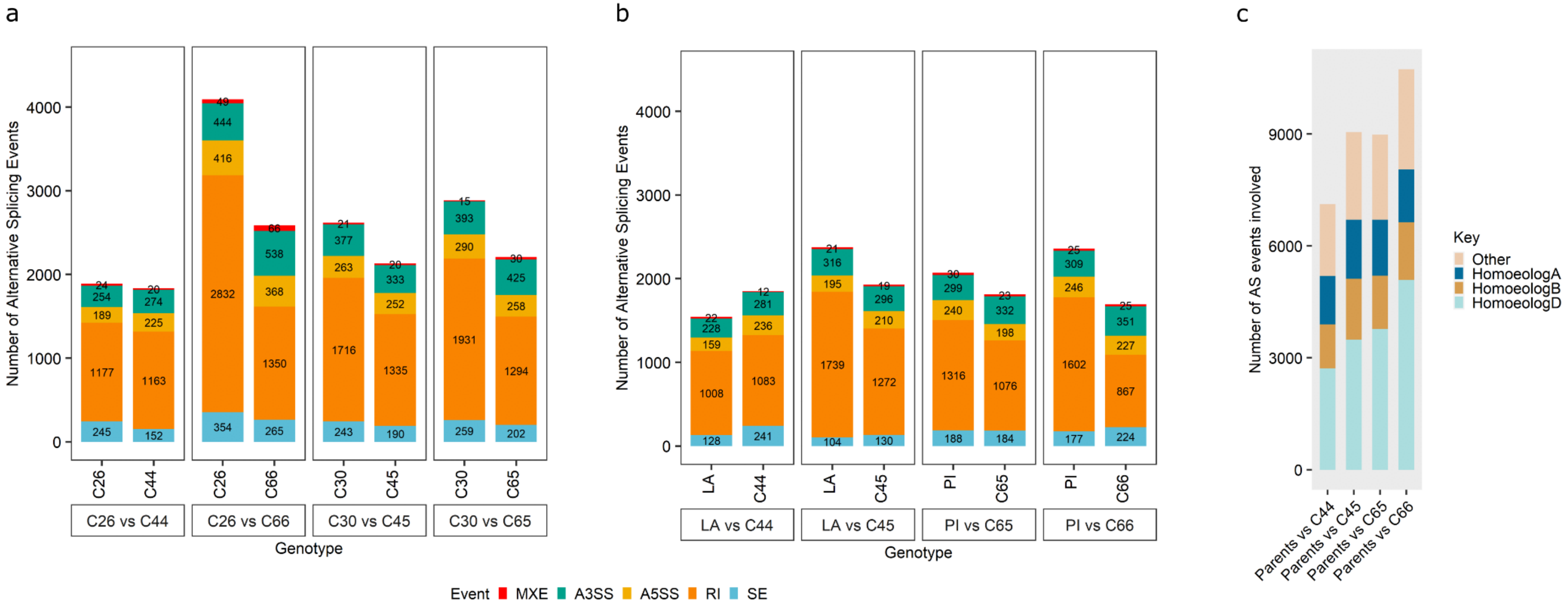

Supplementary Figure 24. Number of alternative splicing events detected in the Diploid/Tetraploid parent vs SHW comparison in hypocotyl.

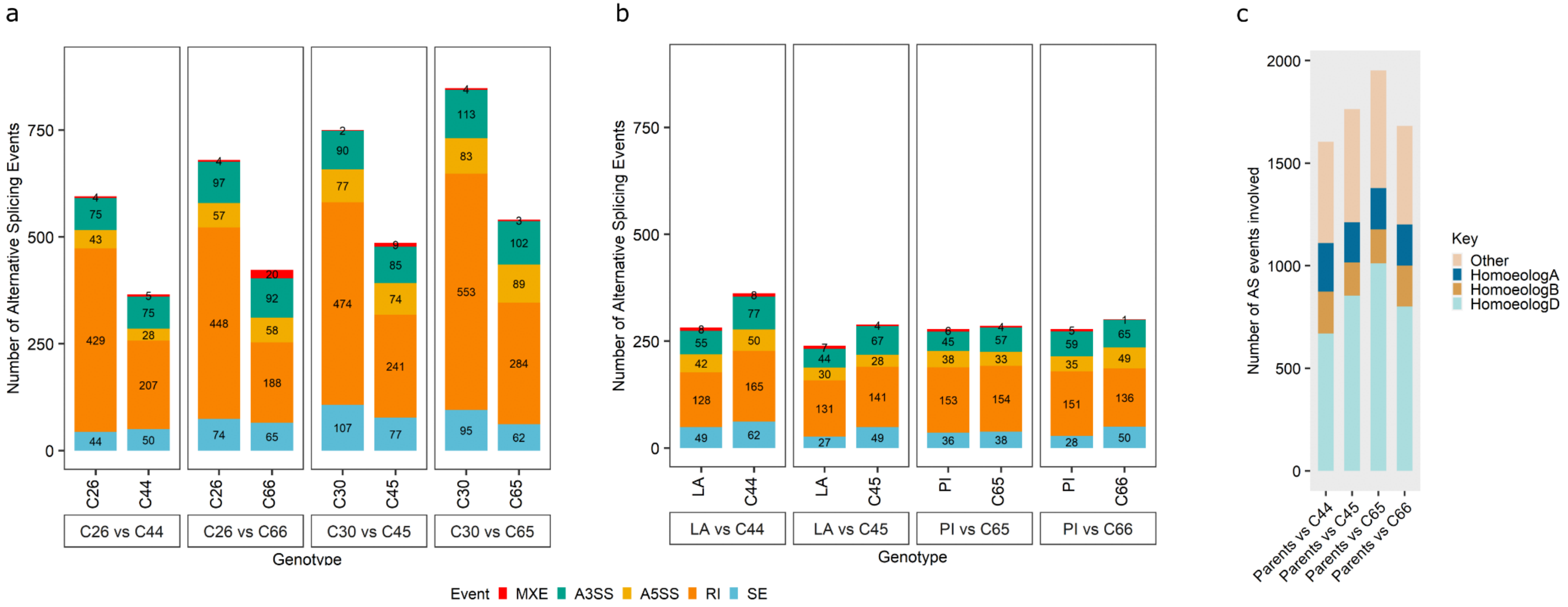

Supplementary Figure 25. Number of alternative splicing events detected in the Diploid/Tetraploid parent vs SHW comparison in pistil-when anthers are green and immature.

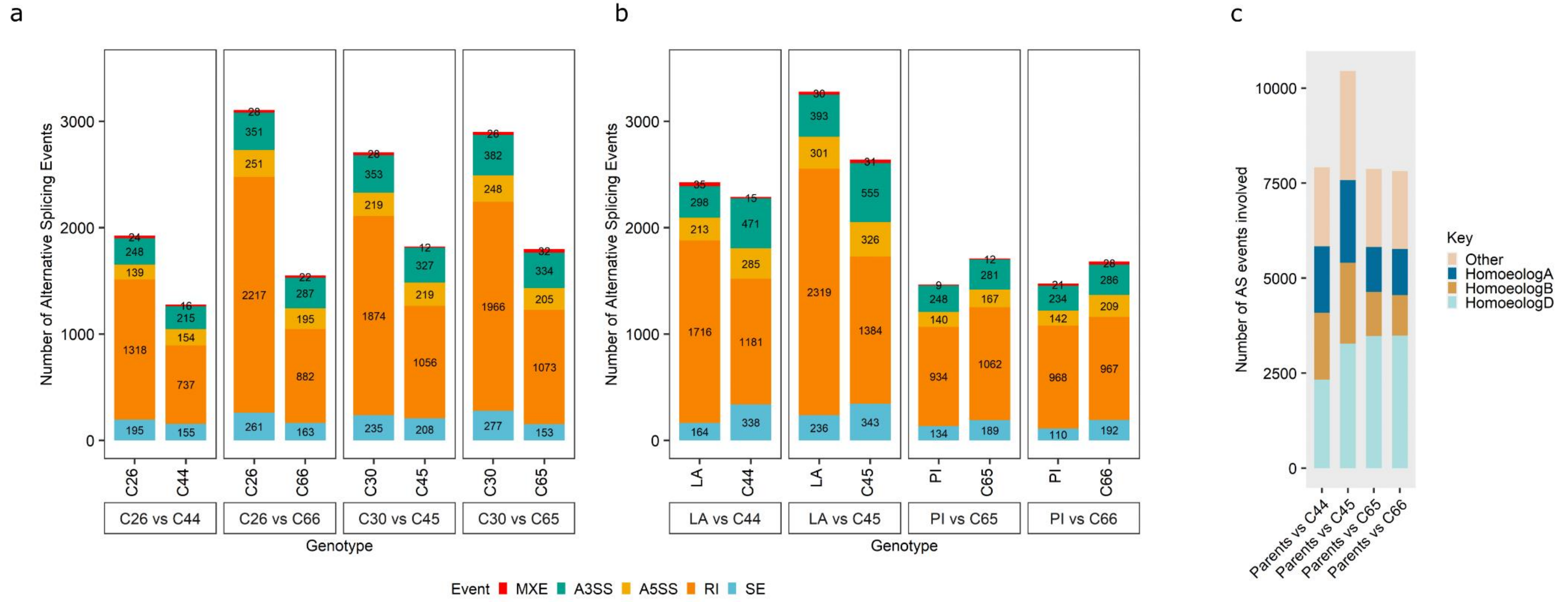

Supplementary Figure 26. Number of alternative splicing events detected in the Diploid/Tetraploid parent vs SHW comparison in pistil-when anthers are yellow and just prior to dehiscence.

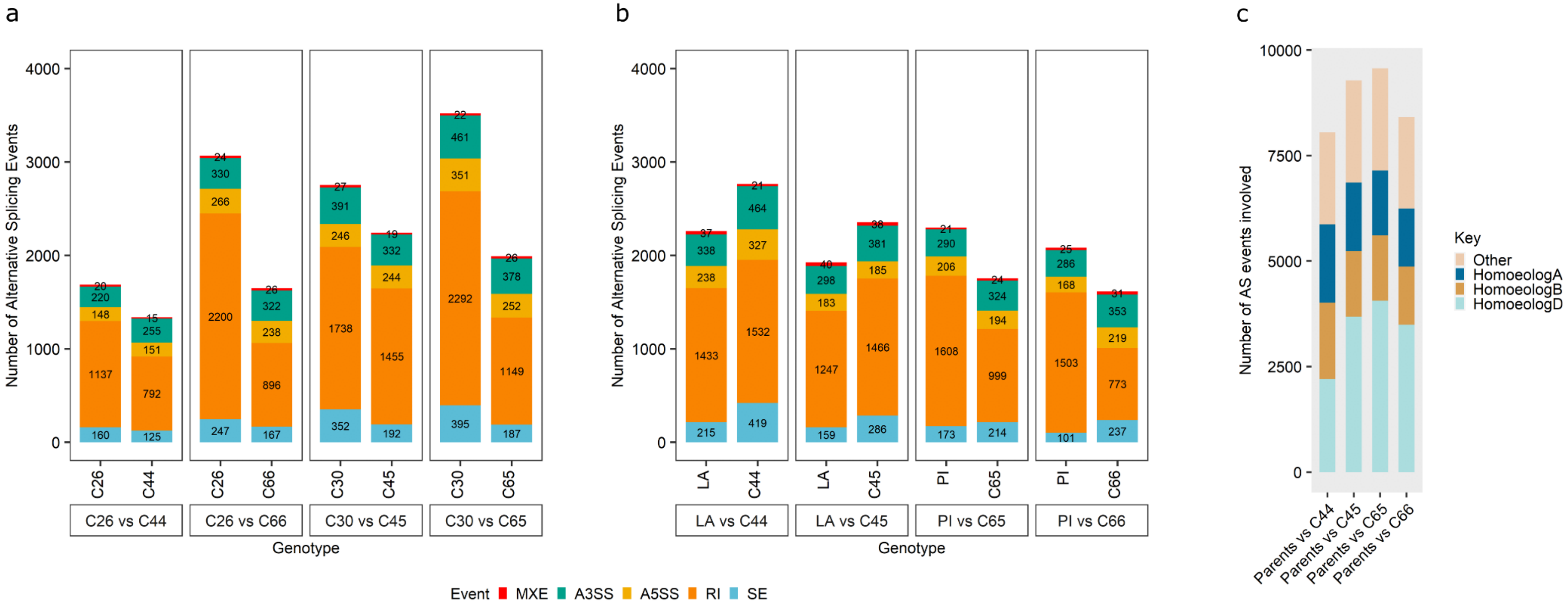

Supplementary Figure 27. Number of alternative splicing events detected in the Diploid/Tetraploid parent vs SHW comparison in palea+lemma.

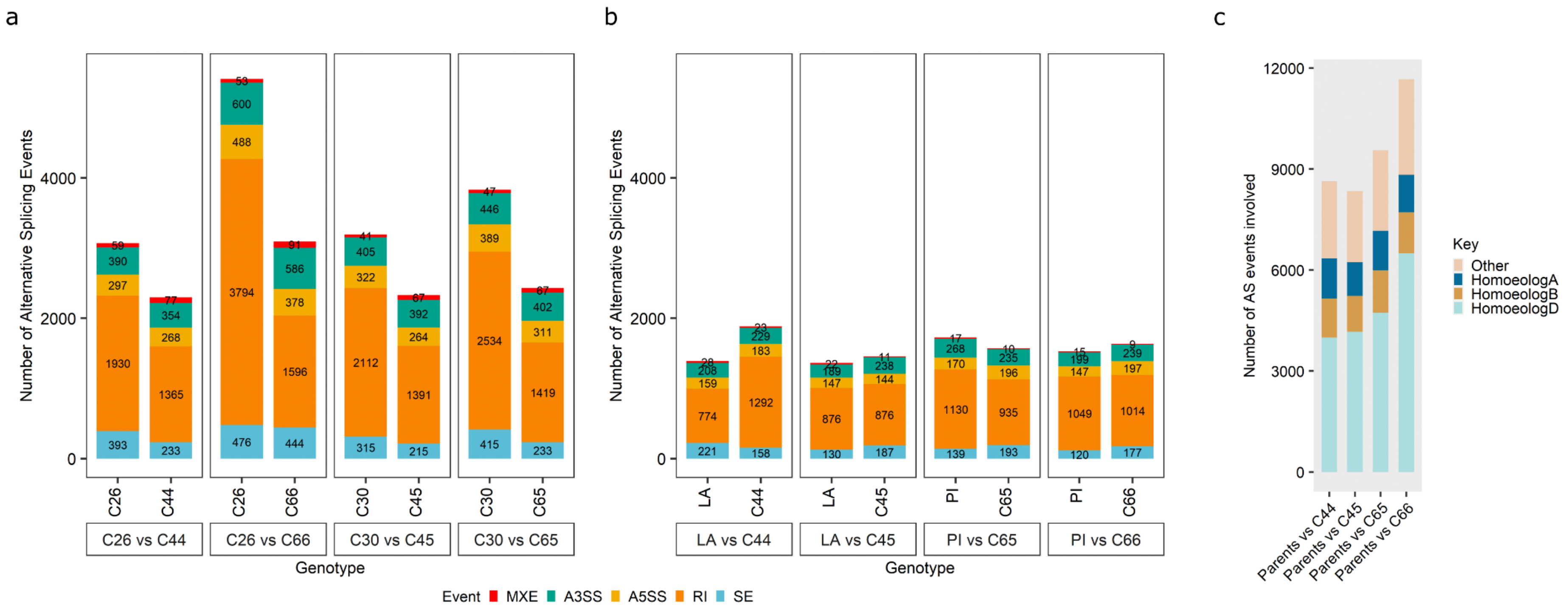

Supplementary Figure 28. Number of alternative splicing events detected in the Diploid/Tetraploid parent vs SHW comparison in root.
